# Optimization of VE607 to generate analogs with improved neutralization activities against SARS-CoV-2 variants

**DOI:** 10.1128/jvi.01034-25

**Published:** 2025-10-13

**Authors:** Shilei Ding, Derek Yang, Irfan Ullah, Ling Niu, Matthew Unger, Marco A. Díaz-Salinas, Monika Chandravanshi, Fei Zhou, Guillaume Beaudoin-Bussières, Mehdi Benlarbi, William D. Tolbert, Keon-Woong Yoon, Ruixue Xu, Geneviève Laroche, Fleur Gaudette, Abraham J. Morton, Zabrina C. Lang, Anna Son, Cameron Abrams, Marceline Côté, Amos B. Smith, Rick K. Huang, Doreen Matthies, James B. Munro, Marzena Pazgier, Pradeep D. Uchil, Andrés Finzi

**Affiliations:** 1 Centre de Recherche du CHUM, Montreal, Québec, Canada; 2 Department of Chemistry, School of Arts and Sciences, University of Pennsylvania, Philadelphia, Pennsylvania, USA; 3 Department of Microbial Pathogenesis, Yale University, School of Medicine, New Haven, Connecticut, USA; 4 Infectious Diseases Division, Department of Medicine, Uniformed Services University of the Health Sciences, Bethesda, Maryland, USA; 5 Department of Microbiology, UMass Chan Medical School, Worcester, Massachusetts, USA; 6 Unit on Structural Biology, Division of Basic and Translational Biophysics, Eunice Kennedy Shriver National Institute of Child Health and Human Development, NIH, Bethesda, Maryland, USA; 7 Département de Microbiologie, Infectiologie et Immunologie, Université de Montréal, Montreal, Québec, Canada; 8 Department of Biochemistry, Microbiology and Immunology, Centre for Infection, Immunity and Inflammation, University of Ottawa, Ottawa, Ontario, Canada; 9 Laboratory of Cell Biology, National Cancer Institute, NIH 313607 , Bethesda, Maryland, USA; 10 Department of Chemical and Biological Engineering, Drexel University, Philadelphia, Pennsylvania, USA; 11 Department of Biochemistry and Molecular Biotechnology, UMass Chan Medical School, Worcester, Massachusetts, USA; The Ohio State University, Columbus, Ohio, USA

**Keywords:** SARS-CoV-2, VE607, CV3-13, neutralization, smFRET, cryo-EM, K18-hACE2 transgenic mice

## Abstract

Severe acute respiratory syndrome coronavirus 2 (SARS-CoV-2) infection remains a threat to human health, particularly among immunocompromised and elderly individuals, given their heightened vulnerability to coronavirus disease 2019 (COVID-19)-associated morbidity and mortality. Recently, omicron subvariants such as KP.3.1.1 and XEC have emerged with an enhanced ability to evade humoral immunity. The development of new strategies against these variants of concern remains an intense area of research. The small molecule VE607 is an entry inhibitor that targets the Spike glycoprotein and delays virus spread *in vivo*. To improve the potency of this new class of SARS-CoV-2 entry inhibitors, we generated and characterized VE607 analogs and identified candidates with enhanced activity against variants, including KP.3.1.1 and XEC. Promising analogs exhibited higher inhibitory potency than the original compound and stabilized the receptor-binding domain in its “up” conformation. Among these, DY-III-281 also reduced viral burden and delayed death in SARS-CoV-2-challenged K18-hACE2 transgenic mice. Furthermore, combining DY-III-281 with a non-neutralizing antibody engineered for Fc-enhanced functions exhibited an additive effect in reducing SARS-CoV-2-induced disease burden in mice. Our findings support the continued development of small-molecule entry inhibitors, alone or in combination with antibody-based therapies, as a promising strategy to counteract emerging SARS-CoV-2 variants.

**IMPORTANCE:**

Mutations in the Spike glycoprotein drive viral evolution and confer resistance to current vaccines and some therapeutic interventions against severe acute respiratory syndrome coronavirus 2 (SARS-CoV-2). Here, we report new analogs of the SARS-CoV-2 small-molecule entry inhibitor VE607. These analogs exhibited improved potency against emerging SARS-CoV-2 variants, including KP.3.1.1 and XEC. One analog, DY-III-281, delayed viral replication in SARS-CoV-2_WA1_-challenged K18-hACE2 transgenic mice, suggesting that small-molecule compounds targeting viral entry might be useful in fighting evolving SARS-CoV-2 variants.

## INTRODUCTION

Severe acute respiratory syndrome coronavirus 2 (SARS-CoV-2) and its variants remain a constant threat, particularly for immunocompromised individuals, whose inherent immunity is not sufficient to clear the virus and therefore have a greater risk of developing prolonged infection (1–5). Emerging variants arise mostly from the accumulation of mutations in the Spike glycoprotein (S). JN.1 subvariants (KP.3.1.1, XEC, LP.8.1, etc.) and a new recombinant variant NB.1.8.1 currently represent the vast majority of newly reported cases (6). Mutations in the Spike glycoprotein, especially the more than 60 mutations found in recent Omicron subvariants, have enabled the virus to better escape protective antibodies generated by infection and vaccination (7, 8). Repeated breakthrough infections compounded by vaccine hesitancy further heighten the risk of long coronavirus disease (COVID) and contribute to a growing public health burden.

Most neutralizing Abs generated either from vaccination or infection target the receptor-binding domain (RBD) or the N-terminal domain (NTD) of the S1 subunit of S, but some also target the S2 subunit, which is the most conserved region of the SARS-CoV-2 S (9–11). Therapeutic monoclonal antibodies (mAbs) were developed for the treatment of infection by preventing virus spread through neutralization (12, 13) and Fc-mediated effector functions (14–19). The potential of these antibodies in therapeutic or prophylaxis settings was first tested in animal models (14, 20–28). These models have included non-human primates, hamsters, B6, and K18-hACE2 mice (29). Challenge studies in K18-hACE2 transgenic mice with protective Abs have revealed that Abs require both neutralization and Fc-effector functions to treat or prevent SARS-CoV-2 infections (14, 20).

Small-molecule inhibitors targeting different viral proteins have been developed and approved to treat SARS-CoV-2 infections (30–32). At the beginning of the COVID-19 pandemic, the Food and Drug Administration approved Remdesivir to treat SARS-CoV-2 infection (33, 34), which was originally discovered for the treatment of hepatitis C virus and Ebola virus (35, 36). New drugs such as Paxlovid (a combination of a protease inhibitor, nirmatrelvir, and the Cyp3A inhibitor ritonavir) were approved later (37–39). Because of the relatively short phylogenetic distance between SARS-CoV-1 and SARS-CoV-2, we also thought of repurposing small-molecule inhibitors previously developed against SARS-CoV-1. During the SARS pandemic, an entry inhibitor targeting the viral Spike, VE607, was reported (40, 41). We previously reported that VE607 inhibits SARS-CoV-2 entry by stabilizing the Spike in the RBD "up" conformation, thereby limiting infection by multiple variants, including several Omicron VOCs and reduced viral burden in K18-hACE2 transgenic mice (42). VE607 is a mixture of three stereochemical isomers composed of (S,S)-VE607, (R,S)-VE607, and (R,R)-VE607 in a ratio of 1:2:1, respectively. (R,R)-VE607 presented a slightly better neutralization (42) and was therefore selected as the basis of our medicinal chemistry efforts.

To advance this class of SARS-CoV-2 entry inhibitors, we synthesized and profiled a panel of VE607-derived analogs. This led to the identification of compounds with improved potency against circulating variants, such as KP.3.1.1 and XEC. Select analogs demonstrated superior inhibitory activity compared to the parent molecule and promoted the “up” conformation of the spike RBD. Notably, a VE607 analog, DY-III-281, not only decreased virus burden but also extended survival in SARS-CoV-2-infected K18-hACE2 mice. Co-administration of DY-III-281 with a non-neutralizing antibody CV3-13 engineered for enhanced Fc effector function further mitigated disease severity, suggesting an additive treatment benefit. These results underscore the potential of combining optimized small-molecule inhibitors with antibody-based approaches to address ongoing viral evolution.

## RESULTS

### VE607 analogs

**Fig 1:**
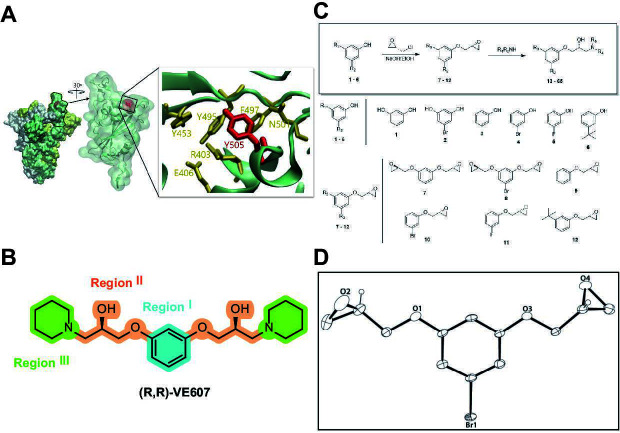
VE607 analogs. (**A**) Putative VE607-binding site on the SARS-CoV-2 Spike. (Left) Protein Data Bank structure 7N0G with RBD of chain A outlined and position of Y505 indicated by a red sphere. (Middle) Tertiary structure of RBD with Y505 indicated in red. (Right) Details of the structure of the neighborhood of Y505. (**B**) The previously synthesized hit compound (R,R)-VE607 color-coded by chemical regions for structure-activity relationship study exploration. (**C**) Chemical synthesis of VE607 and its analogs. (**D**) ORTEP representation of the X-ray structure of 8.

Our medicinal chemistry design, focused on the (R,R)-VE607 stereoisomer, sought to understand the structural requirements for activity and to improve potaency and pharmaceutical properties. From our previous *in silico* studies (42), we identified a potential binding site for VE607 where it interacted with RBD residue Tyr 505 (Fig. 1A). Therefore, in one series of analogs **15–25**, we explored the chemical space around the proposed binding site by modifying region III of (R,R)-VE607 to understand what types of functional groups and substituents were tolerated at this position (Fig. 1B). Other analogs prepared included those with addition of a bromine atom on the aromatic ring **26–41** and truncated analogs **42–65** (Table 1).

**TABLE 1:** IC_50_ value of synthesized compounds against tested pseudoviruses^*a*^

Compound	Name	Structure	IC_50_ VSV-G (µM)	IC_50_ SARS-CoV-1 (µM)	IC_50_ SARS-CoV-2 (µM)	cLogD	Ligand efficiency
VE607	VE607 (racemic)	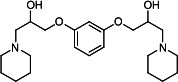	100	0.74	2.66	−2.81	0.27
(R,R)-VE607	(R,R)-VE607	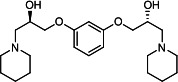	100	0.8	0.98	−2.81	0.29
**15**	DY-III-277	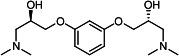	70.7	2.54	1.64	−2.26	0.36
**16**	DY-III-279	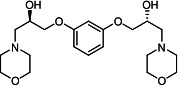	100	100	100	−0.76	0.19
**17**	DY-III-290	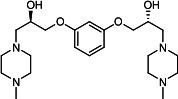	74.9	19.87	1.76	−4.24	0.26
**18^*b*^**	AS-I-020	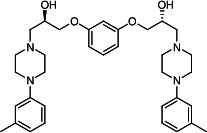	Nd^*c*^	Nd	Nd	Nd	Nd
**19**	AS-I-021	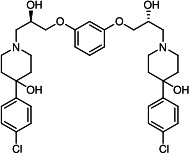	1.35	1.91	0.52	1.61	0.18
**20**	AS-I-013	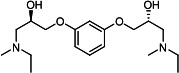	100	0.57	1.18	−2.46	0.34
**21**	AS-I-016	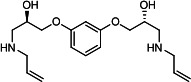	100	41.15	47.85	−1.45	0.25
**22**	AS-I-017	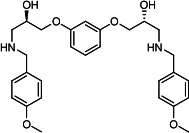	2.85	1.85	0.57	1.00	0.24
**23^*b*^**	AS-I-018	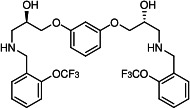	Nd	Nd	Nd	Nd	Nd
**24**	AS-I-019	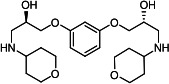	1.38	100	0.38	−2.72	0.29
**25**	AS-I-011	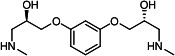	2.69	0.45	100	−2.52	0.27
**26**	DY-III-272	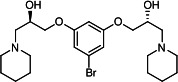	2.39	0.3814	0.443	−1.94	0.29
**27**	**DY-III-281**	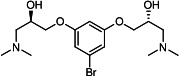	52.36	0.625	0.437	−1.39	0.36
**28**	DY-III-278	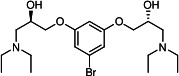	100	17.51	1.79	−2.55	0.30
**29**	DY-III-283	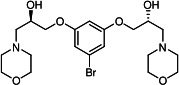	100	30.51	2.65	0.11	0.25
**30**	**DY-III-287**	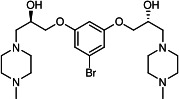	80.84	1.98	1.48	−3.38	0.25
**31^*b*^**	DY-IV-067	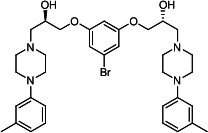	Nd	Nd	Nd	Nd	Nd
**32^*b*^**	DY-IV-068	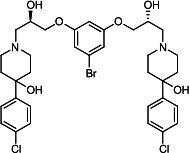	Nd	Nd	Nd	Nd	Nd
**33**	**DY-IV-048**	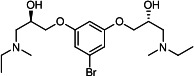	100	1.44	1.71	−1.59	0.30
**34**	DY-IV-050	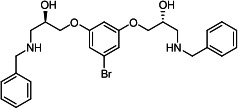	4.71	0.34	0.49	2.14	0.25
**35**	DY-IV-051	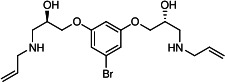	26.39	0.62	0.7	−0.58	0.32
**36**	DY-IV-052	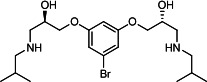	5.05	0.22	0.32	−0.91	0.32
**37**	DY-IV-053	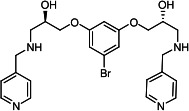	3.89	1.29	1.37	1.51	0.24
**38**	DY-IV-063	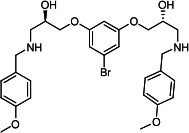	4.09	0.66	1.03	1.87	0.21
**39^*b*^**	DY-IV-064	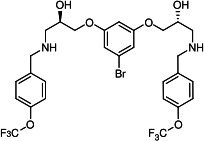	Nd	Nd	Nd	Nd	Nd
**40**	DY-IV-066	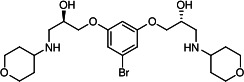	100	0.6	3.63	−1.85	0.23
**41**	DY-IV-044	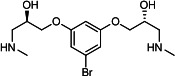	100	0.31	1.17	−1.65	0.37
**42**	DY-III-273	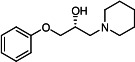	100	0.912	1.35	−0.49	0.47
**43**	AS-I-049	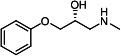	100	62.58	100	−0.35	0.42
**44**	AS-I-043	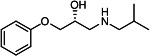	100	5.95	100	0.02	0.34
**45**	AS-I-042	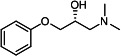	100	13.88	100	−0.22	0.39
**46**	AS-I-070	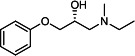	100	100	100	−1.20	0.30
**47**	AS-I-050	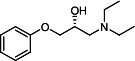	100	100	100	−0.36	0.34
**48**	AS-I-058	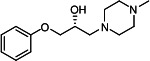	100	100	100	−1.20	0.30
**49**	AS-I-081	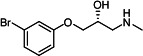	100	100	100	0.49	0.36
**50**	AS-I-082	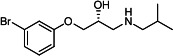	100	100	100	0.87	0.30
**51**	AS-I-077	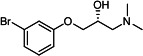	100	17.28	100	0.62	0.34
**52**	AS-I-078	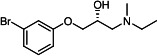	100	51.77	100	0.53	0.32
**53**	AS-I-086	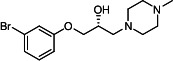	100	100	100	−0.37	0.27
**54**	AS-I-079	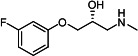	100	54.96	100	−0.24	0.39
**55**	AS-I-080	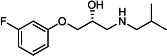	100	100	100	0.13	0.32
**56**	AS-I-072	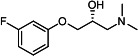	100	19.51	100	−0.11	0.36
**57**	AS-I-073	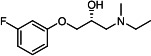	100	6.17	100	−0.21	0.34
**58**	AS-I-083	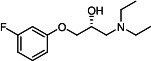	100	100	100	−0.26	0.32
**59**	AS-I-084	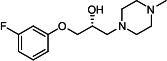	100	100	100	−1.11	0.29
**60**	AS-I-060	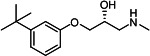	100	45.54	100	1.68	0.32
**61**	AS-I-048	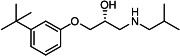	100	73.7	100	2.05	0.27
**62**	AS-I-047	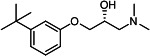	100	0.934	84.67	1.81	0.31
**63**	AS-I-071	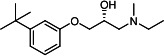	100	4.66	100	1.71	0.29
**64**	AS-I-061	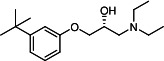	100	57.35	100	1.66	0.27
**65**	AS-I-059	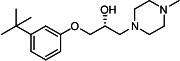	100	22.01	45.66	0.63	0.27

^*a*^ Highlighted in bold are analogs that were further tested in this study.

^*b*^ Insoluble in phosphate-buffered saline (PBS).

^*c*^ Nd, not determined.

To access the various VE607 analogs, we started with aryl alcohols **1–6** (Fig. 1C). To these alcohols, we added (S)-epichlorohydrin and an ethanolic solution of NaOH/EtOH in appropriate equivalents to form either the mono- or di-addition epoxides, **7–12** (Fig. 1C). The enantioselectivity of the synthesis of these epoxides was confirmed by chiral supercritical fluid chromatography. Additionally, the absolute stereochemistry of **8** was determined by single-crystal X-ray diffraction analysis (Fig. 1D). The corresponding epoxides were opened by a series of amines to yield the final VE607 analogs, **13–65** (Table 1; Supplemental material).

We tested all 64 VE607 analogs (Table 1) to verify the neutralizing capacity against pseudoviral particles bearing the Spike glycoproteins from SARS-CoV-1 and SARS-CoV-2. We used pseudotypes with the G glycoprotein of VSV (VSV-G) as a specificity control. Of the compounds that were synthesized, the identity of the amino function had the most impact on non-specificity, as measured by inhibition of VSV-G pseudotyped viruses, and less so on the inhibition of infectivity with SARS-CoV-2 or SARS-CoV-1 Spike pseudoviruses. Particularly, lipophilic secondary amines (region III in Fig. 1B) that resulted in tertiary amine analogs with a calculated log of the distribution coefficient (cLogD) at pH 7.4 of greater than 1.0 resulted in non-specific inhibition of VSV-G. Additionally, while the tertiary amine analogs showed mostly specific neutralization to coronavirus envelopes, secondary amines exhibited broad non-specificity regardless of cLogD. For example, the dimethylamine **15** (IC_50_ VSV-G: 70.7 µM) showed specific inhibition of coronaviruses; however, when one of the terminal methyl groups was removed to yield a methylamine in compound **25** (IC_50_ VSV-G: 2.69 µM), the compound showed non-specific antiviral properties.

A derivative of (R,R)-VE607 (obtained from **2**) with a brominated benzene at region I **26** was synthesized to obtain single-crystal X-ray diffraction data as well as facilitate co-crystallization with the viral Spike using cryo-EM. This compound showed better neutralization capacity (IC_50_ SARS-CoV-2: 0.44 µM) than its parent compound, (R,R)-VE607, (IC_50_ SARS-CoV-2: 0.98 µM). As such, a series of analogs bearing a 3-bromo substituent on the central aromatic core (region I) was synthesized as congeners of the previous resorcinol scaffold, **26–41**. Similar trends that were observed in the resorcinol backbone were mirrored in the 5-bromoresorcinol backbone. Notably, secondary amines still showed broad non-specific inhibition of VSV-G pseudotypes, and highly lipophilic amine R groups similarly resulted in non-specificity. However, by modulating the amine groups to be smaller in size, we could take advantage of the increase in binding affinity afforded by the bromine while also specifically targeting coronavirus spike proteins. This series of modifications resulted in compounds like **27** (DY-III-281) with a superior neutralization capability against both SARS-CoV-1-S (IC_50_: 0.62 µM) and SARS-CoV-2-S (IC_50_: 0.44 µM) pseudoviruses.

Given the C2 symmetry of these analogs, we were then curious to see whether both arms were essential to the neutralization capabilities of these compounds. As such, we started from phenol **3** and synthesized compound **42**. The antiviral assays showed that this compound was active (IC_50_ SARS-CoV-2: 1.35 µM) and had a remarkable ligand efficiency (LE) of 0.47 (Table 1). Therefore, we pursued a series of these compounds starting from phenolic derivatives **9–12** (Supplemental material). Unfortunately, many of these compounds showed no activity against SARS-CoV-2, and therefore attention was turned back toward the symmetric two-armed derivatives, indicating that the dual-functional motive is critical for viral neutralization.

**Fig 2:**
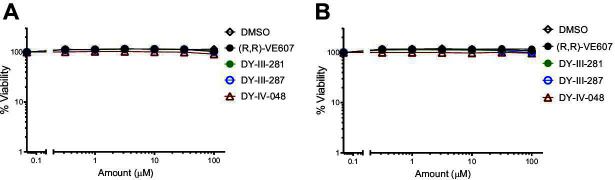
VE607 analogs are not cytotoxic. Indicated serial diluted compounds (from 0 to 100 µM) (R,R)-VE607, DY-III-281, DY-III-287, DY-IV-048, or same volume of dimethyl sulfoxide (DMSO) were applied to test the toxicity on 293T-ACE2 (**A**) or Vero-E6 cells (**B**), which was measured by CellTiter-Glo One Solution Assay (Promega). Data represent the average of at least three independent experiments ± SEM.

From this brief structure-activity relationship (SAR) study of VE607 analogs, three compounds emerged as potential lead compounds, **27** (DY-III-281, IC_50_ SARS-CoV-1: 0.625 µM, IC_50_ SARS-CoV-2: 0.437 µM, LE: 0.36), **30** (DY-III-287, IC_50_ SARS-CoV-1: 1.98 µM, IC_50_ SARS-CoV-2: 1.48 µM, LE: 0.25), and **33** (DY-IV-048, IC_50_ SARS-CoV-1: 1.44 µM, IC_50_ SARS-CoV-2: 1.71 µM, LE: 0.30), and were selected to undergo further *in vitro* and *in vivo* testing. Of note, these compounds showed no toxicity in 293T-ACE2 and Vero-E6 cells at concentrations up to 100 µM (Fig. 2).

### Inhibition of viral infection

**Fig 3:**
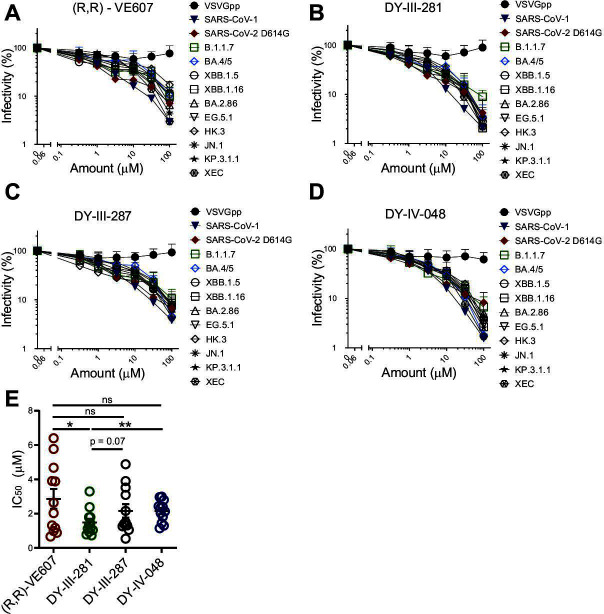
VE607-derived compounds inhibit infection of SARS-CoV-2 variants pseudoviral particles. Pseudoviral particles carrying VSV-G, SARS-CoV-1 S, or S from SARS-CoV-2 variants were produced from transfected 293T cells. Serially diluted compounds (R,R)-VE607 (**A**), DY-III-281 (**B**), DY-III-287 (**C**), and DY-IV-048 (**D**) were applied to inhibit the infection of pseudoviruses in 293T-hACE2 cells. Virus infectivity in the presence of compounds was shown as the percentage of that without a compound. IC_50_ of the compounds against pseudoviruses carrying tested SARS-CoV-2 S were calculated and shown in (**E**). Data represent the average of at least three independent experiments ± SEM; Statistical significance was tested using an unpaired t-test (*P < 0.05; **P < 0.01; ns, not significant).

To compare the neutralizing capacity of (R,R)-VE607, **27** (DY-III-281), **30** (DY-III-287), and **33** (DY-IV-048) on the inhibition of virus infection, pseudoviral particles carrying a luciferase reporter gene and Spike glycoproteins from representative SARS-CoV-2 variants (D614G, B.1.1.7, BA.4/5, XBB.1.5, BA.2.86, EG.5.1, HK.3, JN.1, KP.3.1.1, and XEC) or from SARS-CoV-1 were produced and tested in a standard pseudoviral neutralization assay (43). Pseudoviral particles carrying the VSV-G were used as a specificity control. As shown in Fig. 3, (R,R)-VE607 (Fig. 3A) and the three new analogs: DY-III-281 (Fig. 3B), DY-III-287 (Fig. 3C), and DY-IV-048 (Fig. 3D) neutralized pseudoviral particles carrying SARS-CoV-2 or SARS-CoV-1 Spikes. This neutralization was specific as they did not neutralize pseudoviral particles carrying the G glycoprotein of VSV (VSV-G). Overall, compound DY-III-281 was more potent against the different VOCs Spikes than the lead (R,R)-VE607 inhibitor (Table 2). This could be highlighted when comparing the IC_50_ of the four compounds against the different pseudoviruses tested (Fig. 3E).

**TABLE 2:** IC_50_ value against tested pseudoviruses^*a*^

	IC_50_ (µM)												
	VSVG	SARS-CoV-1	SARS CoV-2 D614G	B.1.1.7	BA.4/5	XBB.1.5	XBB.1.16	BA.2.86	EG.5.1	HK.3	JN.1	KP.3.1.1	XEC
(R,R)-VE607	>100	0.89	0.66	1.17	5.78	0.99	2.03	2.64	1.31	3.88	6.40	4.68	3.90
DY-III-281	>100	0.93	0.78	1.12	1.45	1.04	1.80	3.30	1.13	0.73	1.34	2.38	1.79
DY-III-287	>100	1.31	1.31	1.26	4.88	1.05	1.40	3.52	1.50	0.54	3.90	2.16	3.09
DY-IV-048	>100	1.30	1.15	1.44	2.81	2.43	2.13	2.97	1.82	2.35	2.02	2.99	2.39

^*a*^ Data represent the average of at least three independent experiments.

**Fig 4:**
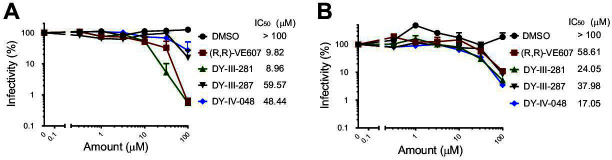
VE607-derived compounds inhibit infection of authentic SARS-CoV-2 viruses. Serially diluted compounds (max 100 µM) (R,R)-VE607, DY-III-281, DY-III-287, or DY-IV-048 were applied to inhibit the infection of authentic SARS-CoV-2 D614G (**A**) or XBB.1 (**B**) in Vero-E6 cells. Virus infectivity in the presence of compounds was shown as the percentage of that without a compound. IC_50_S of the compounds were calculated and are shown beside the graphs. Data represent the average of at least three independent experiments.

We next validated the neutralizing activity of DY-III-281 and DY-IV-048 using authentic SARS-CoV-2 D614G and XBB.1 viruses (44). Briefly, serially diluted (half-Log dilutions) compounds were pre-incubated with the authentic viruses before the addition to Vero-E6 cells in 96-well plates for infection. Small-molecule inhibitors were then added after the removal of the virus-compound mixture, and the cells were incubated for 48–72 hours. Viral infectivity was assessed by intracellular detection of the virus nucleocapsid. As shown in Fig. 4, all analogs effectively blocked infection of Vero-E6 cells by both authentic SARS-CoV-2 D614G (Fig. 4A) and XBB.1 (Fig. 4B), albeit to a different extent. Analog DY-III-281 was more potent than (R,R)-VE607 against SARS-CoV-2 D614G and SARS-CoV-2 XBB.1. To compare the neutralization capacity against pseudovirus and authentic virus, we included a neutralizing antibody in both assays, CV3-25, which inhibits the infection of all current SARS-CoV-2 variants (45). As shown in Fig. S2, IC_50_ of CV3-25 against SARS-CoV-2 D614G Spike pseudotyped virus is lower than that against SARS-CoV-2 D614G authentic virus, indicating the variability of different neutralization assays.

### VE607 analogs promote the RBD-up conformation

**Fig 5:**
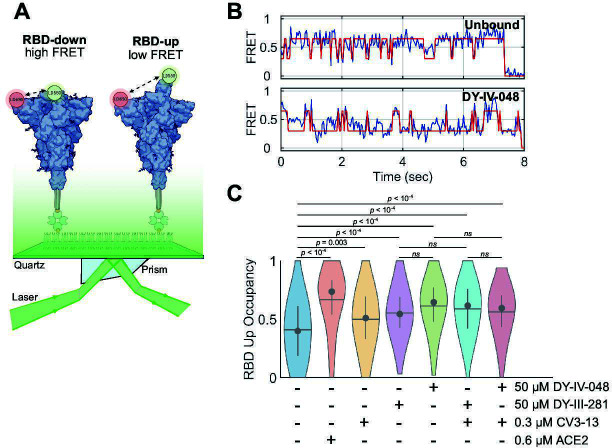
smFRET analysis indicates stabilization of the RBD-up conformation of Spike by VE607 analogs. (**A**) Schematic of the smFRET imaging assay used to evaluate the conformational dynamics of the Spike ectodomain. Donor and acceptor fluorophores, LD555 and LD655, are indicated with green and red circles, respectively. (**B**) Example smFRET trajectories (blue) obtained for unbound, DY-III-281 and DY-IV-048-bound Spike, with idealized trajectory resulting from HMM analysis overlaid in red. (**C**) Violin plots indicating the distribution in RBD-up conformation (low FRET) occupancy. Horizontal lines indicate the mean occupancy; circles and vertical whiskers indicate the median and quantiles, respectively. *P*-values were determined by one-way ANOVA and multiple comparison testing (*ns*, not significant; *P* > 0.05).

Previous work on the viral entry inhibitor (R,R)-VE607 demonstrated that it decreased viral replication in the lungs of K18-hACE2 mice. The decreased replication may have resulted from allosterically blocking ACE2-mediated Spike conformational changes necessary for membrane fusion (42). Here, we sought to determine how the optimized analogs impact Spike conformation. To that end, we implemented a previously described smFRET assay to probe the RBD dynamics of the Spike glycoprotein ectodomain in the absence or presence of ACE2, DY-III-281, DY-IV-048, a non-neutralizing NTD-specific antibody CV3-13 mAb (14), both CV3-13 mAb and DY-III-281, or both CV3-13 mAb and DY-IV-048. A site-specific enzymatic labeling approach was implemented for Spike such that fluorophores were positioned in the NTD and RBD (Fig. 5A). Under all conditions tested, hidden Markov modeling (HMM) of the smFRET trajectories revealed transitions between high- (~0.65) and low- (~0.35) FRET states, which reflect the RBD-down and RBD-up conformations, respectively (Fig. 5B) (46). The impact of DY-III-281 or DY-IV-048 on the fraction of time (occupancy) spent in the RBD-up conformation was assessed by incubating 0.1 µM fluorescently labeled Spike with 0.6 µM ACE2, 50 µM DY-III-281 or DY-IV-048, 0.3 µM CV3-13, or both 50 µM DY-III-281 or DY-IV-048 and 0.3 µM CV3-13, followed by smFRET analysis (Fig. 5C). In the absence of bound ligand, the occupancy of the RBD-up conformation was 40% ± 2%. Incubation with ACE2 increased the RBD-up occupancy to 67% ± 2% (*P* < 10^−4^), consistent with previous work (46). Similarly, DY-III-281 increased the RBD-up occupancy to 55% ± 2% (*P* < 10^−4^), DY-IV-048 increased the RBD-up occupancy to 61% ± 2% (*P* < 10^−4^, Fig. 5C). The increase in RBD-up occupancy upon binding to DY-III-281 or DY-IV-048 is consistent with previous work on the precursor molecule, (R,R)-VE607 (42). The NTD-targeting antibody CV3-13 moderately increased the RBD-up occupancy of Spike to 50% ± 2% (*P* = 0.002), consistent with previous studies of similar antibodies (46, 47). Lastly, the combination of CV3-13 with DY-III-281 or DY-IV-048 increased the RBD-up to 59% ± 2% (*P* < 10^−4^) or 56% ± 2% (*P* < 10^−4^), respectively, which was not significantly different than the RBD-up occupancy in the presence of either ligand independently. These data demonstrate that the new analog shifts the conformational equilibrium of Spike in favor of the RBD-up conformation to an extent comparable to a NTD-targeting antibody.

### A VE607 analog modifies the conformational state of the RBD

To better understand the molecular details of (R,R)-VE607 analogs on the interaction with the SARS-CoV-2 spike, we employed single-particle cryo-electron microscopy (cryo-EM). For our structural studies, we used a soluble preparation of a stabilized, uncleaved SARS-CoV-2 spike trimer, expressed in GnTI^−^ 293F cells. This spike construct, referred to as HPM7, is based on the original Wuhan strain and includes six proline mutations as well as an engineered interprotomer disulfide bond (48). To assess conformational changes in the spike glycoprotein induced specifically by the (R,R)-VE607 analog DY-III-281, we collected two cryo-EM data sets: one for the SARS-CoV-2 spike treated with DY-III-281, and a control (“apo”) data set in which the spike was preincubated with dimethyl sulfoxide (DMSO) at the same concentration used to solubilize DY-III-281, but without the compound itself.

**Fig 6:**
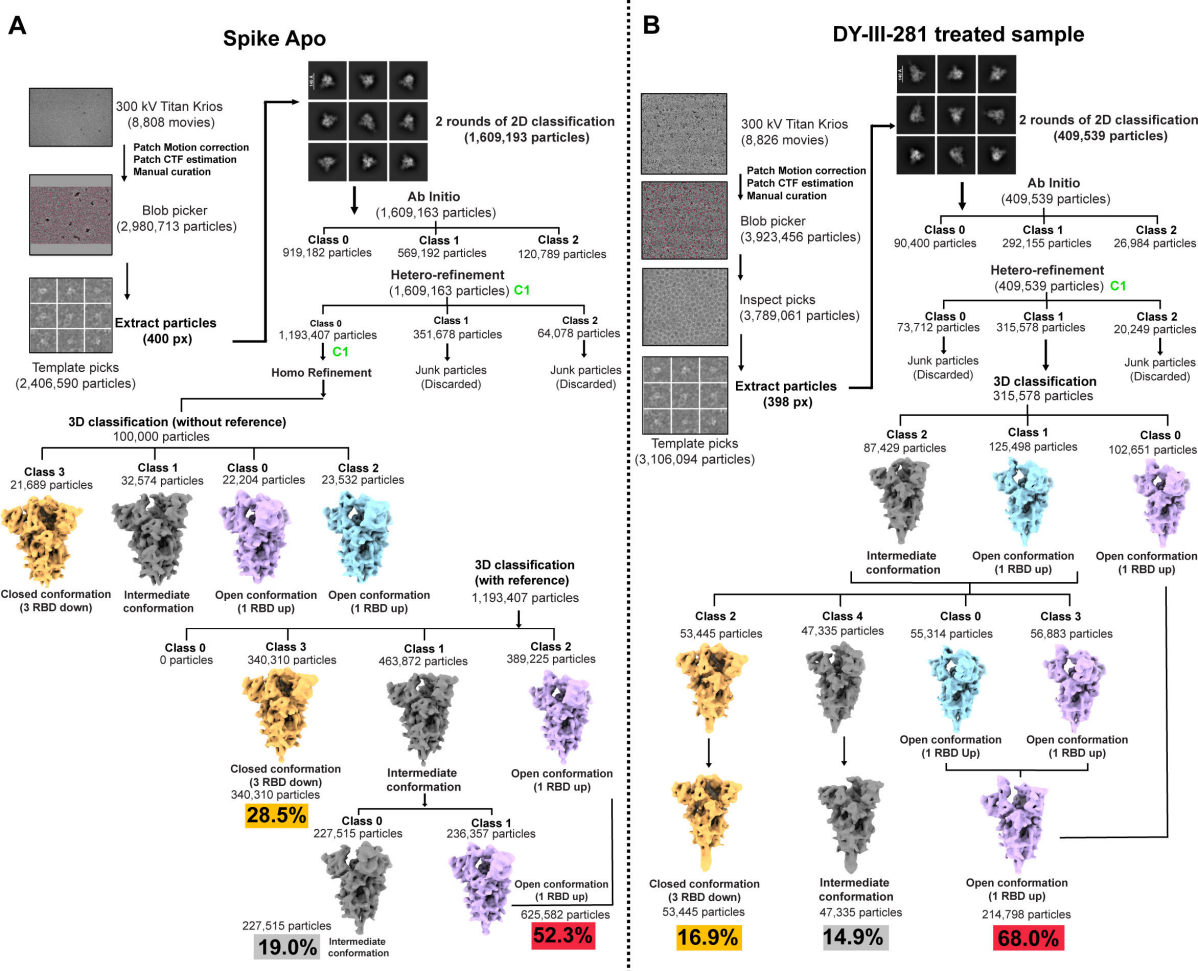
Schematic representation of the Cryo-EM data processing for the apo SARS-CoV-2 spike and SARS-CoV-2 spike treated with DY-III-281. All data processing steps were performed using the software CryoSPARC with C1 symmetry applied throughout all reconstruction and refinement steps. (**A**) A flow chart of data processing for the sample of the apo SARS-CoV-2 spike. (**B**) A flow chart of data processing for the sample of SARS-CoV-2 spike treated with DY-III-281. Particle auto-picking was followed by manual curation and multiple iterative rounds of 2D and 3D classification to eliminate bad particles. Selected high-quality particles were subjected to 3D AutoRefinement to generate the core maps. Subsequently, 3D classification without alignment was performed for the segregation of particles into distinct conformational states of the spike protein. Two processing strategies of 3D classification were followed to obtain a closed and open conformation state of the spike treated with DMSO (left) and DY-III-281 (right). Maps corresponding to closed (orange), open (purple and cyan), and intermediate (gray) conformations are highlighted. Of note, as previously reported, the HPM7 spike expressed in regular HEK 293F cells preferentially adopts the closed (three RBD-down) conformation (48). In contrast, in our apo spike data set, the majority of particles adopt the one-RBD-up open conformation, rather than the closed state observed by others. We speculate that this difference arises from our use of GnTI^−^ 293F cells instead of standard 293F cells, which may influence glycosylation and, consequently, conformational preferences.

Figure 6 schematically illustrates how both data sets were processed to obtain final maps representing the major conformational states adopted by the SARS-CoV-2 spike in each experimental sample. We also quantified the abundance of each conformational class in the data set, calculated as the percentage of particles assigned to each class. In both the apo and DY-III-281-treated data sets, we identified three distinct SARS-CoV-2 conformations: closed (with all three RBDs down), open (with one RBD up), and intermediate (characterized by weak or undefined density for the RBD of one S1/S2 protomer). We proceeded with fitting and refinement of the closed and open conformations from each data set, obtaining structures at 3.14 Å and 3.09 Å for the apo SARS-CoV-2 spike, and 3.56 Å and 3.30 Å for the DY-III-281-treated spike, respectively (Table S1 and Fig. S3).

First, we conducted a thorough inspection of the densities for both the open and closed conformations of the DY-III-281-treated sample to determine whether the DY-III-281 compound could be detected bound to the SARS-CoV-2 spike. However, we were unable to identify any distinct additional density that could be confidently attributed to the compound. This suggests DY-III-281 binding is likely transient, or that the local resolution of the RBDs is insufficient to unambiguously resolve its binding site (Fig. S3). Given the absence of detectable compound-bound density, we next investigated the distribution of conformational states in our DY-III-281-treated and untreated (apo) samples. Although both data sets exhibited the same conformational classes, their relative abundances differed. In the apo spike data set, 28.5% of particles adopted the closed conformation, compared to only 16.9% in the DY-III-281-treated data set. Conversely, the one-RBD-up open conformation accounted for 52.3% of particles in the apo sample (Fig. 6A) and 68.0% of particles in the DY-III-281-treated sample (Fig. 6B). Based on these observations, we propose that DY-III-281 either promotes the transition of the SARS-CoV-2 spike into the one-RBD-up conformation or stabilizes this conformation once adopted. These findings are also consistent with smFRET results in which a different (R,R)-VE607 analog shifted the conformational landscape to the RBD up conformation (Fig. 5), which is considered as energetically favorable upon substrate binding.

**Fig 7:**
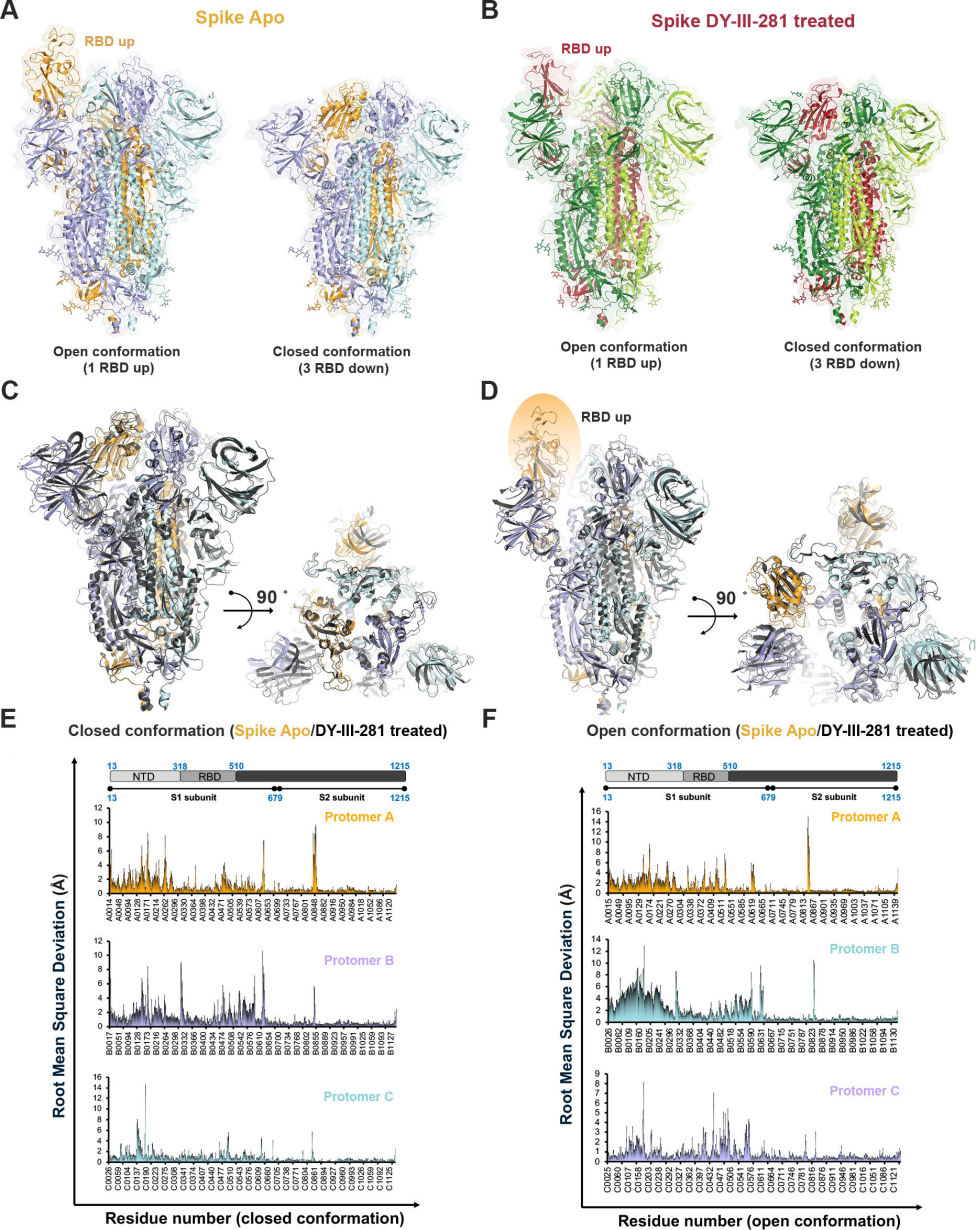
Comparative structure analysis of Apo SARS-CoV-2 spike and SARS-CoV-2 spike treated with DY-III-281. Overall structural comparison of the open and closed conformation of SARS-CoV-2 spike protein in the (**A**) apo and (**B**) DY-III-281-treated spikes, respectively. Each of the three protomers for each conformation is represented in a different color. The protomer with the RBD in the "up" conformation is highlighted in orange for the apo spike and in red for the DY-III-281-treated spike. (**C** and **D**) Structural overlays of the apo and small-molecule-treated spike proteins in the closed and open conformations, respectively. Superpositions were performed based on main chain atoms, and both side and top views are provided for each conformation. (**E** and **F**) RMSD plots indicating major conformational changes, particularly within the NTD and RBD domains of the spike, observed upon comparison of apo and DY-III-281-treated spikes. These changes are shown for both the closed (**E**) and open (**F**) conformations of the spike protein.

Finally, we superimposed the closed conformation (three RBD down) structures of the apo and DY-III-281-treated spike proteins to evaluate structural differences (Fig. 7). The analysis revealed no significant conformational deviation between the two states, with a root-mean-square deviation (RMSD) of 1.63 Å (Fig. 7C). Similarly, superposition of the open conformation (one RBD up) structures from the apo and DY-III-281-treated data sets yielded a Cα RMSD of 2.12 Å (Fig. 7D). To further investigate domain-specific dynamics, we calculated domain-wise RMSD values. While the S2 domain remained relatively stable in both the closed (RMSD: 1.17 Å) and open state (RMSD: 1.26 Å), there was a notable increase in RMSDs corresponding to conformational change in the NTD and RBD domains. Next, we plotted the RMSD plot for each residue to highlight this change in the NTD and RBD regions (Fig. 7E and F). Although the S2 domain remained structurally rigid, the NTD and RBD domains exhibited significant conformational heterogeneity between samples.

### DY-III-281 delays virus spread and decreases viral load in organs from SARS-CoV-2_WA1_-challenged mice

**Fig 8:**
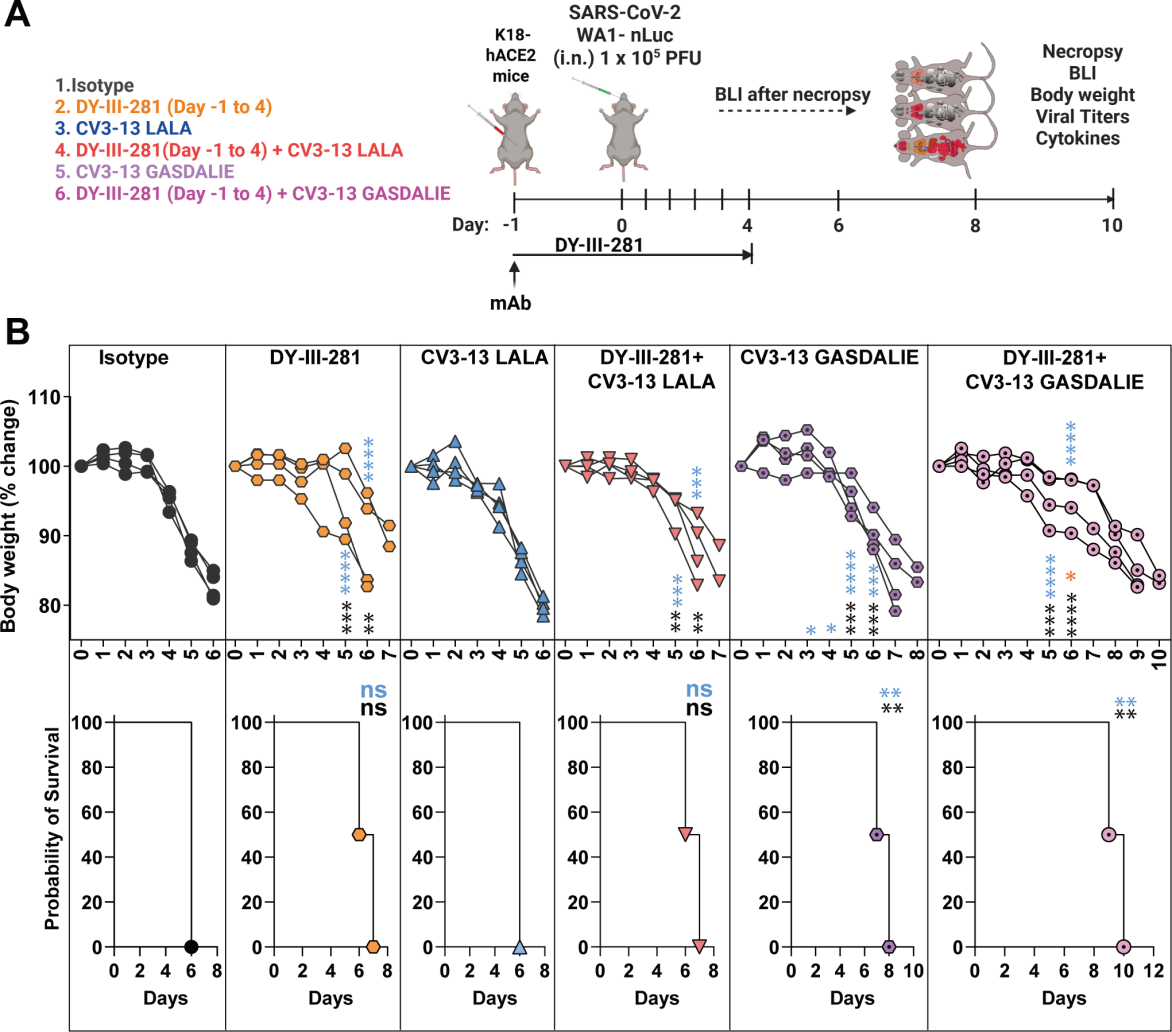
DY-III-281, when combined with CV3-13 GASDALIE, extends the survival of K18-hACE2 mice lethally challenged with SARS-CoV-2_WA1-nLuc_. (**A**) Experimental design for testing the efficacy of DY-III-281 in combination with CV3-13 GASDALIE. Mice received DY-III-281 (25 mg/kg) once daily by intraperitoneal (i.p.) injection starting one day before infection (day −1) and continuing through day 4 post-infection. Antibody treatments (12.5 mg/kg, i.p.), either alone or in combination with DY-III-281, were administered on 1 day prior (day −1) to intranasal (i.n.) challenge with 1 × 10⁵ PFU of SARS-CoV-2_WA1-nLuc_. (**B**) The graph illustrates the changes in body weight over time for K18-hACE2 mice following infection. Each line represents an individual animal, with the initial weight set at 100%. The plots displayed below show Kaplan-Meier survival curves for the experiment described in A. Grouped data in B were analyzed by two-way ANOVA followed by Tukey’s multiple comparison tests. Statistical significance for group comparisons to Vehicle is shown in black, with CV3-13 LALA shown in blue and with DY-III-281 CV3-13 LALA shown in pink. ∗*P* < 0.05; ∗∗*P* < 0.01; ∗∗∗*P* < 0.001; ∗∗∗∗*P* < 0.0001; ns, not significant; mean values ± SD are depicted.

**Fig 9:**
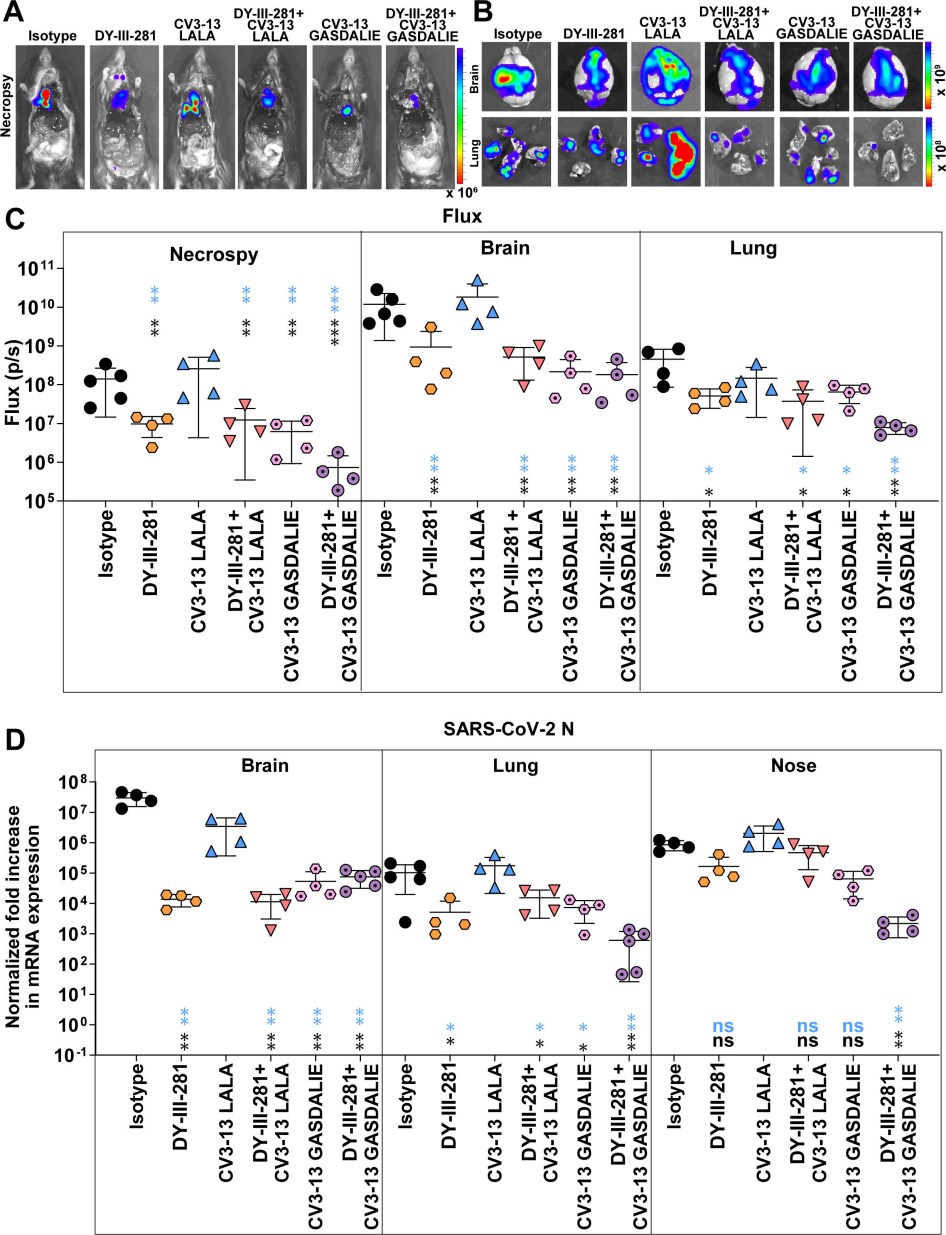
DY-III-281 with CV3-13 GASDALIE reduces viral burden in K18-hACE2 mice challenged with SARS-CoV-2_WA1-nLuc_ (**A–C**) The representative images of whole body, brain, and lung at the time of necropsy and quantification of signals as flux (photons/s) from mice shown in panel A. Scale bar denotes luminescent radiance (p/sec/cm2/sr). (**D**) Changes in SARS-CoV-2 mRNA expression levels were measured in indicated tissues following specified treatment protocols at the time of death after necropsy. The data were standardized against *Gapdh* mRNA expression from the same sample and from uninfected mice after necropsy. The data in (**C and D**) were analyzed by two-way ANOVA followed by Tukey’s multiple comparison tests. Statistical significance for group comparisons to Vehicle is shown in black, with CV3-13 LALA shown in blue. ∗*P* < 0.05; ∗∗*P* < 0.01; ∗∗∗*P* < 0.001; ns, not significant; mean values ± SD are depicted.

**Fig 10:**
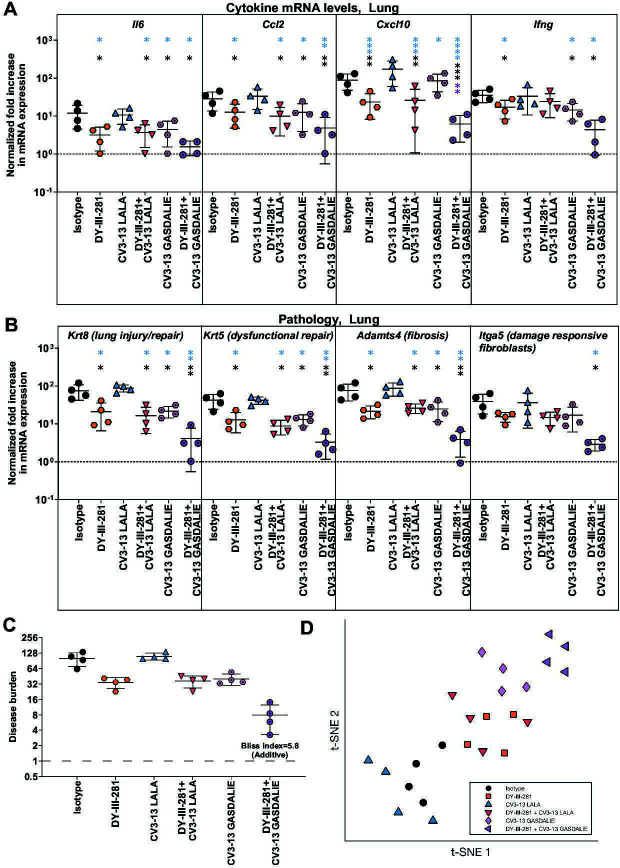
DY-III-281, when combined with CV3-13 GASDALIE, acts additively to reduce disease burden in K18-hACE2 mice challenged with SARS-CoV-2_WA1-nLuc_. (**A–B**) mRNA expression levels of indicated inflammatory cytokines (**A**) and lung pathology markers (**B**) were measured in mouse lung tissues following the specified treatment protocols at the time of death following necropsy. (**C**) Multiparametric disease burden and Bliss index estimation (see Materials and Methods for details) for the indicated groups of mice to compare the efficacies of indicated treatment regimens. Bliss index score of −10 to 10 is considered as an additive effect. (**D**) Visualization of multiparametric disease burden estimations after dimensionality reduction using a t-SNE plot for the indicated treatment regimens. The data were standardized against *Gapdh* mRNA expression from the same sample and from uninfected mice after necropsy in the experiments depicted in Fig. 8A. Data in (**A and B**) were analyzed by two-way ANOVA followed by Tukey’s multiple comparison tests. Statistical significance for group comparisons to Vehicle is shown in black, with CV3-13 LALA shown in blue, with CV3-13 GASDALIE shown in purple. ∗*P* < 0.05; ∗∗*P* < 0.01; ∗∗∗*P* < 0.001; ∗∗∗∗*P* < 0.0001; ns, not significant; mean values ± SD are depicted.

Pharmacokinetic analysis was performed to determine DY-III-281 tissue distribution and concentration decay over time (Fig. S4). K18-hACE2 mice received DY-III-281 or vehicle (DMSO) intraperitoneally at doses of 12.5, 25, and 50 mg/kg, followed by blood collection at 0.5, 2, 8, and 24 hours of intervals and concentrations evaluated by mass spectrometry. In addition, DY-III-281 concentrations in organs, bronchoalveolar lavage fluids (BALF), and nasal wash were evaluated after necropsy at 24 h. Thirty minutes after administering 25 mg/kg, we detected ~1.5 µM of DY-III-281 in plasma, but the levels waned at later time points. DY-III-281 levels were lower in lung, liver, kidney, and spleen compared to plasma, with minimal penetration in the brain (Fig. S4). Because DY-III-281 displayed short plasma retention in PK studies, we evaluated the *in vivo* efficacy by treating K18-hACE2 mice at 25 mg/kg once daily from day –1 to day 4 post-infection with SARS-CoV-2_WA1-nLuc_ (Fig. 8A). While all the mice in this DY-III-281-treated group succumbed to virus-induced mortality, we observed a significant delay in body weight loss and survival was extended by 1 day compared to the control group (Fig. 8B). As is true for many viral infections, combination regimens targeting different steps of virus replication are required for efficient virus clearance. In our previous study, we had demonstrated that a combination of virus-directed (molnupiravir) and host-augmented (convalescent plasma with Fc-effector functions) therapy was successful when the monotherapies on their own failed in preventing SARS-CoV-2-induced mortality (49). In addition, we previously demonstrated that neutralization and Fc-effector functions can be uncoupled and still confer protection against SARS-CoV-2-induced mortality (14). We therefore investigated whether the entry inhibtion provided by DY-III-281 can be modulated by the enhanced Fc-effector functions (GASDALIE mutations) provided by a NTD-binding non-neutralizing antibody CV3-13. K18-hACE2 mice were prophylactically treated with engineered CV3-13 mAb variants carrying LALA (diminished Fc-effector function) or GASDALIE (enhanced Fc-effector function) mutations either alone or in combination with DY-III-281. As expected (14), prophylactic administration of CV3-13 LALA failed to confer protection, as body weight loss and mortality were comparable to those in isotype control-treated mice (Fig. 8B). Consistent with these findings, combining DY-III-281 with CV3-13 LALA did not improve survival or mitigate weight loss beyond the effect of DY-III-281 alone. Prophylaxis with CV3-13 GASDALIE significantly delayed body weight loss and death by 1–2 days compared to control groups of mice (Fig. 8B) (14). Furthermore, the combination of DY-III-281 with CV3-13 GASDALIE significantly reduced body weight loss and extended survival to 10 dpi and was more effective than either intervention alone. Viral load (flux quantification and N mRNA; Fig. 9A through D), inflammatory cytokine, and lung pathology marker mRNA expression at the time of death (Fig. 10A through C) corroborated these findings, demonstrating the superior efficacy of the DY-III-281 combination regimen with CV3-13 GASDALIE to other tested prophylactic administrations. While we observed significantly reduced viral titers in both the brain and lungs of treated mice, the reduction in brain viral loads was not sufficient to achieve complete protection from mortality in this susceptible model. Under these conditions, we were able to observe only delayed death for various treatment regimens. This limited improvement can be attributed to several factors. Based on our pharmacokinetic study results (Fig. S4), relatively low levels of DY-III-281 were detected in the brain compared to other organs. Additionally, viral persistence in the brain was still observed after treatment (Fig. 9), likely due to insufficient drug penetration across the blood-brain barrier. Since K18-hACE2 mice succumb primarily to brain infection, inadequate levels of drug in the brain may explain the modest protective effect despite a significant reduction in viral titers in lungs. Given these limitations of using survival as the sole endpoint, we performed disease burden calculations incorporating seven parameters and dimensionality reduction using t-SNE plots (see Materials and Methods) to comprehensively assess treatment efficacy (Fig. 10C and D). This multi-parameter approach provides a more nuanced evaluation of treatment efficacy beyond the binary survival endpoint. Indeed, the calculation of the Bliss Index yielded a score of 5.8, indicating that the combined regimen of DY-III-281 and CV3-13-GASDALIE provided an additive benefit. The t-SNE plots corroborated this observation. Overall, our results demonstrate the potential of combining the antiviral activities of DY-III-281 with other prophylactic interventions to enhance *in vivo* efficacy.

## DISCUSSION

Small molecules have been used for years to fight viral infections. However, until now, no SARS-CoV-2 small-molecule drugs that directly target the SARS-CoV-2 Spike glycoprotein have been approved for therapeutic or prophylactic use in humans. VE607 is one small-molecule inhibitor that was originally developed to specifically target SARS-CoV-1 (40, 41). We previously repurposed VE607 and reported its capacity to inhibit SARS-CoV-2 entry (42). VE607 inhibits the infection of SARS-CoV-2 original Wuhan strain and Omicron sublineages by stabilizing the “up” conformation of the RBD and preventing viral entry (42). In this study, we aimed at improving potency and breadth of VE607 and developed a series of (R,R)-VE607 derivatives that were next tested for neutralizing efficacy against representative SARS-CoV-2 variants. Three analogs, DY-III-281, DY-III-287, and DY-IV-048, showed similar or superior capacity to inhibit entry of pseudoviral particles carrying Spike glycoproteins from Omicron subvariants, including KP.3.1.1 and XEC, as well as authentic viruses. As seen previously for VE607, these analogs stabilized the Spike in the RBD “up” conformation as measured by smFRET (DY-IV-048, DY-III-281) and cryo-EM (DY-III-281).

SARS-CoV-2 is an enveloped, positive-sense single-stranded RNA virus that enters host cells through a two-step process: initial attachment to the angiotensin-converting enzyme 2 (ACE2) receptor, followed by membrane fusion. The interaction between the RBD of the viral Spike glycoprotein and ACE2 triggers significant conformational rearrangements in the Spike, ultimately exposing the fusion peptide necessary for membrane fusion. Our smFRET and single-particle cryo-EM studies demonstrate that VE607 analogs stabilize the Spike in a conformation with one RBD in the “up” position. While our cryo-EM data suggest that the interaction between VE607 analogs and the Spike is likely transient, we observe a clear increase in the proportion of Spike trimers adopting the one RBD-up conformation. A limitation of our study is that we were unable to localize the compound within the Spike density, likely due to both the transient nature of the interaction and the reduced resolution in the RBD and NTD regions of the cryo-EM maps. Despite this transient binding, we postulate that VE607 analogs act by stabilizing the SARS-CoV-2 Spike in a conformation that interferes with the large-scale structural rearrangements required for viral entry.

K18-hACE2 transgenic mice are a highly susceptible model to test prophylactic or therapeutic interventions targeted against SARS-CoV-2 (29, 50). We have previously used this model to demonstrate that neutralizing antibodies required both neutralization and Fc-effector functions for effective SARS-CoV-2 clearance *in vivo* (20). We also observed that virus neutralization and Fc-effector functions could be separated, with each provided by a different antibody. When combined, these antibodies completely protected SARS-CoV-2-challenged mice even though neither was protective on their own (14). Here, we investigated the possibility of combining DY-III-281, a virus entry inhibitor, with CV3-13, a non-neutralizing antibody with enhanced Fc-effector functions. When combined, neither the entry inhibition by DY-III-281 nor the antibody binding of CV3-13 was altered (Fig. S1). Nevertheless, our multiparametric *in vivo* analyses indicated an additive benefit for DY-III-281 when combined with CV3-13 GASDALIE, supporting the potential of combination strategies to enhance the *in vivo* efficacy of next-generation DY-III-281 analogs.

Current SARS-CoV-2 Omicron subvariants harbor more than 60 mutations in their Spike compared to the original strain (51, 52). These viruses remain a challenge to protect against re-infection (53–56). Several clinically approved mAbs against SARS-CoV-2 have now become obsolete due to resistant mutations in Spike (Imdevimab, Casirivimab, Evusheld [57, 58]). Therefore, multiple tools of intervention are needed to fight the evolving SARS-CoV-2. With broad neutralizing capacity, VE607 analogs represent an additional tool of intervention against emerging SARS-CoV-2 VOCs.

## MATERIALS AND METHODS

Materials and methods have been previously reported (16, 23, 42, 43, 59) and are summarized below.

### Viruses

Authentic SARS-CoV-2 and XBB.1 were isolated, sequenced, and amplified from clinical samples obtained from infected patients by the Laboratoire de Santé Publique du Québec (LSPQ) and were previously described (60). The virus was sequenced by MinION technology (Oxford Nanopore Technologies, Oxford, UK). All work with the infectious SARS-CoV-2 authentic virus was performed in Biosafety Level 3 (BSL3) facilities at CRCHUM using appropriate positive-pressure air respirators and personal protective equipment. For *in vivo* experiments, we utilized SARS-CoV-2-WA1 expressing nanoluciferase (SARS-CoV-2_WA1-nLuc_), which was obtained from Craig B Wilen, Yale University, and generously provided by K. Plante and Pei-Yong Shi, World Reference Center for Emerging Viruses and Arboviruses, University of Texas Medical Branch) (61, 62). We propagated the virus by infecting Vero-E6-TMPRSS2 at a multiplicity of infection of 0.1. The culture supernatants were harvested after 72 h when cytopathic effects were clearly visible. To generate viral stocks, the supernatant was first cleared of cell debris by centrifugation and passed through a 0.45 micron filter. The viruses were then concentrated by mixing three parts of filtered supernatant with one part of cold (4°C) 4× PEG-it Virus Precipitation Solution (System Biosciences) and incubating the mixture overnight at 4°C. The precipitated virus was harvested by centrifugation at 1,500 × *g* for 60 min at 4°C and the resulting pellet resuspended in phosphate-buffered saline (PBS). Final stocks were aliquoted for storage at −80°C and estimation of virus titers by plaque assay. All work involving infectious SARS-CoV-2 was conducted within BSL3 and A-BSL3 facilities at Yale University School of Medicine following protocols approved by the IBSC and using appropriate protective equipment, including positive pressure air respirators.

### Plaque-forming assay

To determine the concentration of viral stocks, a standard plaque assay was performed. The procedure began by seeding Vero-E6 cells into 12-well plates at a density of 4 × 10^5^ cells per well and allowing them to adhere for 24 hours. Following this incubation, the cell monolayers were infected with serial dilutions of the virus. An overlay consisting of 1 mL of 0.6% Avicel (RC-581 FMC BioPolymer) in pre-warmed complete RPMI medium (Thermo Fisher Scientific) was then added to each well. After 48 hours, the cells were fixed for 15 minutes with 10% paraformaldehyde. To visualize the plaques, a staining solution of 0.2% crystal violet in 20% ethanol (both from Sigma Aldrich) was applied for 1 hour. Finally, the plates were rinsed gently with water to reveal the plaques for quantification.

### Chemical synthesis: general information

All solvents were reagent or high-performance liquid chromatography (HPLC) grade. Anhydrous CH2Cl2 and tetrahydrofuran were obtained from the Pure SolveTM PS-400 system under argon atmosphere. All reagents were purchased from commercially available sources and used as received. Reactions were magnetically stirred under a nitrogen or argon atmosphere, unless otherwise noted, and reactions were monitored by thin-layer chromatography performed on pre-coated silica gel 60 F-254 plates (40–55 micron, 230–400 mesh) and visualized by UV light. Yields refer to chromatographically and spectroscopically pure compounds. Optical rotations were measured on a JASCO P-2000 polarimeter. Proton (1H) and carbon (13C) nuclear magnetic resonance (NMR) spectra were recorded on a Bruker Avance III 500-MHz spectrometer or a Bruker NEO600 600-MHz spectrometer. Chemical shifts (δ) are reported in parts per million (ppm) relative to chloroform (δ 7.26), or methanol (δ 3.31) for 1H NMR, and chloroform (δ 77.2) or methanol (δ 49.15) for 13C NMR. High-resolution mass spectra (HRMS) were recorded at the University of Pennsylvania Mass Spectroscopy Service Center on either a VG Micromass 70/70H or VG ZAB-E spectrometer. Analytical HPLC was performed with a Waters HPLC-MS system consisting of a 515 pump and Sunfire C18 reverse phase column (20 µL injection volume, 5 µm packing material, and 4.5 × 50 mm column dimensions) with detection accomplished by a Micromass ZQ mass spectrometer and 2996 PDA detector. Preparative-scale HPLC was carried out on a Waters AutoPurification system (Milford, MA) equipped with a 3100 mass detector, a 2767 sample manager, and a 2489 UV/visible detector. Purification was done on a 19 × 100 mm SunFire Prep C18 OBD 5 µm column using a binary solvent gradient with mobile phase A (0.1% formic acid in water) and B (0.1% formic acid in acetonitrile). HPLC-grade water and acetonitrile, as well as Optima liquid chromatography-mass spectrometry (LC/MS)-grade formic acid, were purchased from Fisher Scientific and used without further purification. Fraction collection was triggered using the mass detector. X-ray intensity data were collected on a Rigaku XtaLAB Synergy-S diffractometer equipped with an HPC area detector (Dectris Pilatus3 R 200K) and employing confocal multilayer optic-monochromated Mo-Kα radiation (λ = 0.71073 Å) at a temperature of 100K. Preliminary indexing was performed from a series of thirty 0.5° rotation frames with exposures of 15 seconds. A total of 1,212 frames (nine runs) were collected employing ω scans with a crystal to detector distance of 34.0 mm, rotation widths of 0.5°, and exposures of 50 seconds. The purity of new compounds was judged to be >95% pure by NMR and LC-MS analysis, unless otherwise noted. Chemical synthesis is detailed in the supplemental material.

### X-ray crystallography

X-ray intensity data were collected on a Rigaku XtaLAB Synergy-S diffractometer equipped with an HPC area detector (Dectris Pilatus3 R 200K) and employing confocal multilayer optic-monochromated Mo-Kα radiation (λ = 0.71073 Å) at a temperature of 100K. Preliminary indexing was performed from a series of thirty 0.5° rotation frames with exposures of 15 seconds. A total of 1,212 frames (nine runs) were collected employing ω scans with a crystal to detector distance of 34.0 mm, rotation widths of 0.5°, and exposures of 50 seconds

### Cell viability test

To measure the cytotoxicity of VE607 and the derived compounds on 293T-ACE2 or Vero-E6 cells, a cell viability assay using CellTiter-Glo One Solution Assay (Promega) was performed. Briefly, 293T-ACE2 or Vero-E6 cells were seeded at a density of 10^4^ cells/well in 96-well luminometer-compatible tissue culture plates (Perkin Elmer). After 24 h, indicated concentrations of (R,R)-VE607, DY-III-281, DY-III-287, or DY-IV-048 up to concentrations of 100 µM were added to the cells, followed by incubation for 48 h at 37°C, and the same volume of its vehicle, DMSO, was added as a control. Then a volume of CellTiter-Glo One Solution buffer equal to the volume of cell culture medium present in each well was added, followed by 2 minutes mixing on a shaker and 10 minutes incubation at room temperature. An LB941 TriStar luminometer (Berthold Technologies) was used to measure the luciferase activity of each well.

### Neutralization assay using pseudoviral particles

Target cells were infected with single-round luciferase-expressing lentiviral particles as described previously (43). Briefly, 293T cells were transfected by the calcium phosphate method with the lentiviral vector pNL4.3 R-E- Luc (NIH AIDS Reagent Program) and a plasmid encoding for SARS-CoV-2 Spike glycoprotein (S) at a ratio of 5:4. Two days post-transfection, cell supernatants were harvested and stored at −80°C until use. Pseudoviral particles carrying VSV-G, SARS-CoV-1 S, or S from SARS-CoV-2 variants (SARS-CoV-2 D614G, B.1.1.7, BA.4/5, XBB.1.5, BA.2.86, EG.5.1, HK.3, JN.1, KP.3.1.1, and XEC) were produced, as previously reported (45). 293T-ACE2 target cells were seeded at a density of 1 × 10^4^ cells/well in 96-well luminometer-compatible tissue culture plates (Perkin Elmer) 24 h before infection. Pseudoviral particles in a final volume of 100 µL were incubated with the indicated concentrations of small molecules (R,R-VE607 or its derived compounds) up to concentrations of 100 µM for 1 h at 37°C and were then added to the target cells, followed by incubation for 48 h at 37°C. Cells were lysed by the addition of 30 µL of passive lysis buffer (Promega) followed by one freeze-thaw cycle. An LB941 TriStar luminometer (Berthold Technologies) was used to measure the luciferase activity of each well after the addition of 100 µL of luciferin buffer (15 mM MgSO4, 15 mM KPO4 [pH 7.8], 1 mM ATP, and 1 mM dithiothreitol) and 50 µL of 1 mM d-luciferin potassium salt (Prolume). The neutralization half-maximal inhibitory concentration (IC_50_) represents the concentration to inhibit 50% of the virus infection of 293T-ACE2 cells.

### Microneutralization with authentic viruses

One day prior to infection, 2 × 10^4^ Vero-E6 cells were seeded per well in the 96-well flat-bottom plate and incubated overnight to permit cell adherence. Compounds dilutions ranged from 0, 0.316, 1, 3.16, 10, 31.6, and 100 µM were performed in a separate 96-well culture plate using Dulbecco’s modified Eagle medium (DMEM) supplemented with penicillin (100 U/mL), streptomycin (100 µg/mL), HEPES, 0.12% sodium bicarbonate, 2% fetal bovine serum (FBS), and 0.24% BSA. 10^4^ TCID50/mL of SARS-CoV-2 virus was prepared in DMEM +2% FBS and combined with an equivalent volume of diluted compounds for 1 hour. After this incubation, all media was removed from the 96-well plate seeded with Vero-E6 cells, and a virus-compound mixture was added to each respective well at a volume corresponding to 600 TCID50 per well and incubated for one hour further at 37°C. Both virus-only and media-only (MEM +2% FBS) conditions were included in this assay. All virus-compound supernatant was removed from wells without disrupting the Vero-E6 monolayer. Each diluted compound (100 µL) was added to its respective Vero-E6 seeded well in addition to an equivalent volume of MEM +2% FBS and was then incubated for 48–72 hours. The media was then discarded and replaced with 10% formaldehyde for 24 hours to cross-link the Vero-E6 monolayer. Formaldehyde was removed from wells and subsequently washed with PBS. Cell monolayers were permeabilized for 15 minutes at room temperature with PBS + 0.1% Triton X-100, washed with PBS, and then incubated for 1 hour at room temperature with PBS + 3% non-fat milk. An anti-mouse SARS-CoV-2 nucleocapsid protein (Clone 1C7, Bioss Antibodies) primary antibody solution was prepared at 1 µg/mL in PBS + 1% non-fat milk and added to all wells for one hour at room temperature. Following extensive washing (3×) with PBS, an anti-mouse IgG HRP secondary antibody solution was formulated in PBS + 1% non-fat milk. One hour post-room temperature incubation, wells were washed with 3 × PBS, substrate (ECL) was added, and an LB941 TriStar luminometer (Berthold Technologies) was used to measure the signal of each well.

### Preparation of proteins for smFRET assays

Expression, purification, and fluorescent labeling of SARS-CoV-2 (Wuhan strain) ectodomain SΔTM trimers (SΔTM spikes) for smFRET experiments have been reported previously (46). Briefly, SΔTM hetero-trimers were expressed by co-transfection of ExpiCHO-S cells (Thermo Scientific, Waltham, MA, USA) with both the untagged SΔTM and A4-peptide-tagged SΔTM (amino acid positions 161 and 345) plasmids at a 2:1 molar ratio. SΔTM hetero-trimers were purified by affinity chromatography using nickel-nitrilotriacetic acid (Ni-NTA) agarose beads (Invitrogen, Waltham, MA, USA) and size exclusion chromatography (SEC), before being labeled by overnight incubation at room temperature with coenzyme A (CoA)-conjugated LD550 and LD650 fluorophores (Lumidyne Technologies, New York, NY, USA) and Acyl carrier protein synthase (AcpS). SΔTM was purified away from unbound dye and AcpS by a second round of SEC. Aliquots were stored at −80°C until use. Expression and preparation of soluble human monomeric ACE2 (hACE2) for smFRET experiments have been described previously (46).

### smFRET imaging and data analysis

Fluorescent-labeled SARS-CoV-2 SDTM spikes prepared as described above were immobilized on streptavidin-coated quartz microscope slides by way of Ni-NTA-biotin (Sigma-Aldrich, St. Louis, MO, USA) and imaged using wide‐field prism‐based total internal reflection fluorescence microscopy as described as well (46, 47, 63, 64). Imaging was performed in PBS containing 1 mM cyclooctatetraene (Sigma-Aldrich, St. Louis, MO, USA), 1 mM 4-nitrobenzyl alcohol (Sigma-Aldrich, St. Louis, MO, USA), 1 mM trolox (Sigma-Aldrich, St. Louis, MO, USA), 2 mM protocatechuic acid (Sigma-Aldrich, St. Louis, MO, USA), and 8 nM protocatechuate 3,4-deoxygenase (Sigma-Aldrich, St. Louis, MO, USA) to remove molecular oxygen and stabilize fluorescence. When indicated, 100 nM labeled SΔTM spikes were incubated with 600 nM of purified monomeric soluble human ACE2 (shACE2) as described (46), 312.5 nM (50 µg/mL) CV3-13 mAb, 50 µM DY-III-281, 50 µM DY-IV-048, or the indicated combination of them for 60 minutes before imaging. Concentrations of shACE2, CV3-13, DY-IV-048, DY-IV-048, and the indicated combinations of them were maintained during imaging. smFRET data were collected using Micromanager v2.0 (65) at 25 frames/s. All smFRET data were processed and analyzed using the SPARTAN software package (https://github.com/stjude-smc/SPARTAN) in Matlab (Mathworks, Natick, MA) (66). smFRET traces were identified according to criteria previously described (46, 47, 63). Traces meeting those criteria were then verified manually. Traces from each of three technical replicates were then compiled into FRET histograms, and the mean probability per histogram bin ±standard error was calculated. Traces were idealized to a three-state HMM (two nonzero-FRET states and a 0-FRET state) using the maximum point likelihood algorithm (67) implemented in SPARTAN. The three-state model was previously selected by comparing the Akaike information criterion across multiple different models with a range of state numbers and topologies as described (46). The idealizations were used to determine the occupancies (fraction of time until photobleaching) in each FRET state and construct Gaussian distributions, which were overlaid on the FRET histograms to visualize the results of the HMM analysis. The distributions in occupancies were used to construct violin plots in Matlab, as well as calculate mean occupancy and standard errors, as displayed in Fig. 5. Statistical significance measures (*P*-values) of FRET state occupancies were determined by one-way ANOVA in Matlab (The MathWorks, Waltham, MA, USA). The analysis displays the full breadth of dynamic behavior across the total population of traces analyzed. The total number of traces analyzed was sufficient to ensure minimally 85% statistical power during comparison of occupancy data from unbound to ligand-bound SΔTM (46). *P*-values < 0.05 were considered to indicate statistical significance.

### Protein expression and purification for Cryo-EM

SARS-CoV-2 spike construct HPM7 (48) was kindly provided by Dr. Andrew Ward (The Scripps Research Institute). The stabilized, uncleaved Spike trimer (HPM7) is based on the Wuhan strain with six additional proline (hexaproline or HP) mutations (F817P, A892P, A899P, A942P, K986P, and V987P) (68) and an engineered interprotomer disulfide (mut7 or M7) between residues 705 and 883 of the S2 subunit. SARS-CoV-2 spike HPM7 was transiently expressed in 293F GNTI- cells (Thermo Fisher Scientific) using transfection reagent FectoPRO (Polyplus 116-010). Briefly, diluted 50 µg HPM7 plasmid and 75 µL FectoPRO were mixed and incubated at room temperature for 10 min. Then the transfection mixture was added to 90 mL of 293F GNTI^−^ cells with cell viability of 95% and at a density of ~1 million cells/mL. 4 days after the transfection, supernatant was harvested and filtered with a 0.22 µm membrane. HPM7 protein was initially purified using Strep-Tactin XT resin (IBA Lifesciences) according to the manufacturer’s instructions. Then it was purified further by SEC using a Superdex 200 10/300 Gl (GE Healthcare) column. Protein purity was checked by SDS-PAGE, and protein concentration was determined using a NanoDrop by measuring the absorbance at 280 nm.

### Cryo-EM sample preparation and data collection

A stock solution of the small-molecule DY-III-281 was prepared in 100% DMSO to a final concentration of 100 mM. For complex preparation, DY-III-281 was serially diluted into PBS and mixed with 1.5 mg/mL of SEC-purified SARS-CoV-2 spike in a 10-fold excess to a final concentration of 500 µM. The mixture was incubated at 4 degrees overnight before cryo-EM grids preparation. Grids (Cu R1.2/1.3, 400 mesh, Quantifoil) were glow-discharged for 60 s at 15 mA. 3 µL of the sample was applied onto the grid, the grid was blotted for 5 s, and then it was plunge-frozen into liquid ethane using a Leica EM GP2 plunge freezer with its chamber set to 4°C and 95% humidity. As a control, an apo SARS-CoV-2 spike sample was prepared by incubation with an equivalent concentration and volume of DMSO without the compound. The cryo-EM grid for the apo sample was prepared using the same parameters. Cryo-EM data were acquired on a FEI Titan Krios electron (G1) microscope operating at 300 keV equipped with Gatan Bioquantum Image filter-K3 direct electron detector (Gatan Inc.) with 20 eV energy slit. 50-frame dose-fractionated movies in super resolution mode were collected at a nominal magnification of 105,000 corresponding to a calibrated physical pixel size of 0.832  Å/px (0.416 Å/px super resolution), with a total exposure dose of 54.2 e^−^/ Å2 at a dose rate of 15 e^-^/px/s and a defocus range of −0.5 to −2.7 µm. Automated data acquisition was done in SerialEM version 4.0.27 (69). More details can be found in Table S1.

### Cryo-EM data processing

Cryo-EM data sets of DMSO and DY-III-281-treated samples were processed using CryoSPARC v4.6.0 (70). Drift correction of raw micrographs was performed using Patch Motion Correction, and the contrast transfer function (CTF) was estimated by Patch CTF Estimation. Particle picking was carried out using the template picker, and two rounds of 2D classification were applied to eliminate junk particles. High-quality 2D class averages were used for *ab initio* reconstruction with three classes followed by heterogeneous refinement. To distinguish the different states of SARS-CoV-2 spike protein, particle alignment was constrained during subsequent 3D classification and 3D refinement steps. The initial round of 3D classification was conducted using three classes with a target resolution of 6 Å. Particles contributing to structurally distinct conformations were analyzed using UCSF Chimera (71) and grouped based on conformation. Each subset, representing open, closed, or intermediate conformations, was processed independently through iterative rounds of 3D classification until well-segregated classes were obtained. 3D classes of particles with the highest quality and resolution were selected and subjected to 3D refinement. A similar workflow, incorporating reference-based iterative 3D classification, was applied for the DMSO-treated spike data set. Final maps were generated using Non-uniform Refinement, and C1 symmetry was applied throughout all processing steps. The overall resolution of each map was assessed using the gold-standard Fourier shell correlation 0.143 criterion. Final density maps used for model building were derived from the non-uniform refinement results.

### Atomic model building and refinement

Final density maps obtained from non-uniform refinement were used to build atomic models of the open and closed conformational states of the SARS-CoV-2 spike protein. An initial fit to the open and closed state of Cryo-EM reconstruction was done using the cryo-EM structure of SARS-CoV-2-6P-Mut7 S protein complexed with CC6.33 IgG as a starting model (PDB ID 7RU3). To generate initial models, the CC6.33 IgG component was removed from the complex, retaining only the spike protein. Then only SARS-CoV-2-6P-Mut7 S protein was fitted into the corresponding cryo-EM maps using UCSF ChimeraX (72). To get the initial model of the closed state, the up-positioned RBD in the 7RU3 structure was truncated and reoriented to match the density corresponding to the down conformation, then merged back with the rest of the spike trimer to complete the closed-state model for refinement.

While the open and closed conformations maps obtained from DMSO-treated data sets were similar to those in the DY-III-281-treated data sets, the models derived from the DY-III-281 reconstructions did not dock well into the DMSO-treated cryo-EM maps. Therefore, for the DMSO-treated samples, initial models of the open and closed states were generated using the cryo-EM structure of the SARS-CoV-2 spike protein (PDB ID: 7N0G). After generating the initial models, these structures were then manually fitted into the reconstruction using COOT (73) and then refined with real-space refinement in PHENIX (74). Several rounds of refinement and model building were done to finalize the model for each of the conformational states. Data collection and refinement statistics are located in Table S1.

### Mouse experiments

All experiments were approved by the Institutional Animal Care and Use Committees and the Institutional Biosafety Committee (IBSC) of Yale University. All the animals were housed under specific pathogen-free conditions in the facilities provided and supported by Yale Animal Resources Center (YARC). hACE2 transgenic B6 mice (heterozygous) were obtained from Jackson Laboratory. 6- to 8-week-old male and female mice were used for all the experiments. The heterozygous mice were crossed and genotyped to select heterozygous mice for experiments using the primer sets recommended by Jackson Laboratory.

### Pharmacokinetics of DY-III-281

DY-III-281 or vehicle (DMSO) was administered intraperitoneally (i.p.) to male and female K18-hACE2 mice aged 6–8 weeks at doses of 50 mg/kg, 25 mg/kg, and 12.5 mg/kg, and the concentration in blood was measured 30 min, 2, 8, and 24 h after administration. The mice were sacrificed at 24 h, and the indicated organs or washes (lung, brain, liver, spleen, kidney, nasal wash, or BALF) were isolated for mass spectrometry. Tissue was weighed, resuspended in serum-free RPMI, and homogenized using 1.5 mm Zirconium beads with BeadBug 6 homogenizer (Benchmark Scientific, TEquipment Inc.). To determine drug concentrations, the homogenized tissue was briefly centrifuged at 13,000 rpm at 4°C, and the clarified supernatant containing free drug was analyzed by mass spectrometry, and data were extrapolated to reflect the amount of drug per organ based on weight.

### Mass spectrometry

DY-III-281 mouse plasma and tissue levels were determined by ultra-HPLC (UHPLC) HRMS. Briefly, DY-III-281 was extracted from mouse plasma and tissue homogenate samples using protein precipitation. Five hundred microliter of internal standard (IS) solution (2.0 ng/mL of VE607 in methanol) was added to an aliquot of 10 µL of sample. The sample was vortexed for approximately 5 s and let stand for a period of 10 min, then centrifuged at 13,000 × *g* for 10 min. The supernatant was transferred to 13 × 100 mm borosilicate tubes and evaporated to dryness at 50°C under a gentle stream of nitrogen. The dried extract was re-suspended with 200 µL of 10:90 methanol: 10 mM ammonium formate pH 3 solution and transferred to an injection vial for analysis.

The analysis was performed using a Thermo Scientific Q-Exactive Plus Orbitrap Mass Spectrometer interfaced with the Thermo Scientific UltiMate 3000 XRS UHPLC system using a pneumatic-assisted heated electrospray ion source. MS detection was performed in positive ion mode operating in scan mode at high resolution and accurate mass (HR/AM). Nitrogen was used for the sheath and auxiliary gases and was set at 35 and 15 arbitrary units. The HESI electrode was set to 3,500 V. The capillary temperature was set at 350°C, and the vaporizer temperature was set at 400°C. The scan range was set to m/z 200–600. Data were acquired at a resolving power of 70,000 (FWHM), resulting in a scanning rate of <0.75 scans/s when using an automatic gain control target of 3.0 × 10^6^ and a maximum ion injection time of 200 ms. Post-acquisition high-resolution extracted ion chromatograms were generated using exact masses of targeted compounds ± 5 ppm.

Chromatographic separation was achieved using gradient elution with a Phenomenex Phenyl-Hexyl analytical column (150 × 2.0 mm I.D., 5 µm) operating at 50°C. The initial mobile phase condition consisted of methanol and 10 mM ammonium formate, pH 3, at a ratio of 10:90, respectively, and this ratio was maintained for 1 min. From 1 to 4.5 min a linear gradient was applied up to a ratio of 90:10 and maintained for 0.6 min. At 5.1 min, the mobile phase composition was reverted to 10:90 and the column was allowed to equilibrate for 4.9 min for a total run time of 10 min. The flow rate was fixed at 0.35 mL/min, and DY-III-281 and IS were eluted at 3.7 and 3.8 min, respectively.

Data acquisition and processing were performed using Thermo Scientific Xcalibur 4.2.47. Calibration curves were generated using weighted (1/*x*) linear regression of DY-III-281/IS peak-area ratios, with an analytical range of 10–20,000 ng/mL. Sample concentrations were interpolated from the standard curve. The method demonstrated acceptable precision and accuracy per bioanalytical standards.

### Mouse studies

All animal studies were conducted using 6- to 8-week-old male and female heterozygous hACE2 transgenic B6 mice (K18-hACE2), which were originally sourced from The Jackson Laboratory (Bar Harbor, ME, USA). We established our own breeding colony and confirmed genotypes using the recommended primer sets. Experimental cohorts, composed of 4–8 animals, were assembled from randomly selected, sex- and age-matched littermates. The sample size for each group was justified by a priori power analysis using data from our previous work and pilot experiments (14, 20, 49). We made every effort to balance the number of male and female mice within experimental groups to mitigate sex as a confounding variable. No animals were excluded from the study post-procedure due to illness. All animals were maintained in the specific pathogen-free (SPF) barrier facility at the YARC with a 14:10 light-dark cycle. Animals infected with SARS-CoV-2 were housed in a dedicated BSL3 containment room, separate from the main breeding populations. All procedures involving infectious agents were performed under ABSL3 conditions, with personnel equipped with full protective gear, including pressurized air-purifying respirators, disposable gowns, and double gloves with shoe and sleeve covers. Decontamination and disposal of all animal-related materials followed the guidelines set by Yale University Environmental Health Services. The infection was initiated by intranasally delivering 1 × 10⁵ PFU of SARS-CoV-2_WA1-nLuc_ to anesthetized mice in a total volume of 25–30 µL. Anesthesia was administered with a precision Dräger vaporizer supplying 0.5%–5% isoflurane in oxygen. DY-III-281 was administered intraperitoneally (i.p. 25 mg/kg), 24 h prior to infection and daily until day 4 following infection. Isotype, CV3-13 LALA, or CV3-13 GASDALIE antibody was administered once (i.p., 12.5 mg/kg), 24 h before infection. Post-infection monitoring involved daily tracking of body weight relative to the initial measurement (100%). For mortality analysis beginning on day 6, we performed welfare checks every 8–12 hours. Humane endpoints were established, requiring euthanasia for any mouse that lost over 20% of its body weight or displayed signs of severe illness or lethargy. These euthanized animals were included as mortalities in Kaplan-Meier survival analyses.

### *Ex vivo* bioluminescence analysis

The imaging workflow was conducted entirely post-mortem. Each animal at the time of euthanasia (humane endpoints; see above) was anesthetized using isoflurane and received a systemic dose of 100 µL furimazine substrate (diluted 1:40 in sterile PBS) through a retro-orbital route. Following substrate dissemination, the animals were euthanized, and the carcass was first imaged in its entirety (whole body) using an IVIS Spectrum instrument housed within an XIC-3 biocontainment chamber (PerkinElmer, Inc.). Subsequently, the lungs and brain were carefully dissected. These explanted organs then had 200 µL of the substrate solution applied directly to their surface and were incubated for 1–2 minutes before undergoing a second round of imaging. All images were captured and analyzed with Living Image software (v4.7.3). Acquisition settings were automated, with a luminescent f/stop of 2, a photographic f/stop of 8, and medium binning. Photon flux was quantified as luminescent radiance (photons/sec/cm²/sr), and a uniform luminescent scale was applied across all images for accurate comparison. Signal thresholds were established to exclude background radiance measured from areas devoid of tissue.

### Measurement of viral burden in tissues

To assess viral loads, brain and lung tissues were harvested from both infected (at the time of death, post necropsy) and uninfected mice and weighed. The collected organs were homogenized in 1 mL of serum-free RPMI with penicillin-streptomycin using a BeadBug 6 homogenizer (Benchmark Scientific) with 1.5 mm Zirconium beads. Total RNA was isolated from tissue homogenates with the RNeasy Plus Mini kit (Qiagen, Cat # 74136) and subsequently reverse transcribed into cDNA using the iScript advanced cDNA kit (Bio-Rad, Cat #1725036). The abundance of the SARS-CoV-2 N gene was then quantified via SYBR Green Real-time PCR with the following primers: N-Forward (5′-ATGCTGCAATCGTGCTACAA-3′) and N-Reverse (5′-GACTGCCGCCTCTGCTC-3′). The specificity of the PCR product was confirmed for all reactions using melt-curve analysis.

### mRNA expression analyses of signature inflammatory cytokines and lung injury/repair genes

To analyze gene expression changes in response to infection, brain and lung tissues were sampled at the time of death post-necropsy. Approximately 20 mg of tissue from each organ was placed in 500 µL of RLT lysis buffer for RNA isolation with the RNeasy Plus Mini kit (Qiagen, Cat # 74136). The purified RNA was converted to cDNA with the iScript advanced cDNA kit (Bio-Rad, Cat #1725036). We then quantified the mRNA transcripts of key inflammatory cytokines and lung pathology markers using multiplex qPCR. This was performed with the iQ Multiplex Powermix (Bio-Rad, Cat # 1725848) and PrimePCR Probe Assays for murine *Gapdh*, *Il6*, *Ccl2*, *Cxcl10*, *Ifnɣ*, *Il1b*, *Krt8 (injury/repair marker*), *Krt5 (dysfunctional repair marker*), *Adamts4 (fibrosis marker*), and *Itga5 (damage-responsive fibroblasts marker*). Reactions were run on a CFX96 touch system (Bio-Rad) under the following thermal cycling conditions: an initial denaturation at 95°C for 2 min, followed by 40 cycles of 95°C for 10 s and 60°C for 45 s. A final melt-curve analysis verified the amplification of a single product per primer set. Transcript levels of target genes were normalized to *Gapdh* mRNA expression (ΔCt), and the fold change relative to uninfected controls was determined using the 2⁻^ΔΔCt^ method.

### Estimation of disease burden, Bliss Index Scores, and visualization

We calculated a “disease burden” score to provide a holistic measure of pathology, as previously described (49, 75). This composite score integrated measurements from brain and lung tissues, including viral loads (*N* mRNA copies), inflammatory cytokine, and pathology mRNA expression (*Ccl2*, *Ifng, Cxcl10, Krt8*), in the lung, and the extent of delayed death was also included, totaling seven parameters.

To calculate the score, each parameter was first normalized by setting the value from the control group (vehicle or isotype) to 100. A composite disease burden score was calculated by taking the mean of the seven normalized parameter values as shown in the equation below:


$$\text{Total disease burden}=\left[\%\;\text{reduction}\;\left(N{\text{ mRNA}}_{\text{lung}}+N{\text{ mRNA}}_{\text{brain}}+{Ccl2\;mRNA}_{\text{lung}}\;\;\;\;\;\;+{Cxcl10\;mRNA}_{\text{lung}}+{Ifng\;mRNA}_{\text{lung}}+{Krt8\;mRNA}_{\text{lung}}\right)\;\;\;\;\;\;+\%\text{ Mortality}+(100-\%\text{ Delay in death})\right]$$

To assess the nature of DY-III-281 and CV3-13 GASDALIE combination, we calculated Bliss index scores from the overall disease burden data as previously described (49, 75). The score was calculated using the following equation to quantify the nature of the interaction:


$$\begin{array}{rl} {Bliss\;index}_{\left(\text{DY-III-281}+\text{CV3-13GASDALIE}\right)} & =\%\;{Inhibition\;in\;disease\;burden}_{\left(\text{DY-III-281}+\text{CV3-13GASDALIE}\right)}\\ & \;-100[1-\left(1-\frac{\%\;{Inhibition\;in\;disease\;burden}_{\left(DY\text{-}III\text{-}281\right)}}{100}\right)\\ & \;\;\left(1-\frac{\%\;{Inhibition\;in\;disease\;burden}_{\left(CV3\text{-}13GASDALIE\right)}}{100}\right)] \end{array}$$

Based on the resulting Bliss index, interactions were classified as synergistic (score >10), additive (−10 ≤ score ≤ 10), or antagonistic (score < −10).

We also used the t-distributed stochastic neighbor embedding (t-SNE) algorithm to reduce the 7-dimensional data set to two dimensions for better visualization (Fig. 10D). Each parameter was z-score normalized across animals prior to dimensionality reduction. The analysis was performed in MATLAB R2024a using the tsne function with a perplexity of 8, a learning rate of 500, and random initialization.

### Quantification and statistical analysis

Statistics were analyzed using GraphPad Prism version 8.4 (GraphPad, San Diego, CA, USA). Every data set was tested for statistical normality, and this information was used to apply the appropriate (parametric or nonparametric) statistical test. Statistical details of experiments are indicated in the figure legends. *P* values < 0.05 were considered significant; significance values are indicated as *P < 0.05, **P < 0.01, ***P < 0.001, ****P < 0.0001.

## Data Availability

Data and reagents are available upon request. Cryo-EM maps have been deposited in the Electron Microscopy Data Bank (EMDB) under accession codes EMD-70454 (apo, closed), EMD-70455 (apo, open), EMD-70451 (DY-III-281, closed), and EMD-70453 (DY-III-281, open). The atomic coordinates have been deposited in the Protein Data Bank (PDB) under accession codes 9OG6 (apo, closed), 9OG7 (apo, open), 9OG4 (DY-III-281, closed), and 9OG5 (DY-III-281, open).

## SUPPLEMENTAL MATERIAL

Supplemental material jvi.01034-25-s0001.pdf Figures S1 to S5, Tables S1 and S2, and supplemental methods.

ASM does not own the copyrights to Supplemental Material that may be linked to, or accessed through, an article. The authors have granted ASM a non-exclusive, world-wide license to publish the Supplemental Material files. Please contact the corresponding author directly for reuse.
