# Supplementary material for: Optimization of VE607 to generate analogs with improved neutralization activities against SARS-CoV-2 variants
Source: J Virol. 2025 Oct 13;99(11):e01034-25. doi: 10.1128/jvi.01034-25 (PMC12645998; doi:10.1128/jvi.01034-25)
Supplement: Supplemental material — Figures S1 to S5, Tables S1 and S2, and supplemental methods. [file jvi.01034-25-s0001.pdf]

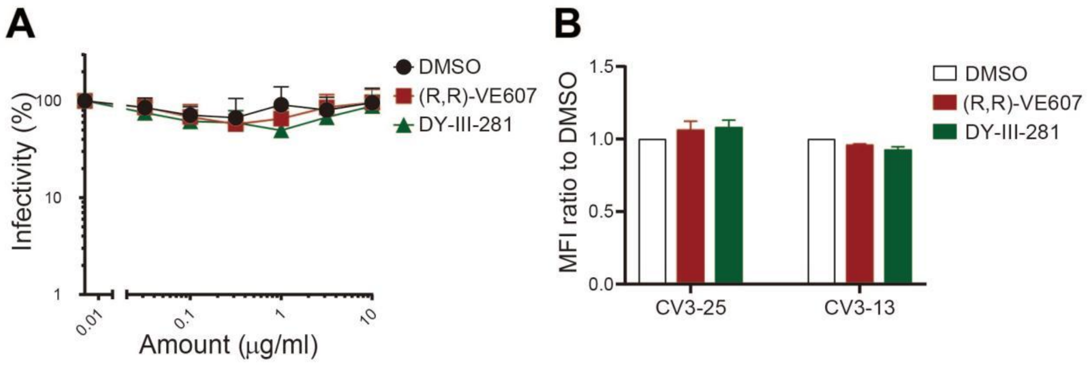


**Figure S1. DY-III-281 does not affect the capacity of CV3-13 to recognize the Spike.** (**A**) For neutralizing assay with pseudoviral particles bearing SARS-CoV-2 D614G Spike glycoprotein, virus was pre-incubated with 1μM of (R,R)-VE607, DY-III-281 or same volume of DMSO and then mixed with serial diluted CV3-13 as shown. Virus infectivity in the presence of CV3-13 was shown as the percentage to that without Ab. (**B**) For the binding with CV3-13, 293T cells transfected with Spike glycoprotein of SARS-CoV-2 D614G were pre-incubated with 50 μM of (R,R)-VE607, DY-III-281 or same volume of DMSO at 37℃, 5 μg/ml CV3-13 or CV3-25 was used for the staining in presence of indicated compounds. Relative binding was shown as the ratio between binding signals in presence of tested compounds and that in presence of DMSO. Data represents the average of at least two independent experiments +/- SEM.

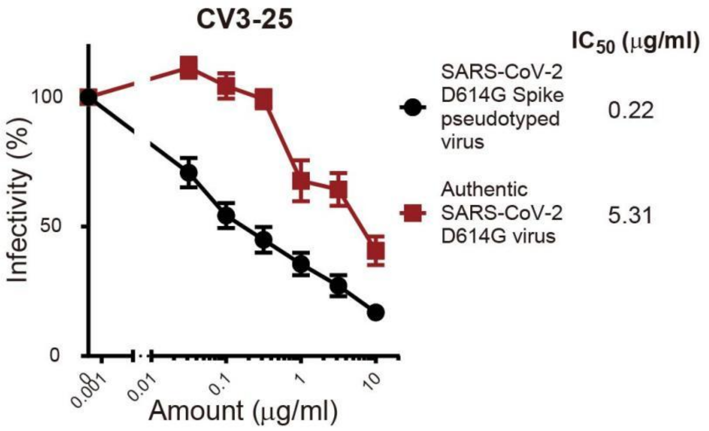


**Figure S2. Neutralization with monoclonal Ab CV3-25 against SARS-CoV-2 D614G Spike pseudotyped virus or authentic SARS-CoV-2 D614G virus.** For neutralizing assay with indicated viruses, serial diluted CV3-25 (start from 10 μg/ml) mAb was pre-incubated with SARS-CoV-2 D614G Spike pseudotyped virus (black solid cycle) or authentic SARS-CoV-2 D614G virus (red solid square) and the mixture was applied to 293T-hACE2 cells or Vero-E6 cells, respectively, for infection. Virus infectivity in the presence of Ab was shown as the percentage to that without Ab. IC50s were calculated and shown. Data represents the average of at least three independent experiments +/- SEM.

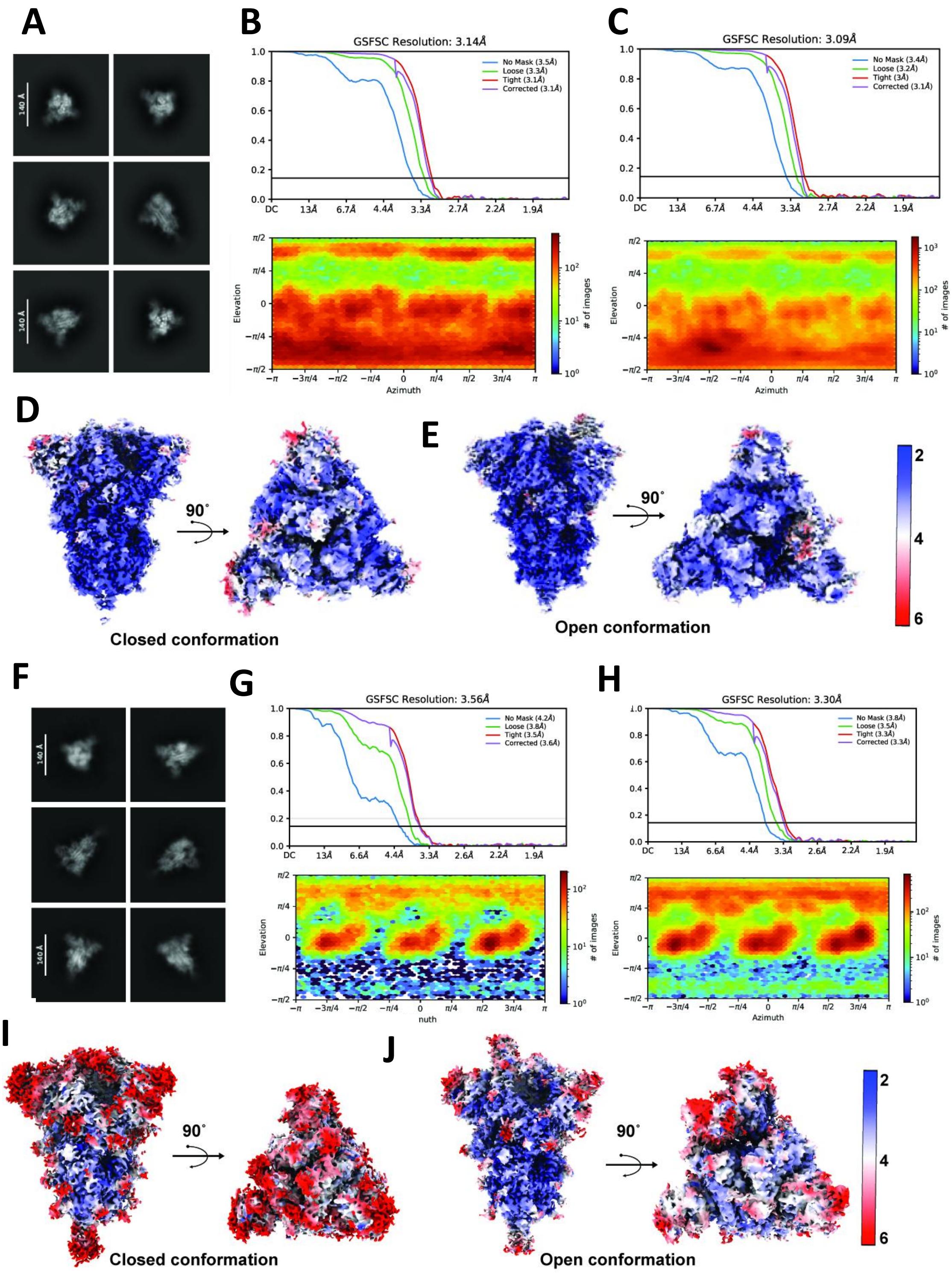


**Figure S3. Quality assessment of cryo-EM reconstructions.** SARS-CoV-2 apo and SARS-CoV-2 spike treated with DY-III-281 are represented by panels (**A - E**) and (**F - J**), respectively. (**A, F**) representative 2D classes averages selected for ab initio map reconstruction.– (**B, C, G, H**) Fourier Shell Correlation (FSC) curves between independently refined half-maps for the closed and open conformations of the spike protein (upper panel) and angular

distribution plot for all particles in the final reconstructions of closed and open conformations of spike (lower panel). Reported resolutions correspond to the gold-standard FSC threshold of 0.143. Color shading from blue to yellow correlates with the number of particles at a specific orientation as indicated in the key. (**D, E, I, J**) local resolution estimation of the final map of the closed (**D, I**) and (**E, J**) open conformational states of spike. Surface representations are colored according to local resolution, with the color scale indicated.

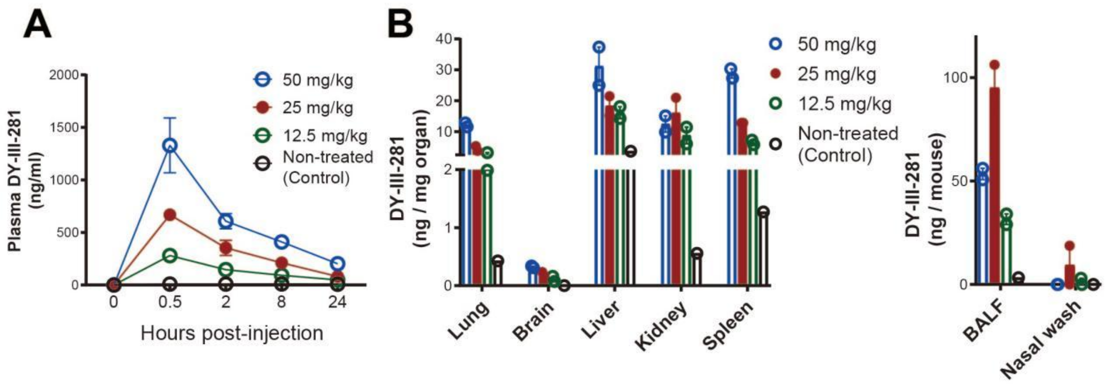


**Figure S4. Pharmacokinetics of DY-III-281 in K18-hACE2 Mice.** (**A**) DY-III-281 or vehicle (DMSO) was intraperitoneally injected into K18-hACE2 mice (n=2) at the indicated concentrations. Serum samples were collected at 0.5 hours, 2 hours, 8 hours and 24 hours post injection. The DY-III-281 serum concentrations (ng/ml) were determined by liquid chromatography-tandem mass spectrometry using mock-treated samples in parallel as baseline and DY-III-281 in DMSO as standard (see Materials and Methods). (**B**) DY-III-281 levels at 24 hour post-injection in indicated organs (ng/mg organ, left) and in BALF (800 µl) and nasal wash (200 ul) (ng/mouse, right) were quantified and reported; total drug per lavage (ng/mouse) was calculated as concentration × recovered volume.

| | Apo Spike | | DY-III-281 treated spike | |
|---|---|---|---|---|
| | Closed conformation | Open conformation | Closed conformation | Open conformation |
| EMDB | EMD-70454 | EMD-70455 | EMD-70451 | EMD-70453 |
| PDB | 9OG6 | 9OG7 | 9OG4 | 9OG5 |
| **Data Collection** | | | | |
| Microscope | FEI TITAN KRIOS | | | |
| Voltage (kV) | 300 | | | |
| Total exposure dose (e-/Å$^2$) | 54.2 | | | |
| Detector | Gatan K3 | | | |
| Pixel Size (Å) | 0.832 | | | |
| Defocus Range (μm) | 0.5-2.7 | | | |
| Magnification | 105,000 | | | |
| **Reconstruction** | | | | |
| Software | CryoSPARC | | | |
| Micrograph collected | 8808 | 8808 | 8826 | 8826 |
| Number of final particles | 340,310 | 625,582 | 53,445 | 214,848 |
| Symmetry | C1 | | | |
| Box size (pix) | 400 | | | |
| Resolution (Å) (FSC 0.143) | 3.14 | 3.09 | 3.56 | 3.30 |
| **Refinement (Phenix) & validation** | | | | |
| Protein residues | 3158 | 3130 | 3156 | 3021 |
| CC_mask | 0.87 | 0.86 | 0.84 | 0.86 |
| EMRinger Score | 2.58 | 2.49 | 1.54 | 1.82 |
| RMSD Bond lengths (Å) | 0.003 | 0.002 | 0.003 | 0.002 |
| RMSD Bond angles (˚) | 0.611 | 0.554 | 0.558 | 0.572 |
| Molprobity score | 2.36 | 2.22 | 2.55 | 2.31 |
| Clash score | 6.90 | 5.77 | 8.75 | 6.93 |
| Rotamer outliers (%) | 4.00 | 4.00 | 5.13 | 3.88 |
| **Ramachandran** | | | | |
| Favored (%) | 91.01 | 93.09 | 90.21 | 92.10 |
| Allowed (%) | 8.74 | 6.55 | 9.41 | 7.60 |
| Disallowed (%) | 0.26 | 0.36 | 0.39 | 0.30 |

**Table S1. Cryo-EM data collection and refinement statistics**

**Supplemental Experimental Procedures - General procedures**

**General procedure A: The synthesis of bis-epoxides.** Diol (**1,2**) (1.0 equiv) was dissolved in enantiopure (S)-epichlorohydrin (8.0 equiv) and heated to 100 °C. A solution of NaOH (2.0 equiv) in EtOH (0.75 mL/mmol) was then added dropwise. After stirring at 100 °C for three hours, the solution was cooled to r.t., diluted with acetone and filtered through a fritted funnel packed with celite. The filtrate was then concentrated in vacuo which was purified via flash column chromatography (5% to 10% EtOAc/hexanes) to yield the bis-epoxides (**7,8**) (90-97% yield).

**General procedure B: The synthesis of mono-epoxides.** The alcohol (**3 - 6**) (1.0 equiv) was dissolved in enantiopure (S)-epichlorohydrin (8.0 equiv) and heated to 100 °C. A solution of NaOH (2.0 equiv) in EtOH (0.75 mL/mmol) was then added dropwise. After stirring at 100 °C for three hours, the solution was cooled to r.t., diluted with acetone and filtered through a fritted funnel packed with celite. The filtrate was then concentrated in vacuo which was purified via flash column chromatography (5% to 10% EtOAc/hexanes) to yield the epoxides (**7 – 12**) (70-95% yield).

**General procedure C: Synthesis of one armed VE607 analogs.** Epoxide (1.0 equiv) was dissolved in the relevant amine (8 equiv) at 0 °C and stirred overnight at room temperature. Upon completion, solvent was removed *in vacuo* and the product was taken up in 1:1 MeCN/$H_2O$ and purified via Waters AutoPur with mass directed HPLC with a flow rate of 32 mL/min and the gradient program as follows: 0-0.5min 5% B, 1-9min linear from 5% to 20% B,

9-9.5min linear from 20% to 95% B, 9.5-11.5min 95% B and 11.5-12min 10% B. Fractions were collected based on a mass trigger and was lyophilized to yield the final product as clear or light yellow oils in 35-60% yield. Final compounds did not have optical rotation measurements as it was assumed that the stereochemistry of the secondary alcohol does not change.

**General procedure D: Synthesis of two armed VE607 analogs.** Epoxide (1.0 equiv) was dissolved in the relevant amine (16 equiv) at 0 °C and stirred overnight at room temperature. Upon completion, solvent was removed *in vacuo* and the product was taken up in 1:1 MeCN/$H_2O$ and purified via Waters AutoPur with mass directed HPLC with a flow rate of 32 mL/min and the gradient program as follows: 0-0.5min 5% B, 1-9min linear from 10% to 25% B, 9-9.5min linear from 10% to 25% B, 9.5-11.5min 25% B and 11.5-12min 10% B. Fractions were collected based on a mass trigger and was lyophilized to yield the final product as clear or light yellow oils in 35-60% yield. Final compounds did not have optical rotation measurements as it was assumed that the stereochemistry of the secondary alcohol does not change.

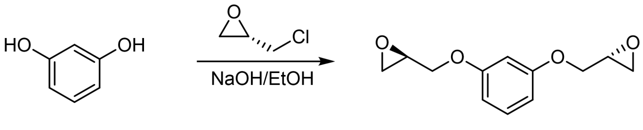


1 7

**1,3-bis(((R)-oxiran-2-yl)methoxy)benzene (7):** Was synthesized according to general procedure A with resorcinol (**1**) as the starting material. **$^1$H NMR** (500 MHz, CDCl3) δ 7.17 (t, J= 8.08 Hz, 1H), 6.55-6.51 (m, 3H), 4.20 (dd, J= 7.84, 3.17 Hz, 2H), 3.95 (dd, J= 5.66, 5.29 Hz, 2H), 3.36 – 3.33 (m, 2H), 2.90 (t, J= 4.77 Hz, 2H). 2.75 (dd, J= 2.63, 2.35 HZ, 2H); **$^{13}$C NM**R (125 MHz, CDCl3) δ 159.76, 130.06, 107.44, 101.94, 68.83, 50.13, 44.87; **HRMS** (ESI) m/z: [M+Na]+ calcd 245.0790, found 245.0791; **$[\alpha]_D^{23}$** +16.51 (c 0.75, MeOH).

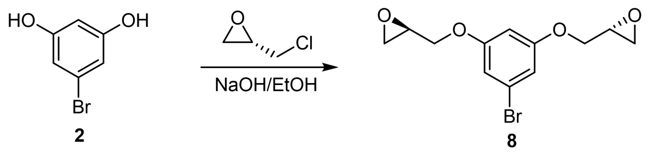


**(2R,2'R)-2,2'-(((5-bromo-1,3-phenylene)bis(oxy))bis(methylene))bis(oxirane) (8):** Was synthesized according to the general procedure A 5-bromobenzene-1,3-diol (**2**) as the starting material. **$^{1}$H NMR** (500 MHz, CDCl3) δ 6.72 – 6.68 (m, 2H), 6.45 (t, *J* = 2.3 Hz, 1H), 4.21 (dd, *J* = 11.0, 2.9 Hz, 2H), 3.88 (dd, *J* = 11.0, 5.7 Hz, 2H), 3.33 (h, *J* = 3.0 Hz, 2H), 2.91 (t, *J* = 4.6 Hz, 2H), 2.74 (dd, *J* = 5.0, 2.6 Hz, 2H). **$^{13}$C NM**R (125 MHz, CDCl3) δ 160.17, 123.10, 111.18, 101.16, 69.20, 50.04, 44.74; **HRMS** (ESI) m/z: [M+H]+ calcd 301.0070, found 301.0079; **$[\alpha]_D^{23}$** - 8.18 (c 1.0, $CH_2Cl_2$).

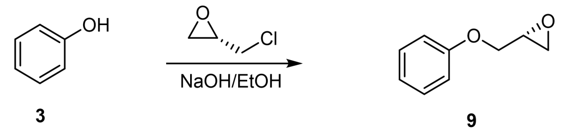


**(R)-2-(phenoxymethyl)oxirane (9):** Was synthesized according to the general procedure B with phenol (**3**) as the starting material. **$^{1}$H NMR** (500 MHz, MeOD) δ 7.31 – 7.23 (m, 2H), 6.97 – 6.90 (m, 3H), 4.31 (dd, *J* = 11.3, 2.6 Hz, 1H), 3.86 (dd, *J* = 11.3, 6.1 Hz, 1H), 2.91 – 2.85 (m, 1H), 2.75 (dd, *J* = 5.0, 2.7 Hz, 1H); **$^{13}$C NMR** (151 MHz, MeOD) δ 158.69, 129.11, 120.73, 114.25, 68.67, 50.00, 43.60; **HRMS** (ESI) m/z: [M+H]+ calcd 151.0759, found 151.0761; **$[\alpha]_D^{23}$** – 13.52(c 1.0, $CH_2Cl_2$).

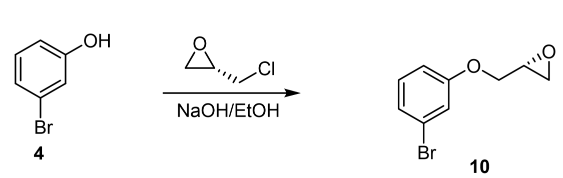

**(R)-2-((3-bromophenoxy)methyl)oxirane (10):** Was synthesized according to the general procedure B with 3-bromophenol (**4**) as the starting material. **$^1$H NMR** (500 MHz, $CDCl_3$) δ 7.16 – 7.06 (m, 3H), 6.86 (ddd, *J* = 8.0, 2.6, 1.2 Hz, 1H), 4.23 (dd, *J* = 11.0, 3.0 Hz, 1H), 3.92 (dd, *J* = 11.0, 5.8 Hz, 1H), 3.35 (ddt, *J* = 5.7, 4.1, 2.8 Hz, 1H), 2.94 – 2.87 (m, 1H), 2.76 (dd, *J* = 4.9, 2.6 Hz, 1H); **$^{13}$C NMR** (126 MHz, $CDCl_3$) δ 159.36, 130.77, 124.52, 122.97, 118.09, 113.80, 69.12, 50.10, 44.77. **HRMS** (ESI) m/z: [M+Na]+ calcd 322.98908, found 322.98894; **$[\alpha]_D^{23}$** – 8.8 (c 0.7, $CH_3OH$).

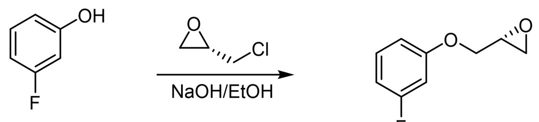


**5** **11**

**(R)-2-((3-fluorophenoxy)methyl)oxirane (11):** Was synthesized according to the general procedure B with 3-fluorophenol (**5**) as the starting material. **$^1$H NMR (500 MHz, $CDCl_3$)** δ 7.25 – 7.19 (m, 1H), 6.74 – 6.61 (m, 3H), 4.23 (dd, *J* = 11.0, 3.0 Hz, 1H), 3.93 (dd, *J* = 11.0, 5.8 Hz, 1H), 3.35 (ddt, *J* = 5.8, 4.2, 2.8 Hz, 1H), 2.92 (t, *J* = 4.5 Hz, 1H), 2.76 (dd, *J* = 4.9, 2.6 Hz, 1H); **$^{13}$C NMR (126 MHz, $CDCl_3$)** δ 163.71 (d, $J_{CF}$ = 244.73 Hz) , 159.97 (d, $J_{CF}$ = 12.31 Hz), 130.44 (d, $J_{CF}$ = 10.44 Hz), 110.48 (d, $J_{CF}$ = 3.1 Hz), 108.22 (d, $J_{CF}$ = 21.45 Hz), 102.60 (d, $J_{CF}$ = 12.23 Hz), 69.14, 50.11, 44.79; **HRMS** (ESI) m/z: [M+H]+ calcd 169.0659, found 168.0655; **$[\alpha]_D^{23}$** – 5.45 (c 0.63, $CH_3OH$).

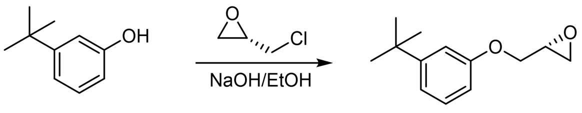


**6** **12**

**(R)-2-((3-(tert-butyl)phenoxy)methyl)oxirane (12):** Was synthesized according to the epoxide general procedure with 3-*tert*-butylphenol (**6**) as the starting material. **$^1$H NMR (500 MHz, $CDCl_3$)** δ 7.22 (d, *J* = 8.0 Hz, 1H), 7.04 –

6.96 (m, 2H), 6.76 – 6.70 (m, 1H), 4.21 (dd, *J* = 10.9, 3.3 Hz, 1H), 3.98 (dd, *J* = 10.9, 5.6 Hz, 1H), 3.37 (tdd, *J* = 5.9, 4.0, 2.8 Hz, 1H), 2.94 – 2.88 (m, 1H), 2.77 (dd, *J* = 5.0, 2.7 Hz, 1H), 1.31 (s, 7H); **$^{13}$C NMR (126 MHz, $CDCl_3$)** δ 158.45, 153.27, 129.14, 118.56, 112.91, 110.77, 68.83, 50.36, 45.00, 34.92, 31.45; **HRMS** (ESI) m/z: [M+H]+ calcd 207.1380, found 207.1380; **$[\alpha]_D^{23}$** – 7.60 (c 0.80, $CH_3OH$)

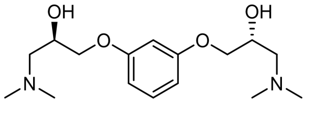

**DY-III-277**
**15**

**(2R,2'R)-3,3'-(1,3-phenylenebis(oxy))bis(1-(dimethylamino)propan-2-ol) (DY-III-277) (15):** Was synthesized according to General Procedure A with epoxide **7** and dimethylamine as the amine and isolated as a clear oil (2.15 mg) **$^1$H NMR (500 MHz, MeOD)** δ 7.19 – 7.12 (m, 1H), 6.54 (d, *J* = 7.7 Hz, 3H), 4.11 – 4.03 (m, 2H), 3.95 (dd, *J* = 9.8, 4.3 Hz, 2H), 3.89 (dd, *J* = 9.8, 5.9 Hz, 2H), 2.51 (qd, *J* = 12.9, 6.0 Hz, 4H), 2.32 (s, 12H); **$^{13}$C NMR (151 MHz, MeOD)** δ 160.18, 129.55, 106.74, 101.30, 70.59, 67.36, 61.92, 44.84; **HRMS** (ESI) m/z: [M+H]+ calcd for $C_{16}H_{29}N_2O_4$: 313.21218, found 313.21219.

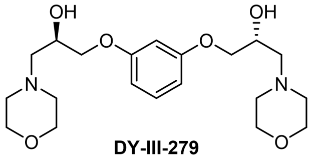

**DY-III-279**
**16**

**(2R,2'R)-3,3'-(1,3-phenylenebis(oxy))bis(1-morpholinopropan-2-ol) (DY-III-279) (16):** Was synthesized according to General Procedure A with epoxide **7** and morpholine as the amine and isolated as a clear oil (1.82 mg). **$^1$H NMR (500 MHz, MeOD)** δ 7.20 – 7.13 (m, 1H), 6.55 (d, *J* = 7.0 Hz, 3H), 4.13 (q, *J* = 5.6 Hz, 2H), 3.99 (dd, *J* = 9.8, 4.2 Hz, 2H), 3.92 (dd, *J* = 9.8, 5.7

Hz, 2H), 3.72 (t, *J* = 4.7 Hz, 8H), 2.67 – 2.54 (m, 12H); **$^{13}$C NMR (126 MHz, MeOD)** δ 160.10, 129.84, 106.97, 101.54, 70.25, 65.33, 65.08, 60.42, 53.29; **HRMS** (ESI) m/z: [M+H]+ calcd for $C_{20}H_{33}N_2O_6$: 397.23330, found 397.233325.

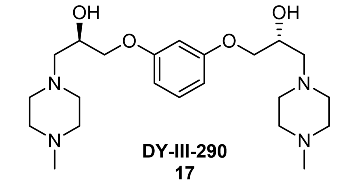


**DY-III-290**
**17**

**(2R,2'R)-3,3'-(1,3-phenylenebis(oxy))bis(1-(4-methylpiperazin-1-yl)propan-2-ol) (DY-III-290) (17):** Was synthesized according to general procedure A with epoxide **7** and *N*-methylpiperizine as the amine and isolated as a clear oil (1.1 mg). **$^{1}$H NMR (500 MHz, MeOD)** δ 7.20 – 7.13 (m, 1H), 6.55 (dt, *J* = 5.1, 1.7 Hz, 3H), 4.16 – 4.08 (m, 2H), 3.99 (dd, *J* = 9.8, 4.3 Hz, 2H), 3.93 (dd, *J* = 9.8, 5.6 Hz, 2H), 3.10 (s, 8H), 2.86 (s, 8H), 2.76 – 2.63 (m, 10H); **$^{13}$C NMR (126 MHz, MeOD)** δ 160.26, 129.79, 106.82, 101.56, 70.32, 66.94, 59.75, 53.38, 51.04, 42.74; **HRMS** (ESI) m/z: [M+H]+ calcd for $C_{22}H_{39}N_4O_4$: 423.29658, found 423.29689.

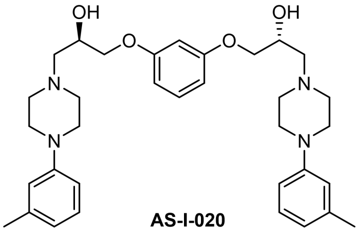


**AS-I-020**
**18**

**(2R,2'R)-3,3'-(1,3-phenylenebis(oxy))bis(1-(4-(m-tolyl)piperazin-1-yl)propan-2-ol) (AS-I-020) (18):** Was synthesized according to general procedure A with epoxide **7** and 1-(*m*-tolyl)piperazine as the amine and isolated as a clear oil (6.15 mg). **$^{1}$H NMR (500 MHz, MeOD)** δ 7.26 – 7.13 (m, 3H), 6.91 – 6.74 (m, 7H), 6.64 – 6.57 (m, 2H), 4.45 (dq, *J* = 9.2, 4.2 Hz, 1H),

4.03 (qd, *J* = 9.8, 5.0 Hz, 2H), 3.85 – 3.74 (m, 4H), 3.72 – 3.66 (m, 2H), 3.65 – 3.59 (m, 1H), 3.42 – 3.46 (m, 6H), 3.36 (m, 8H), 3.27 – 3.20 (m, 1H), 3.20 – 3.15 (m, 1H), 2.31 (d, *J* = 3.0 Hz, 6H); **$^{13}$C NMR (126 MHz, MeOD)** δ 161.86, 159.89, 150.48, 149.97, 138.98, 128.94, 128.90, 121.97, 117.96, 117.61, 117.45, 114.42, 114.02, 113.87, 107.19, 101.59, 69.89, 63.94, 58.88, 46.83, 43.56, 20.46; **HRMS** (ESI) m/z: [M+H]+ calcd for $C_{34}H_{47}N_4O_4$: 575.3598, found 575.3592.

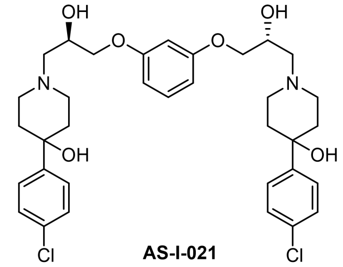


**AS-I-021**
**19**

**1,1'-((2R,2'R)-(1,3-phenylenebis(oxy))bis(2-hydroxypropane-3,1-diyl))bis(4-(4-chlorophenyl)piperidin-4-ol) (AS-I-021) (19):** Was synthesized according to general procedure B with epoxide **7** and 4-(4-chlorophenyl)piperidin-4-ol as the nucleophile and isolated as a clear oil (1.90 mg). **$^{1}$H NMR (500 MHz, MeOD)** δ 7.53 – 7.50 (m, 2H), 7.41 – 7.37 (m, 3H), 7.25 – 7.16 (m, 1H), 6.65 – 6.55 (m, 3H), 4.46 (dd, *J* = 10.1, 5.3 Hz, 1H), 4.17 – 3.92 (m, 4H), 3.88 – 3.65 (m, 1H), 3.69 – 3.51 (m, 4H), 3.51 – 3.35 (m, 6H), 2.47 – 2.28 (m, 4H), 2.05 – 1.90 (m, 4H); **$^{13}$C NMR (126 MHz, MeOD)** δ 160.44, 145.76, 133.06, 129.99, 128.28, 126.48, 107.20, 101.61, 70.50, 69.92, 68.94 67.84, 67.83, 63.92, 62.89, 59.41, 50.93, 45.44, 35.24; **HRMS** (ESI) m/z: [M+H]+ calcd for $C_{34}H_{43}Cl_2N_4O_6$: 645.24821, found 645.24927.

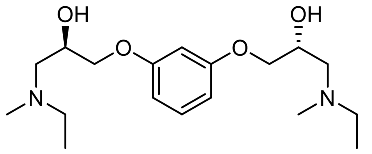

**AS-I-013**
**20**

**(2R,2'R)-3,3'-(1,3-phenylenebis(oxy))bis(1-(ethyl(methyl)amino)propan-2-ol) (AS-I-013) (20):** Was synthesized according to general procedure B with epoxide **7** and *N*-methylethanamine as the amine and isolated as a clear oil (4.52 mg). **$^{1}$H NMR (500 MHz, MeOD)** δ 7.22 (t, *J* = 8.2 Hz, 1H), 6.63 – 6.56 (m, 3H), 4.39 – 4.31 (m, 2H), 4.00 (tq, *J* = 9.6, 4.6 Hz, 4H), 3.41-3.27 (m, *J* = 6.7 Hz, 8H), 2.94 (d, *J* = 12.7 Hz, 6H), 1.37 (td, *J* = 7.3, 2.3 Hz, 6H); **$^{13}$C NMR (126 MHz, MeOD)** δ 159.88, 130.01, 107.09, 101.60, 69.82, 63.78, 52.98, 40.65, 38.54, 8.15; **HRMS** (ESI) m/z: [M+H]+ calcd for $C_{18}H_{33}N_2O_4$: 341.29356, found 341.29348.

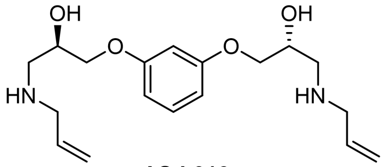

**AS-I-016**
**21**

**(2R,2'R)-3,3'-(1,3-phenylenebis(oxy))bis(1-(allylamino)propan-2-ol) (AS-I-016) (21):** Was synthesized according to general procedure A with epoxide **7** and allyl amine as the amine and isolated as a clear oil (2.26 mg). **$^{1}$H NMR (500 MHz, MeOD)** δ 7.20 (t, *J* = 8.1 Hz, 1H), 6.62 – 6.55 (m, 3H), 5.96 (dt, *J* = 16.9, 8.6 Hz, 2H), 5.58 – 5.49 (m, 4H), 4.24 (s, 2H), 4.05 – 3.94 (m, 4H), 3.71 (d, *J* = 6.8 Hz, 4H), 3.17 – 3.09 (m, 2H); **$^{13}$C NMR (151 MHz, MeOD)** δ 159.75, 129.82, 127.89, 123.01, 106.99, 101.44, 69.64, 65.29, 49.59, 49.07; **HRMS** (ESI) m/z: [M+H]+ calcd for $C_{18}H_{29}N_2O_4$: 337.21218, found 337.21225.

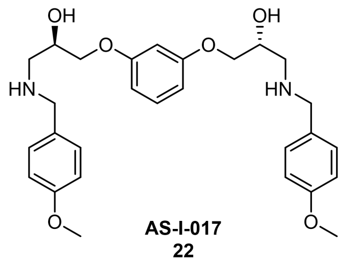

**AS-I-017**
**22**

**(2R,2'R)-3,3'-(1,3-phenylenebis(oxy))bis(1-((4-methoxybenzyl)amino)propan-2-ol) (AS-I-017) (22):** Was synthesized according to general procedure A with epoxide **7** and (4-methoxyphenyl)methanamine as the amine and isolated as a clear oil (3.06 mg). **$^{1}$H NMR (500 MHz, MeOD)** δ 7.41 (d, *J* = 8.5 Hz, 4H), 7.18 (t, *J* = 8.2 Hz, 1H), 6.99 (d, *J* = 8.5 Hz, 4H), 6.55 (dd, *J* = 8.2, 2.3 Hz, 2H), 6.51 (d, *J* = 2.4 Hz, 1H), 4.22 (d, *J* = 4.9 Hz, 2H), 4.17 (s, 4H), 4.02 – 3.90 (m, 4H), 3.82 (s, 6H), 3.21 (dd, *J* = 12.7, 3.1 Hz, 2H), 3.06 (dd, *J* = 12.7, 9.6 Hz, 2H); **$^{13}$C NMR (151 MHz, MeOD)** δ 160.66, 159.73, 131.11, 129.78, 114.13, 106.96, 101.40, 69.70, 65.44, 54.43, 50.59, 48.99; **HRMS** (ESI) m/z: [M+H]+ calcd for $C_{28}H_{37}N_2O_6$: 497.26469, found 497.26461.

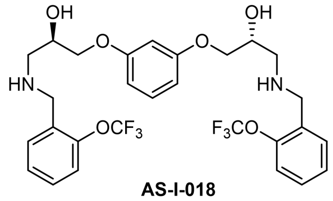

**AS-I-018**
**23**

**(2R,2'R)-3,3'-(1,3-phenylenebis(oxy))bis(1-((2-(trifluoromethoxy)benzyl)amino)propan-2-ol) (AS-I-018) (23):** Was synthesized according to general procedure A with epoxide **7** and (2-(trifluoromethoxy)phenyl)methanamine as the amine and isolated as a clear oil (3.39 mg).

**$^{1}$H NMR (500 MHz, MeOD)** δ 7.67 (d, *J* = 7.5 Hz, 2H), 7.62 (t, *J* = 7.9 Hz, 2H), 7.49 (t, *J* = 7.5 Hz, 4H), 7.24 – 7.15 (m, 1H), 6.62 – 6.51 (m, 3H), 4.47 – 4.36

(m, 4H), 4.33 – 4.25 (m, 2H), 4.07 – 3.92 (m, 4H), 3.78 - 3.64 (m, 2H), 3.21 (m, 2H); **$^{13}$C NMR (126 MHz, MeOD)** δ 159.84, 148.03, 131.97, 131.62, 129.94, 129.80, 127.61, 123.61, 120.39, 107.14, 101.53, 69.73, 65.21, 49.75, 45.07; **HRMS** (ESI) m/z: [M+H]+ calcd for $C_{28}H_{31}F_6N_2O_6$: 605.20842, found 605.20808.

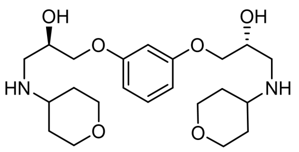


**AS-I-019**
**24**

**(2R,2'R)-3,3'-(1,3-phenylenebis(oxy))bis(1-((tetrahydro-2H-pyran-4-yl)amino)propan-2-ol) (AS-I-019) (24):** Was synthesized according to general procedure A with epoxide **7** and tetrahydro-2*H*-pyran-4-amine as the amine and isolated as a yellow oil (1.84 mg). **$^{1}$H NMR (500 MHz, MeOD)** δ 7.21 (t, *J* = 8.2 Hz, 1H), 6.63 – 6.55 (m, 3H), 4.28 – 4.19 (m, 2H), 4.08 – 3.95 (m, 8H), 3.49 – 3.37 (m, 6H), 3.41 – 3. 33 (m, 2H), 3.18 (dd, *J* = 12.7, 9.8 Hz, 2H), 2.07 - 2.02 (m, 4H), 1.78 – 1.63 (m, 4H); **$^{13}$C NMR (126 MHz, MeOD)** δ 159.88, 130.00, 107.04, 101.59, 69.74, 65.69, 65.61, 54.61, 29.31, 28.95; **HRMS** (ESI) m/z: [M+H]+ calcd for $C_{22}H_{37}N_2O_6$: 425.26508, found 425.26461.

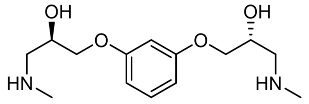


**AS-I-011**
**25**

**(2R,2'R)-3,3'-(1,3-phenylenebis(oxy))bis(1-(methylamino)propan-2-ol) (AS-I-011) (25):** Was synthesized according to general procedure A with epoxide **7** and methylamine as the amine and isolated as a clear oil (3.74 mg). **$^{1}$H NMR (500 MHz, MeOD)** δ 7.20 (t, *J* = 8.2 Hz, 1H), 6.62 – 6.55 (m, 3H), 4.23 (dtd, *J* = 9.7, 5.2, 3.2 Hz, 2H), 4.05 – 3.94 (m, 4H), 3.27 (dd, *J* = 12.7, 3.2

Hz, 2H), 3.15 (dd, *J* = 12.7, 9.5 Hz, 2H), 2.76 (s, 6H); **$^{13}$C NMR (126 MHz, MeOD)** δ 159.88, 129.95, 107.09, 101.56, 69.72, 65.27, 51.54, 32.62; **HRMS** (ESI) m/z: [M+H]+ calcd for $C_{14}H_{25}N_2O_4$: 285.1817, found 285.1814.

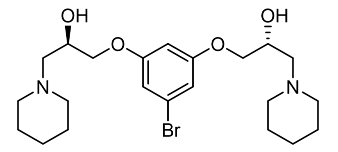


**DY-III-272**
**26**

**(2R,2'R)-3,3'-((5-bromo-1,3-phenylene)bis(oxy))bis(1-(piperidin-1-yl)propan-2-ol) (DY-III-272) (26):** Was synthesized according to General Procedure A with epoxide **8** and piperidine as the amine and isolated as a white powder (1.84 mg). **$^{1}$H NMR (500 MHz, MeOD)** δ 6.73 (d, *J* = 2.1 Hz, 2H), 6.53 (d, *J* = 2.3 Hz, 1H), 4.09 (d, *J* = 6.1 Hz, 2H), 3.97 (dd, *J* = 9.9, 4.0 Hz, 2H), 3.88 (dd, *J* = 9.9, 6.0 Hz, 2H), 2.53 - 2.45 (m, 12H), 1.63 (m, 8H), 1.48 (d, *J* = 6.6 Hz, 4H); **$^{13}$C NMR (151 MHz, MeOD)** δ 160.70, 122.43, 110.32, 100.46, 71.06, 66.66, 61.47, 54.80, 44.91, 25.26, 23.71, 23.49, 22.49; **HRMS** (ESI) m/z: [M+H]+ calcd for $C_{22}H_{36}BrN_2O_4$: 471.1853, found 471.1857.

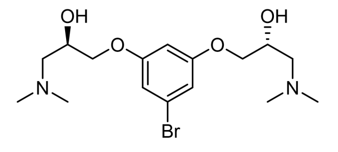


**DY-III-281**
**27**

**(2R,2'R)-3,3'-((5-bromo-1,3-phenylene)bis(oxy))bis(1-(dimethylamino)propan-2-ol) (DY-III-281) (27):** Was synthesized according to general procedure A with epoxide **8** and dimethylamine as the amine and isolated as a clear oil (1.92 mg). **$^{1}$H NMR (500 MHz, MeOD)** δ 6.80 (d, *J* = 2.2 Hz, 2H), 6.57 (t, *J* = 2.2 Hz, 1H), 4.30 – 4.22 (m, 2H), 4.04 – 3.94 (m, 4H), 3.07 (d, *J* = 6.6 Hz, 4H), 2.76 (s, 12H); **$^{13}$C NMR (151 MHz, MeOD)** δ 160.25,

122.66, 110.65, 100.59, 70.01, 63.78, 59.36, 29.25; **HRMS** (ESI) m/z: [M+H]+ calcd for $C_{16}H_{28}BrN_2O_4$: 391.1233, found 391.1232.

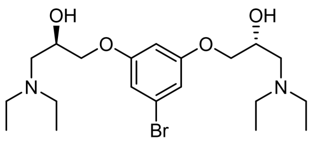


**DY-III-278**
**28**

**(2R,2'R)-3,3'-((5-bromo-1,3-phenylene)bis(oxy))bis(1-(diethylamino)propan-2-ol) (DY-III-278) (28):** Was synthesized according to general procedure A with epoxide **8** and diethylamine as the amine and isolated as a clear oil (3.85 mg). **$^1$H NMR (500 MHz, MeOD)** δ 6.79 (d, *J* = 2.1 Hz, 2H), 6.57 (d, *J* = 2.3 Hz, 1H), 4.31 – 4.25 (m, 2H), 4.00 (d, *J* = 5.0 Hz, 4H), 3.22 (m, 12H), 1.31 (t, *J* = 7.3 Hz, 12H); **$^{13}$C NMR (151 MHz, MeOD)** δ 160.33, 122.61, 110.62, 100.60, 70.18, 64.52, 54.19, 29.26, 8.03; **HRMS** (ESI) m/z: [M+H]+ calcd for $C_{20}H_{36}BrN_2O_4$: 447.18558, found 447.18530.

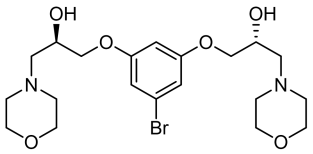


**DY-III-283**
**29**

**(2R,2'R)-3,3'-((5-bromo-1,3-phenylene)bis(oxy))bis(1-morpholinopropan-2-ol)**

**(DY-III-283) (29):** Was synthesized according to general procedure A with epoxide **6.63** and morpholine as the amine and isolated as a clear oil (0.9 mg). **$^1$H NMR (500 MHz, MeOD)** δ 6.74 (d, *J* = 2.2 Hz, 2H), 6.54 (t, *J* = 2.2 Hz, 1H), 4.12 (dq, *J* = 7.5, 5.2 Hz, 2H), 4.01 (dd, *J* = 9.9, 4.0 Hz, 2H), 3.92 (dd, *J* = 9.9, 5.8 Hz, 2H), 3.72 (t, *J* = 4.7 Hz, 8H), 2.65 – 2.53 (m, 12H); **$^{13}$C NMR (126 MHz, MeOD)** δ 160.68, 122.65, 110.56, 100.67, 70.71, 65.55, 65.43, 60.45, 53.48;

**HRMS** (ESI) m/z: [M+H]+ calcd for $C_{20}H_{32}BrN_2O_6$: 475.14383, found 475.14392.

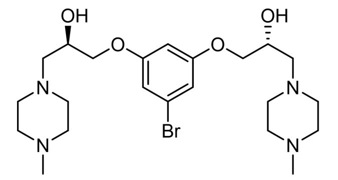

**DY-III-287**
**30**

**(2R,2'R)-3,3'-((5-bromo-1,3-phenylene)bis(oxy))bis(1-(4-methylpiperazin-1-yl)propan-2-ol) (DY-III-287) (30):** Was synthesized according to general procedure A with epoxide **8** and *N*-methylpiperizine as the amine and isolated as a clear oil (2.07 mg). **$^1$H NMR (600 MHz, MeOD)** δ 6.74 (d, *J* = 2.1 Hz, 2H), 6.53 (t, *J* = 2.2 Hz, 1H), 4.13 – 4.07 (m, 2H), 4.00 (dd, *J* = 9.8, 4.0 Hz, 2H), 3.93 (dd, *J* = 9.8, 5.7 Hz, 2H), 2.94 (s, 10H), 2.78 (s, 4H), 2.69 – 2.60 (m, 4H), 2.60 (s, 6H); **$^{13}$C NMR (151 MHz, MeOD)** δ 160.68, 122.47, 110.26, 100.60, 70.66, 66.76, 59.72, 53.68, 51.53f, 43.21; **HRMS** (ESI) m/z: [M+H]+ calcd for $C_{22}H_{37}BrN_4O_4$: 501.20710, found 501.20713.

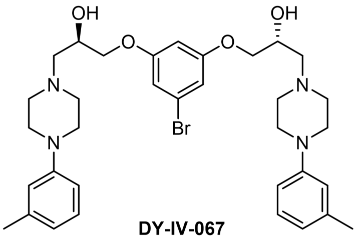

**DY-IV-067**
**31**

**(2R,2'R)-3,3'-((5-bromo-1,3-phenylene)bis(oxy))bis(1-(4-(m-tolyl)piperazin-1-yl)propan-2-ol) (DY-IV-067) (31):** Was synthesized according to general procedure A with epoxide **8** and 1-(m-tolyl)piperazine as the amine and isolated as a clear oil (2.71 mg). **$^1$H NMR (500 MHz, MeOD)** δ 7.20 – 7.12 (m, 3H), 6.88 – 6.72 (m, 6H), 6.59 (t, *J* = 2.1 Hz, 1H), 4.45 (dd, *J* = 9.7, 4.6 Hz, 2H), 4.02 (dd, *J* = 5.0, 2.3 Hz, 4H), 3.76 (m, 8H), 3.70 – 3.62 (m, 2H), 3.45 – 3.34 (m, 8H), 3.22 – 3.12 (m, 2H), 2.31 (d, *J* = 6.3 Hz, 6H); **$^{13}$C**

**NMR (126 MHz, MeOD)** δ 160.39, 149.94, 139.00, 128.94, 122.01, 117.46, 113.88, 110.89, 100.74, 70.23, 63.80, 58.66, 46.56, 43.60, 20.43; **HRMS** (ESI) m/z: [M+H]+ calcd for $C_{34}H_{46}BrN_4O_4$: 653.26969, found 653.26969.

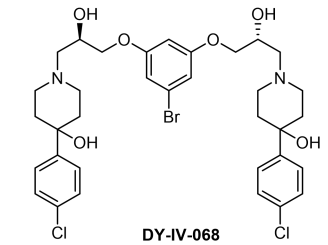

**DY-IV-068**
**32**

**1,1'-((2R,2'R)-((5-bromo-1,3-phenylene)bis(oxy))bis(2-hydroxypropane-3,1-diyl))bis(4-(4-chlorophenyl)piperidin-4-ol) (DY-IV-068) (32):** Was synthesized according to general procedure A with epoxide **8** and 4-(4-chlorophenyl)piperidin-4-ol as the amine and isolated as a clear oil (4.35 mg). **$^1$H NMR (500 MHz, MeOD)** δ 7.51 (t, *J* = 8.3 Hz, 4H), 7.42 – 7.36 (m, 4H), 6.82 (d, *J* = 2.1 Hz, 2H), 6.60 (s, 1H), 4.46 (dd, *J* = 9.9, 4.6 Hz, 2H), 4.02 (d, *J* = 5.0 Hz, 4H), 3.67 – 3.33 (m, 10H), 2.47 – 2.29 (m, 4H), 2.20 (td, *J* = 13.9, 4.6 Hz, 2H), 1.96 – 1.89 (m, 6H); **$^{13}$C NMR (151 MHz, $CDCl_3$)** δ 161.72, 147.04, 134.36, 129.58, 127.46, 124.06, 112.16, 102.04, 71.56, 69.52, 69.13, 65.11, 60.51, 52.23, 41.64, 36.53, 35.83; **HRMS** (ESI) m/z: [M+H]+ calcd for $C_{34}H_{42}BrCl_2N_2O_6$: 723.15978, found 723.1598.

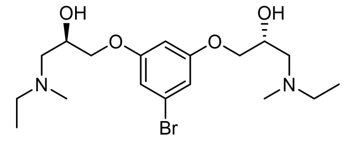

**DY-IV-048**
**33**

**(2R,2'R)-3,3'-((5-bromo-1,3-phenylene)bis(oxy))bis(1-(ethyl(methyl)amino)propan-2-ol) (DY-IV-048) (33):** Was synthesized according to general procedure A with epoxide **6.63** and *N*-methylethanamine as the amine and isolated as a clear oil (2.65 mg). **$^1$H NMR (500 MHz, MeOD)**

δ 6.80 (d, *J* = 2.2 Hz, 2H), 6.57 (t, *J* = 2.2 Hz, 1H), 4.37 – 4.31 (m, 2H), 4.00 (d, *J* = 5.2 Hz, 4H), 3.44 – 3.25 (m, 6H), 2.93 (d, *J* = 13.5 Hz, 6H), 1.37 (t, *J* = 7.3 Hz, 6H); **$^{13}$C NMR (126 MHz, MeOD)** δ 160.39, 122.77, 110.80, 100.70, 70.16, 64.14, 63.68, 57.58, 56.71, 52.98, 49.91, 40.61, 38.51, 8.13, 7.82; **HRMS** (ESI) m/z: [M+H]+ calcd for $C_{18}H_{32}BrN_2O_4$: 419.15399, found 419.15423.

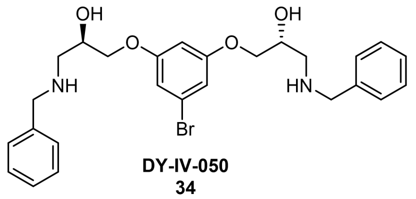


**DY-IV-050**
**34**

**(2R,2'R)-3,3'-((5-bromo-1,3-phenylene)bis(oxy))bis(1-(benzylamino)propan-2-ol) (DY-IV-050) (34):** Was synthesized according to general procedure A with epoxide **8** and benzylamine as the amine and isolated as a yellow oil (1.37 mg). **$^{1}$H NMR (600 MHz, MeOD)** δ 7.51 (dq, *J* = 5.3, 2.8 Hz, 4H), 7.51 – 7.44 (m, 6H), 6.75 (d, *J* = 2.2 Hz, 2H), 6.50 (t, *J* = 2.2 Hz, 1H), 4.28 (s, 4H), 4.25 (m, 2H), 3.97 (qd, *J* = 9.9, 5.2 Hz, 4H), 3.26 (dd, *J* = 12.8, 3.1 Hz, 2H), 3.13 (dd, *J* = 12.7, 9.8 Hz, 2H); **$^{13}$C NMR (151 MHz, MeOD)** δ 160.20, 130.87, 129.72, 129.37, 128.94, 122.58, 110.68, 100.51, 69.94, 65.01, 50.89, 48.92; **HRMS** (ESI) m/z: [M+H]+ calcd for $C_{26}H_{32}BrN_2O_4$: 515.1545, found 515.1536.

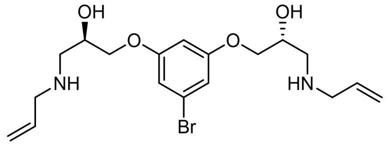


**DY-IV-051**
**35**

**(2R,2'R)-3,3'-((5-bromo-1,3-phenylene)bis(oxy))bis(1-(allylamino)propan-2-ol) (DY-IV-051) (35):** Was synthesized according to general procedure A with epoxide **8** and allyl amine as the amine and isolated as a yellow oil (1.05

mg). **$^1$H NMR (600 MHz, MeOD)** δ 6.80 (d, *J* = 2.3 Hz, 2H), 6.55 (t, *J* = 2.2 Hz, 1H), 5.96 (ddt, *J* = 17.1, 10.3, 6.9 Hz, 2H), 5.57 – 5.50 (m, 4H), 4.22 (dtd, *J* = 8.3, 5.1, 3.1 Hz, 2H), 3.99 (qd, *J* = 9.8, 5.1 Hz, 4H), 3.71 (dd, *J* = 6.9, 1.2 Hz, 4H), 3.26 (dd, *J* = 12.7, 3.1 Hz, 2H), 3.13 (dd, *J* = 12.7, 9.7 Hz, 2H); **$^{13}$C NMR (151 MHz, MeOD)** δ 160.24, 127.68, 123.21, 122.64, 110.64, 100.61, 69.96, 65.12, 49.57, 48.44; **HRMS** (ESI) m/z: [M+H]+ calcd for $C_{18}H_{28}BrN_2O_4$: 415.1232, found 415.1233.

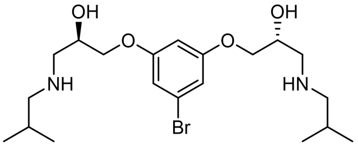


**DY-IV-052**
**36**

**(2R,2'R)-3,3'-((5-bromo-1,3-phenylene)bis(oxy))bis(1-(isobutylamino)propan-2-ol) (DY-IV-052) (36):** Was synthesized according to general procedure A with epoxide **8** and 2-methylpropan-1-amine as the amine and isolated as a clear oil (1.21 mg). **$^1$H NMR (500 MHz, MeOD)** δ 6.79 (d, *J* = 2.2 Hz, 2H), 6.56 (t, *J* = 2.2 Hz, 1H), 4.27 (dtd, *J* = 10.0, 5.1, 3.1 Hz, 2H), 4.04 – 3.95 (m, 4H), 3.28 (dd, *J* = 12.8, 3.1 Hz, 2H), 3.14 (dd, *J* = 12.8, 10.0 Hz, 2H), 2.99 – 2.87 (m, 4H), 2.07 (dp, *J* = 13.7, 6.8 Hz, 2H), 1.05 (dd, *J* = 6.7, 4.3 Hz, 12H); **$^{13}$C NMR (126 MHz, MeOD)** δ 160.40, 122.74, 110.77, 100.67, 70.14, 65.00, 54.81, 50.05, 25.66, 19.06, 18.89; **HRMS** (ESI) m/z: [M+H]+ calcd for $C_{20}H_{36}BrN_2O_4$: 447.1858, found 447.1854.

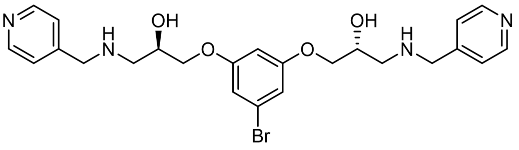


**DY-IV-053**
**37**

**(2R,2'R)-3,3'-((5-bromo-1,3-phenylene)bis(oxy))bis(1-((pyridin-4-ylmethyl)amino)propan-2-ol) (DY-IV-053) (37):** Was synthesized according

to general procedure A with epoxide **8** and pyridin-4-ylmethanamine as the amine and isolated as a light brown oil (2.79 mg). **$^1$H NMR (500 MHz, MeOD)** δ 8.56 (m, 4H), 7.50 (m, 4H), 6.74 (d, *J* = 2.1 Hz, 2H), 6.49 (s, 1H), 4.14 (s, 2H), 4.08 (m, 4H), 3.98 (m, 5H), 3.01 (d, *J* = 12.2 Hz, 2H), 2.92 (d, *J* = 10.4 Hz, 2H); **$^{13}$C NMR (151 MHz, MeOD)** δ 160.48, 149.00, 123.94, 123.88, 122.52, 110.46, 100.48, 70.29, 67.19, 50.88, 29.26; **HRMS** (ESI) m/z: [M+H]+ calcd for $C_{24}H_{30}BrN_4O_4$: 517.1445, found 517.1445.

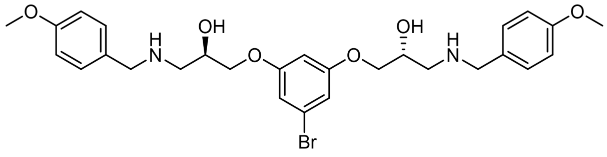


**DY-IV-063**
**38**

**(2R,2'R)-3,3'-((5-bromo-1,3-phenylene)bis(oxy))bis(1-((4-methoxybenzyl)amino)propan-2-ol) (DY-IV-063) (38):** Was synthesized according to general procedure A with epoxide **8** and (4-methoxyphenyl)methanamine as the amine and isolated as a clear oil (2.28 mg). **$^1$H NMR (500 MHz, MeOD)** δ 7.43 (d, *J* = 8.2 Hz, 4H), 6.99 (t, *J* = 7.9 Hz, 4H), 6.75 (d, *J* = 1.9 Hz, 2H), 6.50 (s, 1H), 4.22 (m, 2H), 4.21 (s, 4H), 3.96 (dd, *J* = 9.1, 4.9 Hz, 4H), 3.81 (s, 6H), 3.22 (d, *J* = 11.6 Hz, 2H), 3.08 (t, *J* = 11.1 Hz, 2H); **$^{13}$C NMR (151 MHz, MeOD)** δ 160.75, 160.22, 131.27, 130.13, 122.55, 120.34, 114.16, 110.69, 100.48, 69.97, 65.09, 54.44, 54.40, 50.42; **HRMS** (ESI) m/z: [M+H]+ calcd for $C_{28}H_{36}BrN_2O_6$: 575.17512, found 575.17493.

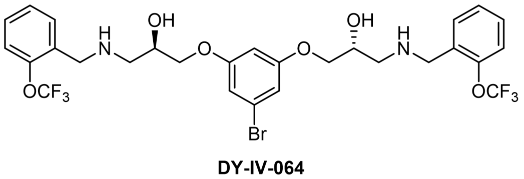


**DY-IV-064**
**39**

**(2R,2'R)-3,3'-((5-bromo-1,3-phenylene)bis(oxy))bis(1-((4-(trifluoromethoxy)benzyl)amino)propan-2-ol)**

**(DY-IV-064 ) (39):** Was synthesized according to general procedure A with epoxide **8** and (2-(trifluoromethoxy)phenyl)methanamine as the amine and isolated as a clear oil (1.55 mg). **$^{1}$H NMR (500 MHz, MeOD)** δ 7.66 (d, *J* = 7.6 Hz, 2H), 7.55 (t, *J* = 7.8 Hz, 2H), 7.49 – 7.41 (m, 4H), 6.76 (d, *J* = 2.0 Hz, 2H), 6.52 (s, 1H), 4.30 (m, 4H), 4.24 (m, 2H), 3.98 (s, 4H), 3.21 (d, *J* = 12.5 Hz, 2H), 3.10 (d, *J* = 10.8 Hz, 2H); **$^{13}$C NMR (126 MHz, MeOD)** δ 161.70, 159.93, 149.22, 132.87, 132.23, 128.78, 123.96, 122.92 (d, $J_{CF}$ = 247.97 Hz) 121.69, 112.01, 101.88, 71.49, 54.47, 53.75, 51.14. **HRMS** (ESI) m/z: [M+H]+ calcd for $C_{28}H_{30}BrF_6N_2O_6$: 683.11859, found 683.11872.

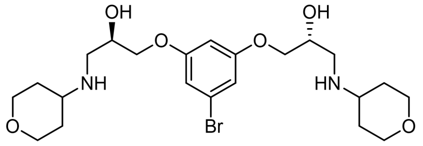


**DY-IV-066**
**40**

**(2R,2'R)-3,3'-((5-bromo-1,3-phenylene)bis(oxy))bis(1-((tetrahydro-2H-pyran-4-yl)amino)propan-2-ol)**

**(DY-IV-066) (40):** Was synthesized according to general procedure A with epoxide **8** and tetrahydro-2H-pyran-4-amine as the amine and isolated as a clear oil (6.61 mg). **$^{1}$H NMR (500 MHz, MeOD)** δ 6.79 (d, *J* = 2.0 Hz, 2H), 6.57 (s, 1H), 4.23 (d, *J* = 8.3 Hz, 2H), 4.02 (m, 8H), 3.44 (t, *J* = 11.9 Hz, 4H), 3.36 (d, *J* = 13.4 Hz, 2H), 3.30 (m, 2H), 3.14 (dd, *J* = 12.5, 9.7 Hz, 2H), 2.09 – 2.00 (m, 4H), 1.71 (tdd, *J* = 17.4, 12.0, 4.8 Hz, 4H); **$^{13}$C NMR (126 MHz, MeOD)** δ 160.43, 122.71, z110.77, 100.68, 70.15, 65.76, 65.57, 54.56, 46.75, 29.45, 29.12; **HRMS** (ESI) m/z: [M+H]+ calcd for $C_{22}H_{36}BrN_2O_6$: 503.1751, found 503.1789.

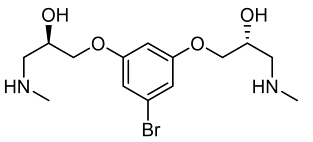

**DY-IV-044**
**41**

**(2R,2'R)-3,3'-((5-bromo-1,3-phenylene)bis(oxy))bis(1-(methylamino)propan-2-ol)**

**(DY-IV-044) (41):** Was synthesized according to general procedure A with epoxide **6.63** and methylamine as the amine and isolated as a clear oil (2.47 mg). **$^{1}$H NMR (500 MHz, MeOD)** δ 6.80 (d, *J* = 2.2 Hz, 2H), 6.56 (t, *J* = 2.2 Hz, 1H), 4.22 (dtd, *J* = 9.7, 5.1, 3.3 Hz, 2H), 4.04 – 3.95 (m, 4H), 3.25 (dd, *J* = 12.7, 3.2 Hz, 2H), 3.14 (dd, *J* = 12.7, 9.6 Hz, 2H), 2.76 (s, 6H); **$^{13}$C NMR (126 MHz, MeOD)** δ 160.39, 122.74, 110.79, 100.67, 70.06, 65.13, 51.35, 32.60; **HRMS** (ESI) m/z: [M+H]+ calcd for $C_{14}H_{34}BrN_2O_4$: 363.09140, found 363.09149.

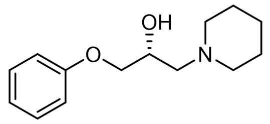

**DY-III-273**
**42**

**(R)-1-phenoxy-3-(piperidin-1-yl)propan-2-ol (DY-III-273) (42):** Was synthesized according to General Procedure B with epoxide **9** and piperidine as the amine and isolated as a clear oil (2.47 mg). **$^{1}$H NMR (500 MHz, MeOD)** δ 7.30 – 7.22 (m, 2H), 6.97 – 6.89 (m, 3H), 4.15 (ddd, *J* = 10.0, 8.3, 4.6 Hz, 1H), 3.97 (dd, *J* = 9.8, 4.4 Hz, 1H), 3.91 (dd, *J* = 9.8, 5.8 Hz, 1H), 2.61 (m, 6H), 1.65 (m, 4H), 1.50 (m, 2H); **$^{13}$C NMR (126 MHz, MeOD)** δ 159.06, 129.20, 120.64, 114.31, 70.64, 66.85, 61.65, 54.84, 25.20, 23.70; **HRMS** (ESI) m/z: [M+H]+ calcd for $C_{14}H_{22}NO_2$: 236.1645, found 236.1646.

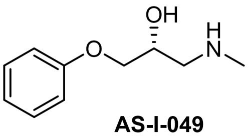


**AS-I-049**
**43**

**(R)-1-(methylamino)-3-phenoxypropan-2-ol (AS-I-049) (43):** Was synthesized according to general procedure B with epoxide **9** and methylamine as the amine and isolated as a clear oil (1.89 mg). **$^1$H NMR (500 MHz, MeOD)** δ 7.32 – 7.25 (m, 2H), 6.99 – 6.92 (m, 3H), 4.23 (ddd, *J* = 8.9, 5.4, 2.7 Hz, 1H), 4.04 (dd, *J* = 9.8, 5.0 Hz, 1H), 3.98 (dd, *J* = 9.8, 5.4 Hz, 1H), 3.28 (dd, *J* = 12.7, 3.3 Hz, 1H), 3.16 (dd, *J* = 12.7, 9.4 Hz, 1H), 2.76 (s, 3H); **$^{13}$C NMR (151 MHz, MeOD)** δ 158.49, 129.18, 120.96, 114.18, 69.44, 65.18, 51.49, 32.52; **HRMS** (ESI) m/z: [M+H]+ calcd for $C_{10}H_{16}NO_2$: 182.11748, found 182.11750.

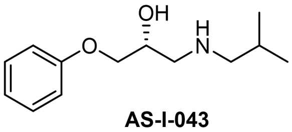


**AS-I-043**
**44**

**(R)-1-(isobutylamino)-3-phenoxypropan-2-ol (AS-I-043) (44):** Was synthesized according to general procedure B with epoxide **9** and 2-methylpropan-1-amine (1.89 mg). **$^1$H NMR (500 MHz, MeOD)** δ 7.32 – 7.25 (m, 2H), 6.96 (dd, *J* = 8.1, 6.6 Hz, 3H), 4.27 (dt, *J* = 8.7, 4.6 Hz, 1H), 4.05 (dd, *J* = 9.8, 5.0 Hz, 1H), 3.99 (dd, *J* = 9.9, 5.5 Hz, 1H), 3.16 (dd, *J* = 12.8, 9.9 Hz, 1H), 2.93 (dd, *J* = 7.3, 5.2 Hz, 2H), 2.07 (dp, *J* = 13.8, 6.9 Hz, 1H), 1.06 (dd, *J* = 6.7, 4.3 Hz, 6H); **$^{13}$C NMR (151 MHz, MeOD)** δ 158.25, 129.35, 121.24, 114.21, 69.47, 64.99, 54.90, 50.39, 25.61, 19.27; **HRMS** (ESI) m/z: [M+H]+ calcd for $C_{13}H_{22}NO_2$: 224.1652, found 224.1651.

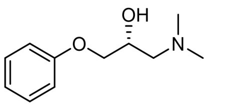

**AS-I-042**
**45**

**(R)-1-(dimethylamino)-3-phenoxypropan-2-ol (AS-I-042) (45):** Was synthesized according to general procedure B with epoxide **9** and dimethylamine as the amine and isolated as a clear oil (2.14 mg). **$^{1}$H NMR (500 MHz, MeOD)** δ 7.28 (t, *J* = 7.9 Hz, 2H), 6.99 – 6.92 (m, 3H), 4.35 (dq, *J* = 10.4, 5.3 Hz, 1H), 4.01 (qd, *J* = 9.8, 5.1 Hz, 2H), 3.37 – 3.32 (m, 2H), 2.96 (s, 6H); **$^{13}$C NMR (126 MHz, MeOD)** δ 158.63, 129.33, 121.09, 114.31, 69.63, 64.07, 59.74; **HRMS** (ESI) m/z: [M+H]+ calcd for $C_{11}H_{18}NO_2$: 196.1333, found 196.1338.

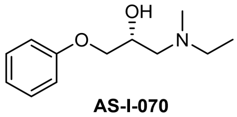

**AS-I-070**
**46**

**(R)-1-(diethylamino)-3-phenoxypropan-2-ol (AS-I-070) (46):** Was synthesized according to general procedure B with epoxide **9** and *N*-methylethanamine as the amine and isolated as a clear oil (3.16 mg). **$^{1}$H NMR (500 MHz, MeOD)** δ 7.29 (t, *J* = 7.9 Hz, 2H), 6.96 (d, *J* = 7.8 Hz, 3H), 4.35 (m, 1H), 4.01 (dt, *J* = 15.2, 5.3 Hz, 2H), 3.47 – 3.34 (m, 4H), 2.94 (d, *J* = 12.6 Hz, 3H), 1.37 (t, *J* = 7.4 Hz, 3H); **$^{13}$C NMR (151 MHz, MeOD)** δ 158.48, 129.20, 121.01, 114.17, 69.53, 63.71, 56.89, 52.84, 38.44, 8.02; **HRMS** (ESI) m/z: [M+H]+ calcd for $C_{12}H_{20}NO_2$: 210.1489, found 210.1491.

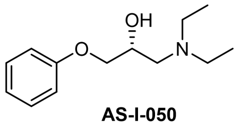

**AS-I-050**
**47**

**(R)-1-(diethylamino)-3-phenoxypropan-2-ol (AS-I-050) (47):** Was synthesized according to general procedure B with epoxide **9** and

diethylamine as the amine and isolated as a clear oil (3.80 mg). **$^{1}$H NMR (500 MHz, MeOD)** δ 7.29 (dd, *J* = 8.6, 7.4 Hz, 2H), 7.00 – 6.93 (m, 3H), 4.33 (s, 1H), 4.08 – 3.96 (m, 2H), 3.40 (m, 4H), 1.36 (td, *J* = 7.3, 2.5 Hz, 6H); **$^{13}$C NMR (151 MHz, MeOD)** δ 158.44, 129.21, 121.03, 114.17, 69.52, 64.09, 54.14, 48.92, 7.77; **HRMS** (ESI) m/z: [M+H]+ calcd for $C_{13}H_{22}NO_2$: 224.1645, found 224.1645.

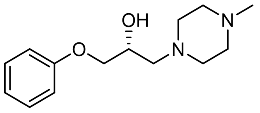

**AS-I-058**
**48**

**(R)-1-(4-methylpiperazin-1-yl)-3-phenoxypropan-2-ol (AS-I-058) (48):** Was synthesized according to general procedure B with epoxide **9** and *N*-methylpiperizine as the amine and isolated as a clear oil (1.16 mg). **$^{1}$H NMR (500 MHz, MeOD)** δ 7.27 (dd, *J* = 8.6, 7.1 Hz, 2H), 6.97 – 6.91 (m, 3H), 4.29 – 4.23 (m, 1H), 4.01 (dd, *J* = 4.9, 1.6 Hz, 2H), 3.43 – 3.38 (m, 4H), 3.27 (m, 4H), 3.09 - 3.03 (m, 2H), 2.89 (s, 3H); **$^{13}$C NMR (126 MHz, MeOD)** δ 158.84, 129.27, 120.90, 114.31, 69.88, 65.90, 59.16, 51.95, 48.59, 42.35; **HRMS** (ESI) m/z: [M+H]+ calcd for $C_{14}H_{23}N_2O_2$: 251.1763, found 251.1754.

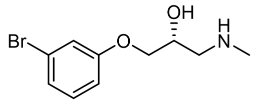

**AS-I-081**
**49**

**(R)-1-(3-bromophenoxy)-3-(methylamino)propan-2-ol (AS-I-081) (49):** Was synthesized according to general procedure B with epoxide **10** and methyl amine as the amine and isolated as a clear oil (1.82 mg). **$^{1}$H NMR (500 MHz, MeOD)** δ 7.21 (t, *J* = 8.1 Hz, 1H), 7.18 – 7.10 (m, 2H), 6.95 (ddd, *J* = 8.3, 2.5, 0.9 Hz, 1H), 4.26 – 4.18 (m, 1H), 4.01 (qd, *J* = 9.8, 5.1 Hz, 2H), 3.26 (dd, *J* = 12.7, 3.3 Hz, 1H), 3.16 (dd, *J* = 12.7, 9.5 Hz, 1H), 2.76 (s, 3H); **$^{13}$C NMR (151**

**MHz, MeOD)** δ 159.37, 130.58, 124.05, 122.35, 117.64, 113.20, 69.80, 65.01, 51.28, 32.49; **HRMS** (ESI) m/z: [M+H]+ calcd for $C_{10}H_{15}BrNO_2$: 260.02902, found 260.02809.

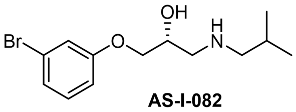


**AS-I-082**
**50**

**(R)-1-(3-bromophenoxy)-3-(isobutylamino)propan-2-ol (AS-I-082) (50):** Was synthesized according to general procedure B with epoxide **10** and 2-methylpropan-1-amine as the amine and isolated as a clear oil (1.29 mg). **$^1$H NMR (500 MHz, MeOD)** δ 7.21 (t, *J* = 8.1 Hz, 1H), 7.18 – 7.10 (m, 2H), 6.98 – 6.92 (m, 1H), 4.27 (s, 1H), 4.07 – 3.97 (m, 2H), 3.27 - 3.15 (dd, *J* = 12.7, 10.0 Hz, 2H), 2.93 (dd, *J* = 7.2, 5.9 Hz, 2H), 2.07 (dt, *J* = 13.7, 6.8 Hz, 1H), 1.06 (dd, *J* = 6.7, 4.3 Hz, 6H). **$^{13}$C NMR (151 MHz, MeOD)** δ 159.37, 130.59, 124.05, 122.36, 117.61, 113.21, 69.88, 64.87, 54.68, 49.96, 25.52, 18.91; **HRMS** (ESI) m/z: [M+H]+ calcd for $C_{13}H_{21}BrNO_2$: 301.0630, found 301.0688.

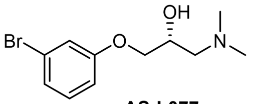


**AS-I-077**
**51**

**(R)-1-(3-bromophenoxy)-3-(dimethylamino)propan-2-ol (AS-I-077) (51):** Was synthesized according to general procedure B with epoxide **10** and dimethylamine as the amine and isolated as a clear oil (1.12 mg). Compound **51** was judged to be >95% pure by LCMS analysis. **$^1$H NMR (500 MHz, MeOD)** δ 7.25 – 7.11 (m, 3H), 6.95 (dd, *J* = 8.0, 2.5 Hz, 1H), 4.38 – 4.30 (m, 1H), 4.01 (dd, *J* = 4.9, 2.2 Hz, 2H), 2.96 (d, *J* = 15.2 Hz, 6H); **$^{13}$C NMR (151 MHz, MeOD)** δ 159.39, 130.58, 124.04, 122.34, 117.65, 113.20, 69.88, 63.84,

59.42, 42.07; **HRMS** (ESI) m/z: [M+H]+ calcd for $C_{11}H_{17}BrNO_2$: 274.04446, found 274.04372.

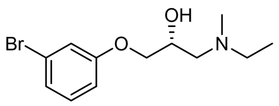

**AS-I-078**
**52**

**(R)-1-(3-bromophenoxy)-3-(ethyl(methyl)amino)propan-2-ol (AS-I-078) (52):** Was synthesized according to general procedure B with epoxide **10** and *N*-methylethanamine as the amine and isolated as a clear oil (1.00 mg). **$^1$H NMR (500 MHz, MeOD)** δ 7.25 – 7.11 (m, 3H), 6.96 (d, *J* = 8.4 Hz, 1H), 4.35 (s, 1H), 4.02 (d, *J* = 5.2 Hz, 2H), 3.4 – 3.3 (m, 2H), 2.93 (d, *J* = 13.5 Hz, 3H), 1.37 (t, *J* = 7.3 Hz, 3H); **$^{13}$C NMR (151 MHz, MeOD)** δ 159.39, 130.58, 124.04, 122.35, 117.65, 113.22, 69.93, 63.85, 57.09, 51.57, 39.31, 7.90; **HRMS** (ESI) m/z: [M+H]+ calcd for $C_{12}H_{19}BrNO_2$: 288.060212, found 288.059368.

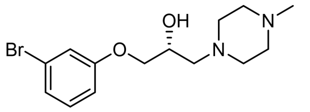

**AS-I-086**
**53**

**(R)-1-(3-bromophenoxy)-3-(4-methylpiperazin-1-yl)propan-2-ol (AS-I-086) (53):** Was synthesized according to general procedure B with epoxide **10** and *N*-methylpiperizine as the amine and isolated as a clear oil (1.82 mg). **$^1$H NMR (500 MHz, MeOD)** δ 7.20 (t, *J* = 8.1 Hz, 1H), 7.16 – 7.08 (m, 2H), 6.97 – 6.91 (m, 1H), 4.17 (dd, *J* = 8.2, 4.6 Hz, 1H), 4.03 (dd, *J* = 9.8, 4.3 Hz, 1H), 3.98 (dd, *J* = 9.8, 5.5 Hz, 1H), 3.46 (d, *J* = 11.0 Hz, 1H), 3.02 (m, 6H), 2.85 (s, 3H). **$^{13}$C NMR (151 MHz, MeOD)** δ 159.74, 130.51, 123.72, 122.31, 117.51, 113.29, 70.30, 66.45, 59.08, 52.89, 50.49, 42.30; **HRMS** (ESI) m/z: [M+H]+ calcd for $C_{13}H_{22}BrN_2O_2$: 329.08668, found 329.08592.

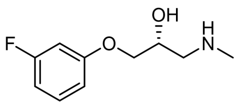

**AS-I-079**
**54**

**(R)-1-(3-fluorophenoxy)-3-(methylamino)propan-2-ol (AS-I-079) (54):** Was synthesized according to general procedure B with epoxide **11** and methylamine as the amine and isolated as a clear oil (2.66 mg).

**$^1$H NMR (500 MHz, MeOD)** δ 7.29 (q, *J* = 8.0 Hz, 1H), 6.79 (dd, *J* = 8.3, 2.4 Hz, 1H), 6.77 – 6.68 (m, 2H), 4.26 – 4.19 (m, 1H), 4.02 (qd, *J* = 9.9, 5.2 Hz, 2H), 3.29 – 3.16 (m, 2H), 3.16, 2.76 (s, 3H); **$^{13}$C NMR (151 MHz, MeOD)** δ 164.46 (d, $J_{CF}$ = 244.4 Hz), 159.89 (d, $J_{CF}$ = 13.4 Hz), 130.30 (d, $J_{CF}$ = 10.86 Hz), 110.10 (d, $J_{CF}$ = 2.89 Hz), 107.59 (d, $J_{CF}$ = 18.9 Hz), 101.91 (d, $J_{CF}$ = 25.6 Hz), 69.81, 65.01, 51.30, 32.49; **HRMS** (ESI) m/z: [M+H]+ calcd for $C_{10}H_{15}FNO_2$: 200.10881, found 200.10813.

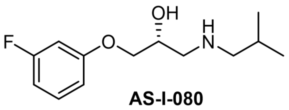

**AS-I-080**
**55**

**(R)-1-(3-fluorophenoxy)-3-(isobutylamino)propan-2-ol (AS-I-080) (55):** Was synthesized according to general procedure B with epoxide **11** and 2-methylpropan-1-amine as the amine and isolated as a clear oil (2.76 mg). Compound **55** was judged to be >95% pure by LCMS analysis. **$^1$H NMR (500 MHz, MeOD)** δ 7.33 – 7.26 (m, 1H), 6.80 (d, *J* = 8.5 Hz, 1H), 6.73 (q, *J* = 10.1 Hz, 2H), 4.26 (m, 1H), 4.03 (m, 2H), 3.20 (d, *J* = 12.5 Hz, 1H), 3.07 (t, *J* = 11.6 Hz, 1H), 2.85 (m, 2H), 2.06 (d, *J* = 13.5 Hz, 1H), 1.31(d, *J* = 5.9 Hz, 6H); **$^{13}$C NMR (151 MHz, MeOD)** δ 164.9 (d, $J_{CF}$ = 245 Hz), 130.27 (d, $J_{CF}$ = 11.55 Hz), 110.09 (d, $J_{CF}$ = 3.04 Hz), 107.46 (d, $J_{CF}$ = 19.66 Hz), 101.87 (d, $J_{CF}$ =

25.38 Hz), 70.11, 55.28, 55.46, 29.2, 25.00, 19.06; **HRMS** (ESI) m/z: [M+H]+ calcd for $C_{13}H_{20}FNO_2$: 242.15586, found 242.15588.

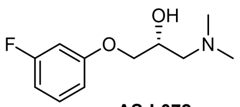

AS-I-072
56

**(R)-1-(dimethylamino)-3-(3-fluorophenoxy)propan-2-ol (AS-I-072) (56):** Was synthesized according to general procedure B with epoxide **11** and dimethylamine as the amine and isolated as a clear oil (2.01 mg). **$^1$H NMR (500 MHz, MeOD)** δ 7.33 – 7.25 (m, 1H), 6.79 (d, *J* = 8.3 Hz, 1H), 6.77 – 6.68 (m, 2H), 4.34 (dd, *J* = 8.4, 5.3 Hz, 1H), 4.01 (dd, *J* = 5.0, 3.0 Hz, 2H), 3.39 – 3.32 (m, 2H), 2.96 (s, 6H); **$^{13}$C NMR (151 MHz, MeOD)** δ 164.46 (d, $J_{CF}$ = 244.4 Hz), 159.97 (d, $J_{CF}$ = 11.0 Hz), 130.25 (d, $J_{CF}$ = 10.0 Hz), , 110.09 (d, $J_{CF}$ = 2.79 Hz), 107.62 (d, $J_{CF}$ = 21.49 Hz), 101.91 (d, $J_{CF}$ = 25.9 Hz), 69.87, 63.75, 59.40, 48.16; **HRMS** (ESI) m/z: [M+H]+ calcd for $C_{11}H_{16}FNO_2$: 214.1244, found 214.12378.

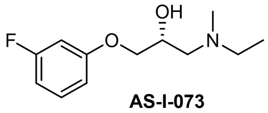

AS-I-073
57

**(R)-1-(3-bromophenoxy)-3-(ethyl(methyl)amino)propan-2-ol (AS-I-078) (57):** Was synthesized according to general procedure B with epoxide **11** and *N*-methylethanamine as the amine and isolated as a clear oil (1.00 mg). Compound **57** was judged to be >95% pure by LCMS analysis. **$^1$H NMR (500 MHz, MeOD)** δ 7.25 – 7.11 (m, 3H), 6.96 (d, *J* = 8.4 Hz, 1H), 4.35 (s, 1H), 4.02 (d, *J* = 5.2 Hz, 2H), 3.4 – 3.3 (m, 2H), 2.93 (d, *J* = 13.5 Hz, 3H), 1.37 (t, *J* = 7.3 Hz, 3H); **$^{13}$C NMR (151 MHz, MeOD)** δ 159.39, 130.58, 124.04, 122.35,

117.65, 113.22, 69.93, 63.85, 57.09, 51.57, 39.31, 7.90; **HRMS** (ESI) m/z: [M+H]+ calcd for $C_{12}H_{19}BrNO_2$: 288.060212, found 288.059368.

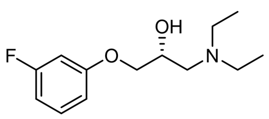

**AS-I-083**
**58**

**(R)-1-(diethylamino)-3-(3-fluorophenoxy)propan-2-ol (AS-I-083) (58):** Was synthesized according to general procedure B with epoxide **11** and diethylamine as the amine and isolated as a clear oil (1.00 mg). **$^1$H NMR (500 MHz, MeOD)** δ 7.29 (q, *J* = 8.1 Hz, 1H), 6.79 (dd, *J* = 8.6, 2.3 Hz, 1H), 6.77 – 6.68 (m, 2H), 4.34 (dd, *J* = 9.9, 3.9 Hz, 1H), 4.07 – 3.98 (m, 2H), 3.42 – 3.33 (m, 6H), 1.36 (t, *J* = 7.3 Hz, 6H). **$^{13}$C NMR (151 MHz, MeOD)** δ 164.50 (d, $J_{CF}$ = 244.2 Hz), 159.8 (d, $J_{CF}$ = 12.73 Hz), 130.29 (d, $J_{CF}$ = 9.86 Hz), 110.15 (d, $J_{CF}$ = 3.36 Hz), 107.19 (d, $J_{CF}$ = 20.55 Hz), 101.41 (d, $J_{CF}$ = 25.4 Hz), 69.92, 63.97, 53.97, 48.16, 7.89; **HRMS** (ESI) m/z: [M+H]+ calcd for $C_{13}H_{21}FNO_2$: 242.15583, found 242.15508.

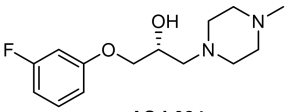

**AS-I-084**
**59**

**(R)-1-(3-fluorophenoxy)-3-(4-methylpiperazin-1-yl)propan-2-ol (AS-I-084) (59):** Was synthesized according to general procedure B with epoxide **11** and *N*-methylpiperizine as the amine and isolated as a clear oil (1.10 mg). **$^1$H NMR (500 MHz, MeOD)** δ 7.31 – 7.23 (m, 1H), 6.80 – 6.65 (m, 3H), 4.17 (d, *J* = 7.8 Hz, 1H), 4.06 – 3.95 (m, 2H), 3.46 (m, 6H), 3.03 (s, 4H), 2.85 (s, 6H). **$^{13}$C NMR (151 MHz, MeOD)** δ 164.47 (d, $J_{CF}$ = 242.9 Hz), 160.35 (d, $J_{CF}$ = 10.8 Hz), 130.21 (d, $J_{CF}$ = 9.9 Hz), 110.15 (d, $J_{CF}$ = 2.84 Hz), 107.11 (d, $J_{CF}$ = 19.46

Hz), 101.81 (d, $J_{CF}$ = 25.38 Hz), 70.32, 66.43, 59.11, 52.86, 50.45, 42.31; **HRMS** (ESI) m/z: [M+H]+ calcd for $C_{14}H_{22}FN_2O_2$: 269.16684, found 269.16598.

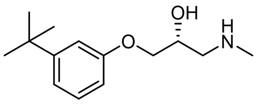

**AS-I-060**
**60**

**(R)-1-(3-(tert-butyl)phenoxy)-3-(methylamino)propan-2-ol (AS-I-060) (60):** Was synthesized according to general procedure B with epoxide **12** and methylamine as the amine and isolated as a clear oil (2.83 mg). **$^1$H NMR (500 MHz, MeOD)** δ 7.21 (t, *J* = 8.0 Hz, 1H), 7.05 – 6.97 (m, 2H), 6.76 (d, *J* = 8.2 Hz, 1H), 4.22 (s, 1H), 4.04 (t, *J* = 7.3 Hz, 1H), 3.98 (t, *J* = 7.8 Hz, 1H), 3.30 - 3.17 (m, 2H), 2.76 (s, 3H), 1.30 (s, 9H); **$^{13}$C NMR (151 MHz, MeOD)** δ 158.34, 152.82, 128.75, 118.11, 112.01, 110.57, 69.44, 65.22, 51.54, 34.18, 32.52, 30.31; **HRMS** (ESI) m/z: [M+H]+ calcd for $C_{14}H_{24}NO_2$: 238.1807, found 228/1802.

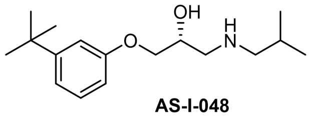

**AS-I-048**
**61**

**(R)-1-(3-(tert-butyl)phenoxy)-3-(isobutylamino)propan-2-ol (AS-I-048) (61):** Was synthesized according to general procedure B with epoxide **12** and 2-methylpropan-1-amine as the amine and isolated as a clear oil (1.62 mg). **$^1$H NMR (500 MHz, MeOD)** δ 7.21 (t, *J* = 8.0 Hz, 1H), 7.02 (dt, *J* = 7.9, 1.3 Hz, 1H), 6.98 (t, *J* = 2.2 Hz, 1H), 6.76 (dd, *J* = 8.0, 2.5 Hz, 1H), 4.27 (s, 1H), 4.05 (dd, *J* = 9.8, 5.0 Hz, 1H), 3.99 (dd, *J* = 9.8, 5.5 Hz, 1H), 3.29 (m, 2H), 3.21 – 3.13 (m, 2H), 2.07 (dt, *J* = 13.7, 6.9 Hz, 1H), 1.30 (s, 9H), 1.05 (dd, *J* = 4.1, 2.4 Hz, 3H); **$^{13}$C NMR (151 MHz, MeOD)** δ 158.33, 152.83, 128.77, 118.11,

112.00, 110.56, 69.55, 65.06, 54.70, 50.24, 34.18, 30.30, 25.53, 18.91, 18.75; **HRMS** (ESI) m/z: [M+H]+ calcd for $C_{17}H_{30}NO_2$: 280.2244, found 280.2271.

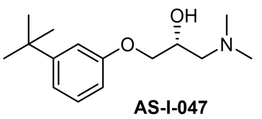

**AS-I-047**
**62**

**(R)-1-(3-(tert-butyl)phenoxy)-3-(dimethylamino)propan-2-ol (AS-I-047) (62):** Was synthesized according to general procedure B with epoxide **12** and dimethylamine as the amine and isolated as a clear oil (3.59 mg). **$^1$H NMR (500 MHz, MeOD)** δ 7.21 (t, *J* = 8.0 Hz, 1H), 7.02 (d, *J* = 7.8 Hz, 1H), 6.98 (t, *J* = 2.2 Hz, 1H), 6.76 (dd, *J* = 8.0, 2.6 Hz, 1H), 4.37 – 4.31 (m, 1H), 4.06 – 3.95 (m, 2H), 3.36 (d, *J* = 7.0 Hz, 2H), 2.96 (d, *J* = 14.4 Hz, 6H), 1.30 (s, 9H); **$^{13}$C NMR (151 MHz, MeOD)** δ 158.34, 152.83, 128.76, 118.13, 112.02, 110.52, 69.48, 63.94, 59.67, 44.19, 34.17, 30.30; **HRMS** (ESI) m/z: [M+H]+ calcd for $C_{15}H_{26}NO_2$: 252.1962, found 252.1958.

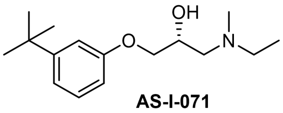

**AS-I-071**
**63**

**(R)-1-(3-(tert-butyl)phenoxy)-3-(ethyl(methyl)amino)propan-2-ol (AS-I-071) (63):** Was synthesized according to general procedure B with epoxide **12** and *N*-methylethanamine as the amine and isolated as a clear oil (1.42 mg). **$^1$H NMR (500 MHz, MeOD)** δ 7.21 (t, *J* = 8.0 Hz, 1H), 7.05 – 6.97 (m, 2H), 6.76 (d, *J* = 8.1 Hz, 1H), 4.35 (s, 1H), 4.07 – 3.96 (m, 2H), 3.48 – 3.35 (m, 1H), 2.94 (d, *J* = 12.6 Hz, 3H), 1.41 – 1.34 (m, 3H), 1.32 – 1.27 (m, 9H). **$^{13}$C NMR (151 MHz, MeOD)** δ 158.35, 152.83, 128.77, 118.13, 112.03, 110.56, 69.53, 63.77, 56.96, 52.85, 38.44, 34.18, 30.30, 8.02; **HRMS** (ESI) m/z: [M+H]+ calcd for $C_{16}H_{27}NO_2$: 266.2112 found 266.2114.

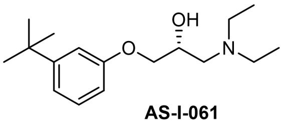

**AS-I-061**
**64**

**(R)-1-(3-(tert-butyl)phenoxy)-3-(diethylamino)propan-2-ol (AS-I-061) (64):** Was synthesized according to general procedure B with epoxide **12** and diethylamine as the amine and isolated as a clear oil (1.08 mg). **$^{1}$H NMR (500 MHz, MeOD)** δ 7.22 (t, *J* = 8.0 Hz, 1H), 7.05 – 6.97 (m, 2H), 6.77 (d, *J* = 8.2 Hz, 1H), 4.33 (s, 1H), 4.05 (t, *J* = 7.4 Hz, 1H), 4.02 – 3.96 (m, 1H), 3.38 (m, 6H), 3.32 (s, 9H), 1.36 (t, *J* = 7.2 Hz, 6H), 1.30 (s, 9H); **$^{13}$C NMR (151 MHz, MeOD)** δ 158.32, 152.85, 128.78, 118.15, 112.02, 110.61, 69.54, 64.14, 54.22, 49.03, 34.18, 30.30, 7.78; **HRMS** (ESI) m/z: [M+H]+ calcd for $C_{17}H_{30}NO_2$: 280.2277, found 280.2277.

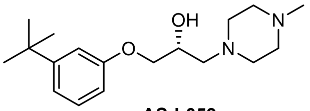

**AS-I-059**
**65**

**(R)-1-(3-(tert-butyl)phenoxy)-3-(4-methylpiperazin-1-yl)propan-2-ol (AS-I-059) (65):** Was synthesized according to general procedure B with epoxide **12** and *N*-methylpiperizine as the amine and isolated as a clear oil (4.68 mg). **$^{1}$H NMR (500 MHz, MeOD)** δ 7.19 (d, *J* = 8.4 Hz, 1H), 7.02 – 6.94 (m, 2H), 6.74 (d, *J* = 7.4 Hz, 1H), 4.12 (m, 1H), 4.02 – 3.95 (m, 2H), 2.81 (s, 8H), 1.30 (s, 9H); **$^{13}$C NMR (151 MHz, MeOD)** δ 158.68, 152.71, 128.68, 117.80, 112.04, 110.56, 69.90, 66.66, 59.33, 53.00, 48.16, 42.39, 34.15, 30.32; **HRMS** (ESI) m/z: [M+H]+ calcd for $C_{18}H_{31}N_2O_2$: 307.2392 found 307.2380.

**X-ray Structure Determination of Compound 8:**

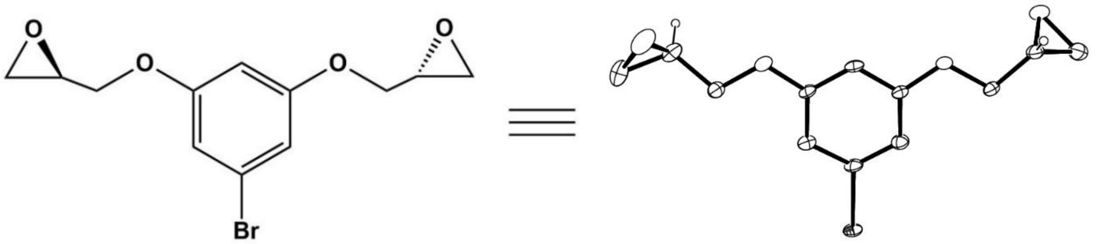


Compound **8** Crystallization and diffraction was performed by Michael Gau at the University of Pennsylvania X-Ray Crystallography Facility. Compound **8** , $C_{12}H_{13}BrO_4$, crystallizes in the monoclinic space group $P2_1$ (systematic absences 0k0: k=odd) with a=4.1597(3)Å, b=8.1637(5)Å, c=17.7262(12)Å, α=90°, β=93.123(6)°, γ=90°, V=601.06(7)$Å^3$, Z=2, and $d_{calc}$=1.664 g/$cm^3$. X-ray intensity data were collected on a Rigaku XtaLAB Synergy-S diffractometer equipped with an HPC area detector (Dectris Pilatus3 R 200K) and employing confocal multilayer optic-monochromated Mo-Kα radiation (λ=0.71073 Å) at a temperature of 100K. Preliminary indexing was performed from a series of thirty 0.5° rotation frames with exposures of 15 seconds. A total of 1212 frames (9 runs) were collected employing ω scans with a crystal to detector distance of 34.0 mm, rotation widths of 0.5° and exposures of 50 seconds.

Rotation frames were integrated using CrysAlisPro, producing a listing of unaveraged $F^2$ and $\sigma(F^2)$ values. A total of 11302 reflections were measured over the ranges $4.602 \le 2\theta \le 56.562°$, $-5 \le h \le 5$, $-9 \le k \le 10$, $-23 \le l \le 23$ yielding 2931 unique reflections ($R_{int}$ = 0.0651). The intensity data were corrected for Lorentz and polarization effects and for absorption using SCALE3 ABSPACK (minimum and maximum transmission 0.39964, 1.00000). The structure was solved by dual space methods - SHELXT. Refinement was by full-matrix least squares based on $F^2$ using SHELXL [5]. All reflections

were used during refinement. The weighting scheme used was w=1/[$\sigma^2(F_o^2)$+ $(0.0792P)^2$ + 0.0000P] where P = $(F_o^2 + 2F_c^2)/3$. Non-hydrogen atoms were refined anisotropically and hydrogen atoms were refined using a riding model. Refinement converged to R1=0.0456 and wR2=0.1102 for 2625 observed reflections for which F > 4σ(F) and R1=0.0526 and wR2=0.1130 and GOF =1.060 for all 2931 unique, non-zero reflections and 154 variables. The maximum Δ/σ in the final cycle of least squares was 0.000 and the two most prominent peaks in the final difference Fourier were +1.97 and -1.06 e/Å$^3$.

Table S2.1 lists cell information, data collection parameters, and refinement data. Final positional and equivalent isotropic thermal parameters are given in Tables S2.2 and S2.3 Anisotropic thermal parameters are in Table S2.4 Tables S2.5 and Tables S2.6 list bond distances and bond angles. Figure S5 is an ORTEP representation of the molecule with 50% probability thermal ellipsoids displayed.

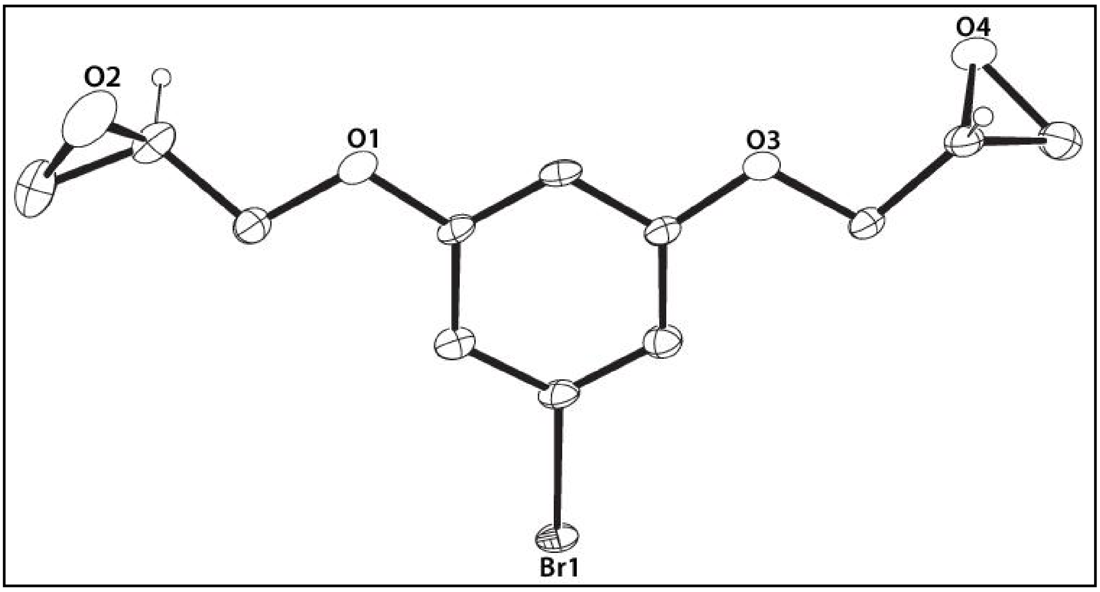


**Figure S5. ORTEP drawing of the title compound with 50% thermal ellipsoids.**

**Table S2.1. Summary of Structure Determination of Compound 8**

| | |
|---|---|
| Empirical formula | $C_{12}H_{13}BrO_4$ |
| Formula weight | 301.13 |
| Diffractometer | Rigaku XtaLAB Synergy-S (Dectris Pilatus3 R 200K) |
| Temperature/K | 100 |
| Crystal system | monoclinic |
| Space group | $P2_1$ |
| a | 4.1597(3)Å |
| b | 8.1637(5)Å |
| c | 17.7262(12)Å |
| α | 90° |
| β | 93.123(6)° |
| γ | 90° |
| Volume | 601.06(7)$Å^3$ |
| Z | 2 |
| $d_{calc}$ | 1.664 g/$cm^3$ |
| μ | 3.419 $mm^{-1}$ |
| F(000) | 304.0 |
| Crystal size, mm | 0.419 × 0.099 × 0.038 |
| 2θ range for data collection | 4.602 - 56.562° |
| Index ranges | -5 ≤ h ≤ 5, -9 ≤ k ≤ 10, -23 ≤ l ≤ 23 |
| Reflections collected | 11302 |
| Independent reflections | 2931[R(int) = 0.0651] |
| Data/restraints/parameters | 2931/1/154 |
| Goodness-of-fit on $F^2$ | 1.060 |
| Final R indexes [I>=2σ (I)] | $R_1$ = 0.0456, $wR_2$ = 0.1102 |
| Final R indexes [all data] | $R_1$ = 0.0526, $wR_2$ = 0.1130 |
| Largest diff. peak/hole | 1.97/-1.06 e$Å^{-3}$ |
| Flack parameter | 0.001(9) |

### Table S2.2. Refined Positional Parameters for Compound 8

| Atom | *x* | *y* | *z* | U(eq) |
|---|---|---|---|---|
| Br1 | 0.34187(11) | 0.81584(2) | 0.28144(3) | 0.02278(18) |
| O1 | 0.4546(11) | 0.1821(5) | 0.3272(3) | 0.0209(11) |
| O2 | 0.6248(13) | -0.0194(6) | 0.4779(3) | 0.0375(12) |
| O3 | -0.1505(10) | 0.3413(5) | 0.1098(2) | 0.020(1) |
| O4 | -0.2826(9) | 0.3126(8) | -0.06092(19) | 0.0263(7) |
| C1 | 0.2573(15) | 0.5936(7) | 0.2569(3) | 0.0190(12) |
| C2 | 0.3893(15) | 0.4744(8) | 0.3058(4) | 0.0180(13) |
| C3 | 0.3321(11) | 0.3087(15) | 0.2853(2) | 0.0164(10) |
| C4 | 0.1471(13) | 0.2694(8) | 0.2200(3) | 0.0177(12) |
| C5 | 0.0215(13) | 0.3943(7) | 0.1738(3) | 0.0159(10) |
| C6 | 0.0736(15) | 0.5575(7) | 0.1910(3) | 0.0193(11) |
| C7 | 0.6552(15) | 0.2245(8) | 0.3931(3) | 0.0205(12) |
| C8 | 0.8101(15) | 0.0747(8) | 0.4265(4) | 0.0248(13) |
| C9 | 0.8978(17) | 0.0766(10) | 0.5068(4) | 0.0352(16) |
| C10 | -0.2771(13) | 0.4686(7) | 0.0599(3) | 0.0166(10) |
| C11 | -0.4669(14) | 0.3861(8) | -0.0029(3) | 0.0200(11) |
| C12 | -0.4796(17) | 0.4545(8) | -0.0790(3) | 0.0268(13) |

### Table S2.3 Positional Parameters for Hydrogens in Compound 8.

| Atom | *x* | *y* | *z* | U(eq) |
|---|---|---|---|---|
| H2 | 0.511723 | 0.502995 | 0.350674 | 0.022 |
| H4 | 0.106751 | 0.158074 | 0.207092 | 0.021 |
| H6 | -0.012645 | 0.641949 | 0.158988 | 0.023 |
| H7A | 0.52316 | 0.277839 | 0.43087 | 0.025 |
| H7B | 0.823078 | 0.302973 | 0.378944 | 0.025 |
| H8 | 0.954092 | 0.011783 | 0.393638 | 0.03 |
| H9A | 1.095028 | 0.017076 | 0.524561 | 0.042 |
| H9B | 0.853263 | 0.177599 | 0.535362 | 0.042 |
| H10A | -0.099083 | 0.532929 | 0.039828 | 0.02 |
| H10B | -0.416487 | 0.543627 | 0.087424 | 0.02 |
| H11 | -0.661705 | 0.324224 | 0.011529 | 0.024 |
| H12A | -0.67933 | 0.43794 | -0.111047 | 0.032 |
| H12B | -0.369059 | 0.560068 | -0.086617 | 0.032 |

**Table S2.4. Refined Thermal Parameters (U's) for Compound 8**

| Atom | $U_{11}$ | $U_{22}$ | $U_{33}$ | $U_{23}$ | $U_{13}$ | $U_{12}$ |
|---|---|---|---|---|---|---|
| Br1 | 0.0314(3) | 0.0081(3) | 0.0286(3) | -0.0020(3) | -0.00018(18) | -0.0020(3) |
| O1 | 0.025(2) | 0.010(2) | 0.027(2) | 0.0027(19) | -0.0022(18) | -0.0017(18) |
| O2 | 0.039(3) | 0.028(3) | 0.047(3) | 0.017(2) | 0.008(2) | 0.001(2) |
| O3 | 0.0286(19) | 0.010(3) | 0.0213(17) | -0.0010(15) | -0.0032(14) | 0.0005(16) |
| O4 | 0.0326(19) | 0.0197(18) | 0.0266(17) | -0.005(3) | 0.0029(14) | 0.007(3) |
| C1 | 0.026(3) | 0.009(3) | 0.023(3) | 0.001(2) | 0.005(2) | -0.002(2) |
| C2 | 0.013(3) | 0.015(3) | 0.026(3) | -0.001(2) | 0.003(2) | -0.001(2) |
| C3 | 0.020(2) | 0.009(3) | 0.021(2) | 0.003(3) | 0.0038(17) | 0.000(3) |
| C4 | 0.022(3) | 0.008(3) | 0.024(3) | -0.0007(17) | 0.005(2) | -0.0014(18) |
| C5 | 0.015(2) | 0.012(3) | 0.020(3) | -0.001(2) | 0.003(2) | 0.003(2) |
| C6 | 0.023(3) | 0.014(3) | 0.021(3) | 0.001(2) | 0.003(2) | -0.001(2) |
| C7 | 0.023(3) | 0.017(3) | 0.021(3) | 0.001(2) | 0.001(2) | 0.003(2) |
| C8 | 0.024(3) | 0.020(3) | 0.031(3) | 0.005(2) | 0.006(2) | 0.005(2) |
| C9 | 0.031(4) | 0.039(4) | 0.035(4) | 0.012(3) | -0.005(3) | 0.007(3) |
| C10 | 0.016(2) | 0.012(3) | 0.022(3) | 0.001(2) | 0.0013(19) | 0.001(2) |
| C11 | 0.020(3) | 0.013(2) | 0.027(3) | -0.002(2) | -0.001(2) | 0.000(2) |
| C12 | 0.035(3) | 0.022(3) | 0.023(3) | -0.002(2) | 0.000(2) | 0.001(3) |

**Table S2.5. Bond Distances in Compound 8, Å**

| | | | | | |
|---|---|---|---|---|---|
| Br1-C1 | 1.894(6) | O1-C3 | 1.356(11) | O1-C7 | 1.440(8) |
| O2-C8 | 1.446(8) | O2-C9 | 1.451(9) | O3-C5 | 1.377(7) |
| O3-C10 | 1.445(7) | O4-C11 | 1.446(7) | O4-C12 | 1.444(8) |
| C1-C2 | 1.395(9) | C1-C6 | 1.393(9) | C2-C3 | 1.418(14) |
| C3-C4 | 1.391(8) | C4-C5 | 1.392(8) | C5-C6 | 1.381(8) |
| C7-C8 | 1.489(8) | C8-C9 | 1.451(10) | C10-C11 | 1.490(8) |
| C11-C12 | 1.458(9) | | | | |

**Table S2.6. Bond Angles in Compound 8, °**

| | | | | | |
|---|---|---|---|---|---|
| C3-O1-C7 | 116.4(6) | C8-O2-C9 | 60.1(4) | C5-O3-C10 | 115.7(4) |
| C12-O4-C11 | 60.6(4) | C2-C1-Br1 | 117.6(5) | C6-C1-Br1 | 118.8(4) |
| C6-C1-C2 | 123.6(6) | C1-C2-C3 | 116.8(6) | O1-C3-C2 | 122.3(5) |
| O1-C3-C4 | 117.0(9) | C4-C3-C2 | 120.7(8) | C3-C4-C5 | 119.5(7) |
| O3-C5-C4 | 114.6(5) | O3-C5-C6 | 123.5(5) | C6-C5-C4 | 121.9(5) |
| C5-C6-C1 | 117.4(5) | O1-C7-C8 | 110.1(5) | O2-C8-C7 | 116.9(5) |
| O2-C8-C9 | 60.1(4) | C9-C8-C7 | 117.5(6) | O2-C9-C8 | 59.8(4) |
| O3-C10-C11 | 107.0(5) | O4-C11-C10 | 116.1(5) | O4-C11-C12 | 59.7(4) |
| C12-C11-C10 | 120.6(6) | O4-C12-C11 | 59.8(4) | | |
